# The Chemical Reactivity of Membrane Lipids

**DOI:** 10.1021/acs.chemrev.3c00608

**Published:** 2024-03-18

**Authors:** Genevieve Duché, John M Sanderson

**Affiliations:** ∇ Génie Enzimatique et Cellulaire, Université Technologique de Compiègne, Compiègne 60200, France; † Chemistry Department, Durham University, Durham DH1 3LE, United Kingdom

## Abstract

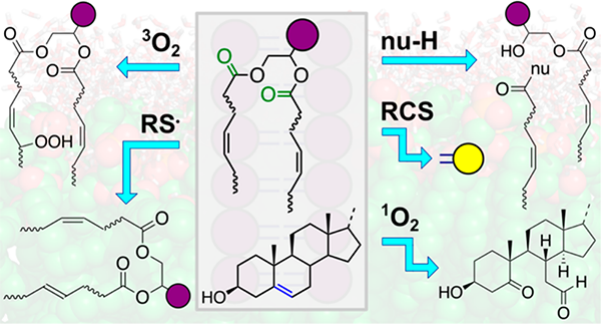

It is well-known that aqueous dispersions of phospholipids spontaneously assemble into bilayer structures. These structures have numerous applications across chemistry and materials science and form the fundamental structural unit of the biological membrane. The particular environment of the lipid bilayer, with a water-poor low dielectric core surrounded by a more polar and better hydrated interfacial region, gives the membrane particular biophysical and physicochemical properties and presents a unique environment for chemical reactions to occur. Many different types of molecule spanning a range of sizes, from dissolved gases through small organics to proteins, are able to interact with membranes and promote chemical changes to lipids that subsequently affect the physicochemical properties of the bilayer. This Review describes the chemical reactivity exhibited by lipids in their membrane form, with an emphasis on conditions where the lipids are well hydrated in the form of bilayers. Key topics include the following: lytic reactions of glyceryl esters, including hydrolysis, aminolysis, and transesterification; oxidation reactions of alkenes in unsaturated fatty acids and sterols, including autoxidation and oxidation by singlet oxygen; reactivity of headgroups, particularly with reactive carbonyl species; and *E*/*Z* isomerization of alkenes. The consequences of reactivity for biological activity and biophysical properties are also discussed.

## 1 Introduction

### 1.1 Overview

The structure of bilayers formed by amphipathic lipid molecules has been the subject of intense study since the pioneering work on the nature of the biological membrane by Danielli, Davson, and Robertson in the first half of the 20th century.^1−3^ However, it was not until the 1960s, with the realization by Bangham that lipids can spontaneously form liposomes that exhibit many of the properties of the biological membranes, that the properties of the lipid membrane began to be fully understood. Since then there has been an explosion of uses and applications of liposomes, from drug delivery agents and tools for studying the physical properties of membranes to models for biological membranes.^4−7^ Since 2010 there have been >36 000 primary articles (Web of Science search, Topic = liposom*, type = article, range 2010 to 2019; accessed 2022-05-25) and >14 000 patents (Espacenet search, https://worldwide.espacenet.com, worldwide search, Title, Abstract, or Claims contain “liposom*”, 2010 to present; accessed 2023-05-09) involving the use of liposomes. For almost as long as liposomes have been used, the chemical and physical stability of membranes, both biological and synthetic, has been examined in order to understand how the membrane responds to changes in the physical and chemical environment and how the products of reactions involving lipids can exert biological activity. Although many aspects of membrane stability are now well characterized, our understanding of lipid chemistry is still evolving.

**Figure 1:**
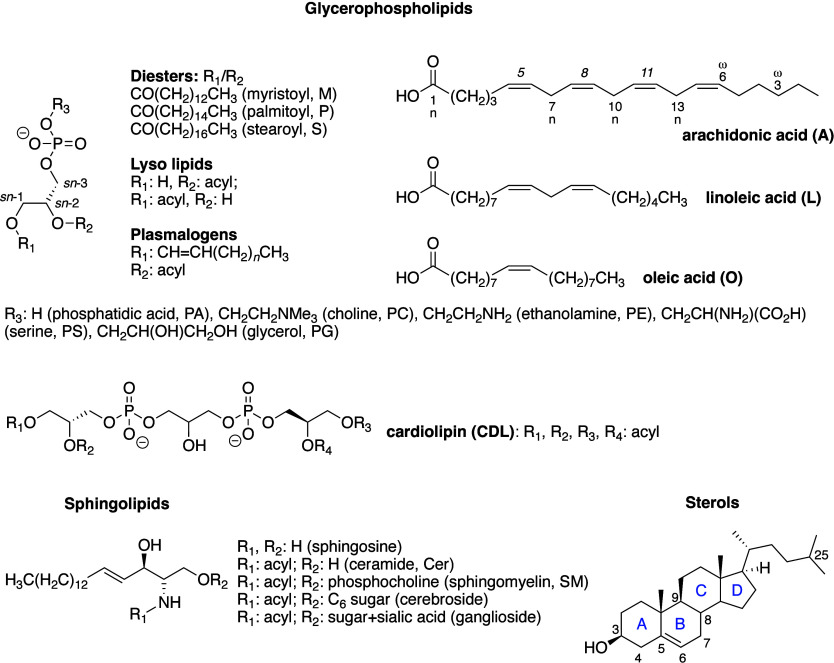
Structures of phospholipids described in this review. Numbering corresponds to common nomenclature.

Biological membranes comprise a range of phospholipid species that are summarized in Figure 1.^8,9^ The major glycerophospholipids are diesters of glycerol and are primarily classified according to the identity of the headgroup and the fatty acyl chains. Related classes of lipids, grouped together under the term plasmalogens, contain ether-linked alkyl chains. Sphingolipids are based around a ceramide core and are notable for an amide-linked acyl group that significantly increases both their chemical stability and the mechanical stability of membranes that incorporate them. Cardiolipins (CDLs) are notable for containing two diacylglycerophosholipids linked through glycerol to form a structure with four acyl chains. These lipids are found in the membranes of prokaryotes and the mitochondrial membranes of eukaryotes. Sterols, most notably cholesterol, significantly change the fluidity and phase behavior of membranes.^10−22^

In biological membranes, which are rich in unsaturated lipids at the *sn*-2 position and are generally in a fluid liquid crystalline phase under physiological conditions, cholesterol incorporation increases their physical stability,^23−25^ decreases the lateral diffusion rate,^19,26−28^ and reduces the permeation of water and hydrophilic solutes across the bilayer,^29−37^ although the level of hydration in the interfacial region is increased to the depth of the acyl carbonyl groups.^38−40^ For amphiphilic organic molecules and peptides, the effects of cholesterol on permeability and partitioning vary with membrane composition and the nature of the partitioning molecule.^41−44^

Permeation and partitioning are of key significance for the reactivity of membrane lipids, as the regions around the glycerol esters and fatty acid olefin groups are major sites of reactivity. The bilayer permeability of hydrogen peroxide is low, but increases if the content of unsaturated lipids increases. Its concentration is greatest near the outer leaflet (extracellular space) and decreases across the membrane toward the inner leaflet.^45^ Oxygen partitions favorably into membranes to give higher steady state concentrations in the bilayer than the surrounding aqueous phase. The presence of cholesterol reduces the favorability of oxygen partitioning.^46^ In a similar vein, the permeability of dissolved gases, including oxygen^47,48^ and carbon dioxide,^49^ is decreased by the presence of cholesterol. In some systems, however, the presence of cholesterol has been found to increase oxygen permeability by an order of magnitude.^50^

In mixtures containing cholesterol that exhibit phase separation into liquid ordered (L_o_) and liquid disordered (L_d_) phases, the L_o_ phase has lower water permeability.^29,51^ However, the difference in water permeability between L_o_ and L_d_ is significantly lower than the difference between the permeability of gel and fluid phases of single-component lipid membranes. This difference is attributed to a permeation pathway that includes diffusion into the L_d_ regions at the L_o_/L_d_ boundary with subsequent diffusion along the membrane midplane.^52^

The composition of biological membranes, in terms of lipid type, class, and acyl composition, varies significantly by organism, cell type, and organelle.^8,9^ Furthermore, some cells are able to adapt the lipid profile of their membranes in response to changes in the local environment.^53−55^ Within many membranes, the distribution of lipids is nonuniform, both laterally within the plane of membrane,^56−59^ and transversely between leaflets.^20,26,60^ For example, in eukaryotes the plasma membrane is enriched in phosphatidylethanolamine (PE), phosphatidylserine (PS), and phosphatidylinositol (PI) in the inner leaflet and phosphatidylcholine (PC), sphingolipids, and cholesterol in the outer plasma membrane leaflet.^20,60−63^ Mitochondrial membranes also exhibit lateral and transverse asymmetry.^64,65^ Synthetic liposomes, especially when prepared in bulk in the absence of extrinsic reagents, are transversely symmetric but can still exhibit lateral separation into domains with separate phases.^19,66,67^ The formation of more ordered phases, frequently termed lipid rafts, has been extensively studied in ternary systems, particularly those involving PC, sphingolipids, and cholesterol.^56,58,68,69^

Reactions of membrane lipids, such as oxidation and hydrolysis, that lead to significant chemical changes have been well reviewed.^70−75^ This is especially true in the field of food science,^70−72^ as oxidation of lipids can seriously impact the quality of commercial food and beverage products.

### 1.2 Biological Membranes

Liposomes have been valuable tools for relating the effects of chemical changes in membrane lipids to the corresponding changes in fundamental membrane properties, such as hydration, porosity, compressibility, lateral diffusion, and transverse diffusion (flip-flop).^6,76−79^ Studies *in vitro* have also proved useful for the development of assays to detect the same modifications *in vivo*, providing insights into the occurrence and consequences of lipid damage to biological membranes.^80−86^ The development of methods in mass spectrometry, particularly with regard to cellular lipidomic profiling,^87−93^ and matrix assisted laser desorption/ionization (MALDI) imaging^94−96^ is providing particularly powerful tools for revealing changes in lipid composition during aging and the progression of numerous diseases, including cardiovascular diseases, cancer, diabetes, and neurodegenerative diseases.^80,97−100^

Some of the chemical reactions that lipids undergo, most notably nonenzymatic hydrolysis, occur at significantly slower rates than most biological processes.^101^ As a result, in a healthy cell the products of lipid reactivity do not accumulate. The levels of lipid degradation products *in vivo* are intrinsically linked to homeostasis and cell signaling, and the levels are therefore controlled.^54,102−111^ This is particularly true for oxidation products that arise from reaction with reactive oxygen species (ROS). ROS are generated *via* the normal pathways of oxidative degradation that occur within mitochondria, mostly *via* reverse electron transport and flavin mononucleotide pathways.^112−114^ Under stressed conditions, such as during disease or following injury, increased levels of lipid degradation products are associated with the resulting physiological response.^115^ Elevated levels of lipid oxidation products have been implicated in degenerative neurological conditions such as Alzheimer’s^116−120^ and Parkinson’s diseases,^118,121,122^ as well as diseases that involve the overproduction of ROS such as type 2 diabetes^123,124^ and cancer,^109,113,125^ among others.^115,126−128^ Increased levels of oxidation products are found following traumatic brain injury^96,129−131^ and are also a marker of aging.^117^

Many lipid degradation products, particularly those arising from ROS, chemically modify other biological macromolecules such as proteins and nucleic acids.^132−137^ Such modifications have been implicated, for example, in the pathology of Parkinson’s disease through the modification of the protein α-synuclein at internal lysine residues by the byproducts of lipid peroxidation.^138^

The interplay between chemical damage to a particular class of lipid and homeostasis can be complex. For example, oxidative damage to glycerophosphocholine lipids by ROS changes the biophysical properties of the membrane, leading to activation of phospholipases that then catalyze lipid hydrolysis to form byproducts which themselves are involved in cell signaling.^100,139,140^ The purpose of this response, principally mediated by cPLA_2_, is to recycle arachidonic acid, which is particularly prone to oxidative damage.^100,131,141^ Conversely, loss of mitochondrial phospholipase activity leads to increased lipid peroxidation in this organelle.^142^ This complexity is not restricted to lipid class. Sphingomyelinases (SMases), for example, catalyze the formation of phosphatidylcholine from PA and sphingomyelin, producing ceramide as a byproduct.^20,143,144^ SMase activity increases in the presence of ROS.^145,146^ Ceramide and the related sphingolipid sphingosine-1-phosphate perform key roles in cellular homeostasis and apoptosis.^147,148^ The exchange between ceramide and sphingomyelin may also be used to modulate the lateral phase behavior of membranes.^149^ As a final point, redox-active metals such as Fe(II) produce radical initiators from ROS and facilitate the breakdown of lipid hydroperoxides. When the usual corrective enzyme for lipid hydroperoxides is absent, the generation of excess lipid hydroperoxides leads to cell death by a nonapoptotic route that is Fe(II)-dependent (ferroptosis).^106^

### 1.3 Drug Discovery and Delivery

Liposomes have been extensively examined as systems that can be used to deliver pharmaceutical agents^150−153^ and macromolecules such as proteins^154,155^ and nucleic acids^4,156,157^ that would otherwise be unstable. Due to the amphipathic nature of lipids, the principal uses of liposomes are to solubilize hydrophobic drugs or to encapsulate hydrophilic drugs that would otherwise be unstable *in vivo*. Liposomes can enhance tissue penetration and retention passively and can be decorated with groups that target them to specific tissues or cells,^158−161^ and many drugs become more biocompatible when incorporated in liposomes, reducing adverse reactions and improving their therapeutic index.^151,162^ For all these applications it is essential that lipids exhibit good chemical stability, both *in vivo* and during preparation, sterilization, and storage in the formulated form prior to administration,^71,163^ particularly since the byproducts of lipid breakdown can exhibit acute toxicity.^164,165^ The stability of encapsulated macromolecules can also be influenced by changes in the chemical composition of the membrane, especially if the macromolecule can bind hydrolysis products. For example, the stability of human serum albumin (HSA) is improved in aged 1,2-dipalmitoyl-*sn*-glycero-3-phosphocholine (DPPC)/CDL liposomes, an effect attributed to the association of HSA with free fatty acids (FFAs) formed by hydrolysis.^166^

Liposomes are frequently modified with surface groups that improve bioavailability and *in vivo* stability^159,160^ Approaches used to improve stability include the use of modified lipids to change the surface activity,^167,168^ incorporation of polymeric materials, and the formation of colloidal dispersions such as solid lipid nanoparticles (SLNs).^169,170^ As biomembrane mimics, liposomes have been used to model drug-lipid interactions and pharmacokinetic behavior.^5,171,172^

### 1.4 Food

As membranes are ubiquitous in biology, many of the chemical processes that occur in liposomes can also occur during food processing. This is of particular concern because the usual cellular mechanisms for removing lipid byproducts no longer operate in many cases. This can lead to the accumulation of products that degrade taste, smell, visual appearance, and nutritional quality. In addition to the lipids found naturally in foods, liposomes are used in food processing to deliver molecules that enhance flavor and nutritional content, improve shelf life,^173−176^ or reduce exposure to proteolytic enzymes in the gastrointestinal tract.^177^ Consequently, a large body of literature has examined the chemical changes that occur in food during processing, packaging, and transport.^70,72,153,178−181^

### 1.5 Cosmetics

Liposomes are primarily used in cosmetics to improve the stability of components within the formulation or to enhance the delivery and skin penetration of materials such as antioxidants.^182−185^ As with drug delivery, this exploits the ability of liposomes to solubilize hydrophobic materials and entrap water-soluble actives. When applied topically, entrapment of actives with liposomes brings the dual advantages of increasing skin localization and reducing systemic distribution, as well as improving the bioavailability of the target drugs.^186,187^ Liposome incorporation into products such as skin creams often brings additional benefits, such as improvements in skin hydration.^184,185,188,189^

The FDA guidelines for industry, issued in April 2018 (https://www.fda.gov/media/70837/download, accessed 2023-06-23), make it a requirement that for any liposome formulation including synthetic lipids, the levels of products associated with lipid degradation are quantified, including lysophospholipids, free fatty acids, and peroxides. In addition, stress testing of the formulation is required to examine physicochemical stability under extremes of temperature, light, pH, and oxygen in order to determine optimal storage conditions and retest periods. From the extensive literature on lipid reactivity, it is apparent that, when stored at ambient or low temperatures at neutral pH, most liposomes are remarkably stable for lengthy periods, frequently exhibiting little chemical change over a period of several months.

### 1.6 Key Challenges for Studying the Reactivity of Membrane Lipids

There are two key obstacles to understanding the chemical reactivity of membrane lipids. First, lipids are challenging to manipulate due to their properties of limited solubility in many solvents, long retention times in both normal and reversed phase chromatography, and formation of heterogeneous phases in aqueous systems. Second, the products of lipid reactivity often have significantly different properties compared to lipids, generally being more polar and sometimes even volatile. In recent years there have been spectacular advances in the analysis of lipid reaction products due to advances in mass spectrometry (MS), together with liquid chromatography (LC), and direct infusion “shotgun” approaches,^190−192^ which have enabled the highly sensitive detection of trace components of lipid mixtures and thus revealed a wealth of diverse lipid chemistry.^87,88^ While synthetic systems such as liposomes and solid lipid nanoparticles are well described chemically when prepared fresh and therefore changes in composition in controlled conditions are easily related to chemical reactivity, particularly when instruments can be calibrated against authentic standards, the challenge is significantly greater in biological systems. The cell lipidome is complex and includes several examples of lipids of the same mass but differing fatty acid composition. Many of the more polar products of lipid reactivity also ionize much more readily than lipids themselves, leading to the detection of products of such low abundance that any chemical or biological activity is uncertain. Tandem mass spectrometry approaches can decipher the fatty acid composition of individual lipids, but it is not a trivial pursuit.

In most cells there is a continual turnover of lipids and therefore the composition of many cellular membranes, both in terms of lipid class and acyl group composition, can evolve over time. Consequences of this evolution are a changing reference point or control alongside changes in the distribution of reaction products. Some products of lipid chemical reactivity, such as fatty acids and lysolipids, are produced by processes that occur both with and without enzyme control, making it challenging to establish which mode of generation is most significant. Furthermore, some products of lipid lysis are themselves substrates for other enzymes and can change receptor activation, either by direct binding or indirectly through changes in membrane properties. Changes in the levels of these products are therefore challenging to monitor in order to establish cause and effect. Some products of lipid reactivity, such as oxidation products, trigger large physiological responses such as ferroptosis that produce significant additional complexity.

The purpose of this Review is to provide an update on our current understanding of the stability of all the classes of lipid commonly found in biological and synthetic membranes. It focuses on the chemical stability of lipids in their membrane form, as found in liposomes and biological membranes, with an emphasis on conditions in which the lipids are well hydrated in the form of bilayers.

## 2 Hydrolysis

This section is concerned with the chemical (*i.e.*, non-enzyme-catalyzed) hydrolysis of glycerophospholipids, as this is by far the most significant class for which hydrolysis has been studied. Enzyme-catalyzed hydrolysis of glycerophospholipids is discussed for context. No significant reports exist of the chemical hydrolysis of sphingolipids or sterols.

### 2.1 Phospholipases

Before considering hydrolysis reactions of lipids in depth, it is useful to briefly consider the reactions catalyzed by phospholipases, as many of the reactions they catalyze are the same as hydrolysis reactions seen *in vitro* and, as mentioned earlier, the activity of these enzymes is often invoked in response to other types of chemical reactivity *in vivo*, such as oxidation. Many of the products of phospholipase activity are themselves biologically active, acting as second messengers in cell signaling.

**Figure 2:**
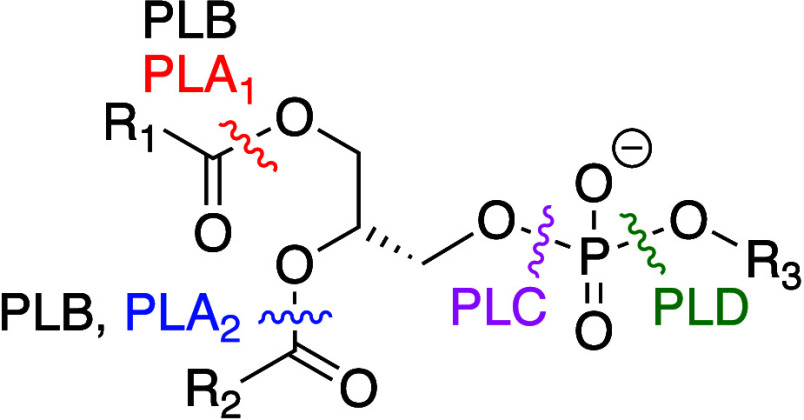
Glycerophospholipid bonds cleaved by phospholipases A–D.

Phospholipases have been well reviewed.^61,193−195^ They act upon glycerophospholipids to generate hydrolysis products (Figure 2, Table 1). They are classified according to the bond hydrolyzed: phospholipases A (PLAs) hydrolyze the carboxyl esters, with PLA_1_ (frequently just termed “lipase”) and PLA_2_ selective for the *sn*-1 and *sn*-2 acyl groups, respectively; phospholipase C (PLC) hydrolyzes the phosphate ester on the glyceryl side to form a diacylglycerol and a phosphate ester of the headgroup; and phospholipase D (PLD) hydrolyzes the phosphate ester on the headgroup side to form a phosphatidic acid and the free headgroup (serine, ethanolamine, choline, glycerol, or inositol).

**Table 1:** Overview of the Chemical Reactivity of Phospholipases

enzyme	product(s)	major biological activity
PLA_1_	free fatty acid	Saturated FFAs (typically found at the glycerol *sn*-1 position) increase lipid disorder and increase membrane permeability^203,204^
	2-acyl-glycerophospholipid	changes in membrane fluidity, bending modulus, and barrier integrity;^203,205−207^ Signaling during cell growth (lyso-PI);^110,207^ and inflammation (lyso-PC)^208^
PLA_2_	free fatty acid	polyunsaturated fatty acids (PUFAs) are involved in the inflammatory response and are precursors for second messengers in the CNS;^209,210^ unsaturated FFAs have a greater membrane perturbing effect than saturated FFAs^203,204^
	1-acyl-glycerophospholipid	see 2-acyl-glycerophospholipid
PLB	free fatty acid	see above
	*sn*-glycero-3-phosphoalcohol	regulation of G-proteins (glycero-PI)^207^
PLC	diacylglycerol	activates protein kinase C^211^
	3-phosphoalcohol	inflammatory response by activation of C-reactive protein (phosphocholine);^212^ cell signaling (inositol phosphates);^213,214^ others are involved in general metabolism or are inert
PLD	phosphatidic acid	cell signaling (second messenger)^197,215−217^
	lipid headgroup (as alcohol)	general metabolism

PLA_1_ and PLA_2_ both selectively form a FFA and a lysolipid. The lysolipid may itself be subject to further hydrolysis catalyzed by phospholipase B (PLB), which does not exhibit the *sn*-1/*sn*-2 selectivity of PLA. Some PLAs, such as cPLA2, also exhibit lysophospholipase activity.^141,196^ During chemical analysis of lipid membranes, the lysolipids formed by PLA_1_ and PLA_2_ activity typically give an equilibrium mixture of products that result from acyl migration between the *sn*-1 and *sn*-2 positions, with the *sn*-1 acyl being the major product. PLA_1_, PLA_2_, and PLD also exhibit transesterification activities, providing a means for exchanging the acyl groups in the case of the former two and the headgroup in the case of the latter.^197^ Non-PLA transesterifcation mechanisms have also been described for both glycerophospholipids^198,199^ and mitochondrial cardiolipins.^200^ Deacylation and reacylation reactions are controlled as part of the Lands’ cycle.^198,201,202^

### 2.2 Nonenzymatic Glycerophospholipid Hydrolysis

**Scheme 1:**
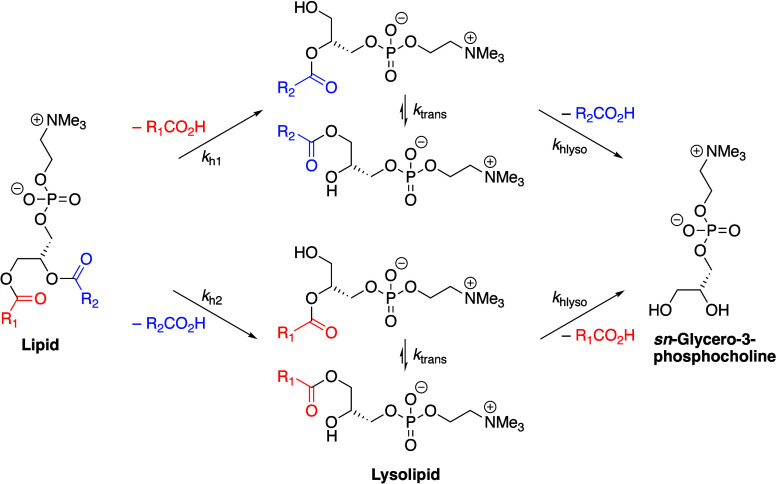
Ester Hydrolysis Reactions of PCs

Extensive studies of lipid hydrolysis were reported in the 1980s and 1990s, most notably by the group of Crommelin and subsequently by Zhang, using PC liposomes as model systems.^218−224^ These studies, and many others, have demonstrated that phosphate ester hydrolysis in aqueous dispersions is so slow as to be insignificant on any reasonable time scale. Hydrolysis of the carboxylic esters (Scheme 1) is the most significant process for membrane lipids.

#### 2.2.1 Temperature and pH Effects

Hydrolysis of the glyceryl esters follows pseudo-first-order kinetics for the decrease in lipid concentration, down to 25% lipid remaining, with a V-shaped pH-rate profile.^218,219,222,223,225−230^ The slowest rates are at pH 5.8–6.5, with hydrolysis at the *sn*-1 and *sn*-2 positions having comparable rates (*k*_h1_ and *k*_h2_, respectively, Scheme 1), typically 8.5 × 10^–4^ h^–1^ in neutral single-component PC membranes. This rate of hydrolysis corresponds to a half-life of >30 days at room temperature. At low temperatures (4–6 °C), and in the absence of buffer additives, the half-life for hydrolysis is often >200 days (Table 2). The major contribution to hydrolysis is specific acid–base catalysis,^218^ but buffer catalysis also occurs, with acetate, tris, and citrate ions all providing general acid–base catalysis.^223^ During the course of the reaction, the production of free fatty acid (FFA) lowers the pH,^231^ and this pH change is frequently used as a means of monitoring hydrolysis rates. The lysolipid that initially forms following hydrolysis is subject to a transesterifcation reaction, resulting in acyl migration to form predominantly the 1-acyl species regardless of the initial site of hydrolysis. The rate of this transesterification (*k*_trans_) has a minimum at a bulk pH of 4–5.^232^ Second-order rate constants for acid- and base-catalyzed transesterification have been estimated to be 4 × 10^–4^ and 160 M^–1^ s^–1^, respectively, with the first-order uncatalyzed reaction having a rate of 8 × 10^–7^ s^–1^.^232−234^ It is notable though, that the estimated rate of the acid-catalyzed reaction was determined at pH 1–2, which is close to the p*K*_a_ of the phosphate group (0.8).^235^ The rate of the acid-catalyzed reaction may be faster at acidic pH values significantly greater than 0.8. As second-order rate constants for hydroxide-catalyzed hydrolysis are typically 0.3 M^–1^ s^–1^,^222^ base-catalyzed transesterification is faster than hydrolysis by three orders of magnitude. Conversely, in acidic conditions, hydrolysis (rate constant of 1.6 × 10^–2^ M^–1^ s^–1^) is faster by two orders of magnitude.

**Table 2:** A Summary of Studies to Examine the Rates of Lipid Hydrolysis in Model Systems

lipid	*T* (°C)	Phase	pH	notes	*E*_a_ (kJ mol^–1^)^a^	*k*_obs_ (h^–1^)^b^	*t*_1/2_ (days)	ref
soybean PC	72	L_α_	4	buffer-free		7.7 × 10^–3^	3.7	(223)
			6.5			7.3 × 10^–4^	39.6	
			9			7.7 × 10^–3^	3.8	
soybean PC	72	L_α_	4	buffer (acetate/citrate/tris), μ = 0.068, *k*_w_ = 8.5 × 10^–4^ h^–1^	29.7	8.6 × 10^–3^	3.4	(223)
			6.5	*k*_H_ = 0.8 × 10^2^ M^–1^ h^–1^	57.2	1.7 × 10^–3^	17.2	
			8	*k*_OH_ = 6.8 × 10^2^ M^–1^ h^–1^	41.9	5.3 × 10^–3^	5.4	
				*k*_buff_ = 4.2 × 10^–3^ to 1.1 × 10^–1^ M^–1^ h^–1^ (except for AcOH)				
hydrogenated soy PC	70	L_α_?	4	50 mM buffer		7.3 × 10^–3^	4.0	(226)
			6			3.9 × 10^–4^	74.5	
			9			4.6 × 10^–3^	7.1	
egg PC/chol (7:1)	30	L_o_	4	citrate buffer (200 mM and 800 mM), *E*_a_ data are given here for 200 mM buffer, O_2_-free	56.2	2.1 × 10^–4^	137.0	(218)
	40		4		80.1	4.2 × 10^–4^	68.6	
	50		4		4.8	7.4 × 10^–4^	39.1	
	30		4.8			4.9 × 10^–5^	591.4	
	40		4.8			1.2 × 10^–4^	235.8	
	50		4.8			3.5 × 10^–4^	82.0	
bovine heart plasmenylcholine (semisynthetic)	38	gel/L_α_	1.57	NaCl (150 mM); buffers (20 mM): phosphate (pH 5.3), citrate (pH 4.3), unbuffered for pH 2.53 and 1.57 (pH adjusted with HCl); *T*_m_ = 37–38 °C		1.8	0.02	(242)
			2.53			0.6	0.05	
			4.3			2.3 × 10^–2^	1.27	
			5.3			2.9 × 10^–3^	9.9	
DPPlsC	37	?	4.5	DPPlsC is a di-*O*-((*Z*)-1′-hexadecenyl)-PCpl; citrate (20 mM), NaCl (150 mM).		0.23	0.1	(243)
DPPC/DOPE (3:1)	70	L_α_	4	DPPC hydrolysis; 20 mM lipid, 500 mM buffer (phosphate/acetate/HEPES)		3.6 × 10^–3^	8.0	(226)
			5.8			1.3 × 10^–3^	22.7	
			9.1			1.5 × 10^–2^	1.9	
DPPC/DOPE (3:1)	70	L_α_	4	DOPE hydrolysis; 20 mM lipid, 500 mM buffer (phosphate/acetate/HEPES)		4.4 × 10^–3^	6.6	(226)
			5.8			2.4 × 10^–3^	12.3	
			9.1			3.3 × 10^–2^	0.9	
DOTAP/DOPE (1:1)	70	?	4	DOTAP hydrolysis; 20 mM lipid, 500 mM buffer (phosphate/acetate/HEPES)		4.4 × 10^–3^	6.6	(226)
			5.9			3.7 × 10^–3^	7.7	
			7.9			6.9 × 10^–2^	0.4	
DOTAP/DOPE (1:1)	70	?	4	DOPE hydrolysis; 20 mM lipid, 500 mM buffer (phosphate/acetate/HEPES)		4.4 × 10^–3^	6.6	(226)
			5.9			4.8 × 10^–3^	6.0	
			7.9			6.7 × 10^–2^	0.4	
DOTAP/chol/DPPC (5:10:85)	50	L_o_?		DPPC hydrolysis; pH, buffers, and ionic strength not stated.		3.7 × 10^–3^	7.7	(244)
DOTAP/cholesterol/DPPC (5:10:85)	50	L_o_?		DOTAP hydrolysis; pH, buffers, and ionic strength not stated.		1.3 × 10^–3^		(244)
Lipoid E80	40	?	9.4	containing itraconazole microcrystals; unbuffered, pH 9.4 at start, pH 4 at end		7.2 × 10^–4^	40.1	(231)
Lipoid E80	40	?	9.4	containing itraconazole microcrystals; unbuffered, containing 0.22% (w/w) oleic acid, pH 9.46 at start, pH 7 at end		2.9 × 10^–4^	143.2	(231)
DPPC/DSPE-PEG_2000_ (90:4)	4	?	2	citrate buffer (300 mM); DPPC hydrolysis monitored		3.1 × 10^–4^	94.4	(229)
	4		4			6.5 × 10^–5^	445.7	
	22		2			1.8 × 10^–3^	16.2	
	22		4			2.1 × 10^–4^	136.0	
Lipoid E80	50	n/a		oil in water emulsion following autoclaving (20 min, 121 °C); unbuffered, pH 6.1 before autoclaving, pH 5.0 after 3 months.		6.9 × 10^–4^ (PC)	42.0	(236)
						5.6 × 10^–4^ (PE)	51.3	

a *E*_a_ modeled by Arrhenius kinetics over a range of temperatures.

b *k*_obs_ is the observed rate. *k*_obs_ =*k*_w_ + *k*_H_[H^+^] + *k*_OH_[OH^–^] + *k*_buff_[buffer], where *k*_w_ is first-order rate constant for hydrolysis in water and *k*_H_, *k*_OH_, and *k*_buff_ are the second-order rate constants for acid-catalyzed, base-catalyzed, and buffer-catalyzed processes, respectively).

Further hydrolysis of the lysolipid ultimately forms *sn*-glycero-3-phosphocholine (GPC).^220^ Most hydrolysis studies focus on either direct measurement of lysolipid or FFA formation or indirect measurements of hydrolysis such as pH changes. The hydrolysis of the lysolipid to FFA and GPC is infrequently addressed. However, a number of studies have produced kinetic profiles that reveal an initial increase in lysolipid levels, with a subsequent decrease at longer time periods that can be attributed to GPC formation.^229,236^ GPC formation has been measured directly in a small number of cases.^222,237^

Above the temperature of the main gel to liquid crystalline phase transition (*T*_m_), the temperature dependence of the rate exhibits Arrhenius kinetics, with activation energies (*E*_a_) in the range 40–80 kJ mol^–1^.^223,238,239^ For PC lipids with saturated chains, activation energies increase in the gel phase, resulting in a discontinuity in plots of rate vs 1/*T* at the transition temperature of the lipid.^219,240^ For drug delivery applications, gel phase membranes tend to be more stable in blood plasma, with DPPC/1,2-dipalmitoyl-*sn*-glycero-3-phosphoglycerol (DPPG), for example, being more stable than hen egg phosphatidylcholine (EPC)/hen egg phosphatidylglycerol (EPG), as assessed by the retention of radiolabeled DPPC within the liposome fraction (49% of a tritium label in the acyl group was retained within EPC/EPG after 48 h compared to 80% in DPPC/DPPG). Inclusion of the drug temoporfin did not significantly affect stability.^241^

#### 2.2.2 Chain Length Effects

In cholesterol-free fluid membranes above *T*_m_, shorter acyl chains and increased levels of unsaturation both yield faster hydrolysis rates, effects probably related to water penetration into the interface.^227^ In bicelles composed of 1,2-dimyristoyl-*sn*-glycero-3-phosphocholine (DMPC) and 1,2-dihexanoyl-*sn*-glycero-3-phosphocholine (DHPC), both saturated lipids, this is reflected by faster hydrolysis of the shorter chain lipid, which segregates to the edge of the bicelle.^245,246^ Cholesterol inclusion into fluid membranes slows hydrolysis,^218^ most likely as a consequence of an increased packing density in the liquid-ordered (*L*_o_) phase relative to the fluid phase and therefore reduced water penetration. For membranes composed of saturated lipids, such as 1,2-distearoyl-*sn*-glycero-3-phosphocholine (DSPC) and DPPC, chain length has little effect on rate for chain lengths between 12 and 18 carbons.^219,247^ Gel-phase membranes below *T*_m_ tend to exhibit slower hydrolysis than their counterparts that include cholesterol, particularly when the cholesterol content is high. For example, the activation energy for hydrolysis of DPPC/cholesterol (chol) (5:2) is lower than that of DPPC alone.^219^ In this case, the formation of the L_o_ phase has the potential to increase water penetration into the bilayer relative to the more densely packed gel phase. However, the effects of cholesterol incorporation on the hydrolysis kinetics of membranes composed of saturated lipids are small,^222^ and in many systems the inclusion of cholesterol imparts significant stability benefits with regard to hydrolysis. Some of these benefits may relate to specific interactions with components of the membrane such as PE,^248^ others to secondary effects resulting from the ability of cholesterol to decrease oxidative damage, particularly as the formation of oxidized species increases water penetration into the membrane and can thereby increases hydrolysis rates.^249−251^

In liposomes composed of DSPC/DPPC/1,2-dipalmitoyl-*sn*-glycero-3-phosphoglycerol (DSPG)/chol (35:35:20:10) and DSPC/DPPC/DSPG (38:38:24), qualitative detection of lysolipid was only possible in either system after 3 months at ambient temperatures.^252^ The commercial liposome preparation DOXIL, containing hydrogenated soybean PC (H-soyPC), cholesterol, and 1,2-distearoyl-*sn*-glycero-3-phosphoethanolamine–polyethyleneglycol (DSPE-PEG), exhibited approximately 30% hydrolysis of the PE component on storage at 4 °C for 68 months.^253^

In a system composed of fully hydrogenated soy PC, investigated as a carrier for carboplatin, 5.4% degradation occurred after 6 months at 4 °C in the dark. Inclusion of cholesterol, ascorbyl palmitate (AP), or both (PC/chol/AP, 70:10:15 by mass), gave, respectively, 0%, 11%, or 12% lysolipid in the same conditions. At room temperature in daylight, the PC/chol system was remarkably stable, but the other systems all exhibited increased lysolipid formation, most notably the systems containing ascorbyl palmitate, where the level of lysolipid was >50%.^254^ In a study on the stability of egg PC liposomes, Samuni et al. also found that the presence of light had little impact on hydrolysis, observing ∼15% and ∼4% formation of FFA in EPC and EPC/chol (10:1) liposomes, respectively, after 16 months at room temperature and pH 7.4.^255^ In this study, the inclusion of vitamin E produced a slight reduction of 1–2% in the extent of hydrolysis.

#### 2.2.3 Membrane Composition and Ionic Strength

In membranes composed of different classes of glycerophospholipids, those that are neutral (PC, PE) tend to exhibit slower rates of hydrolysis than those that are charged (PS, phosphatidylglycerol (PG), PI),^256,257^ with, for example, hydrolysis rates for lipid classes decreasing in rate in the order DPPG > DPPC > 1,2-dipalmitoyl-*sn*-glycero-3-phosphoethanolamine (DPPE) in comparable systems.^219^ The inclusion of charged species into membranes containing a saturated lipid, such as stearylamine, DPPG, cholesteryl sulfate, or dicetylphoshate, generally increases hydrolysis rates,^219,227,244,258^ an effect which has been ascribed to localized changes in the surface pH in accordance with Gouy–Chapman theory.^219,227^ Accordingly, hydrolysis rates in charged membranes are more sensitive to the ionic strength of the medium, with hydrolysis rates increasing at high ionic strength.^259^ The ionic strength of the medium has little effect on the reaction rate in many neutral membranes,^218,219,227^ although liposomes composed of PC and PE have been found to show an increase in size and decrease in retention rate with increasing ionic strength.^260^

#### 2.2.4 The Effects of Membrane Additives

There are some notable deviations from predicted hydrolytic behavior, most notably for amphiphiles such as amines with titratable groups that are ionized at neutral pH. Increased rates in the presence of ascorbyl palmitate^254^ were noted above. Dialkylphosphates have a significant effect on the rate of lipid hydrolysis in a manner that is dependent on the chain length, giving faster rates when the length of the alkyl chain is similar to that of the lipid.^237^ For example, with H-soyPC/dipalmitoyl (10:1) liposomes, 77% of the lipid remained after 28 days (40 °C, pH 7.5), compared with ≥20% for didecyl and dieicosyl phosphate and 96% in the absence of the dialkylphosphate. Interestingly, in this system significant hydrolysis of the lysolipid was also observed. Incorporation of cholesterol increased hydrolysis rates below the *T*_m_ of DPPC and decreased rates above *T*_m_. In both DPPC/DOPE (3:1) and 1,2-dioleoyl-3-trimethylammonium propane (DOTAP)/1,2-dioleoyl-*sn*-glycero-3-phosphoethanolamine (DOPE) (1:1) membranes, the rate of hydrolysis both components is almost pH-independent below pH ∼6.5, which is ascribed to predominant catalysis by the charged ammonium headgroup.^226^

It is increasingly becoming apparent that while many drugs, particularly uncharged compounds such as paclitaxel, have little effect on liposome stability,^261^ others, most notably cationic amphiphilic drugs (CADs), can markedly influence the hydrolytic stability of lipids. Incorporation of gemcitabine (dFdC) into DPPC/DSPC/DPPG_2_ (50:20:30) liposomes produced evidence for increased hydrolysis, with lysolipid levels between 0.6% and 1.3% in approximately a third of the samples following passive loading by incubating the vesicles for 30 min with dFdC at 60 °C and pH 7.4^262^ An earlier report suggests that the nature of the anion can influence the kinetics of hydrolysis promoted by dFdC. In liposomes composed of hydrogenated hen egg phosphatidylcholine (H-EPC)/chol (3:2), higher rates of hydrolysis were found in the presence of more lipophilic anions, decreasing in the order I^–^ > Br^–^ > Cl^–^ > SO_4_^2–^ > F^–^, but only at dFdC concentrations ≥40 mM.^256^ At the highest concentration of dFdC (80 mM), in the presence of 50 mM NaI, 20% lysolipid formation was noted after 66 h at 60 °C, compared with <2.5% in the absence of dFdC. The precise role of the anion is uncertain, but it likely that it provides a counterion to the dFdC ammomium ion to facilitate partitioning of dFdC into the membrane.

More recent studies of the effects of CADs have identified a lipid dependence on the rate of hydrolysis. Casey et al. found that 1,2-dioleoyl-*sn*-glycero-3-phosphocholine (DOPC) liposomes incorporating 5 mol % raclopride were hydrolyzed at a rate of 0.1136 mol h^–1^ mol_RAC_^–1^ over a 22 day period, producing 3.3 mol % at the end of the experiment. DOPC hydrolysis in the absence of raclopride was minimal over the same period.^263^ Hydrolysis in saturated lipid systems, or mixtures of DOPC with a high saturated lipid content, was significantly faster, with the rate increasing with at longer saturated chain lengths. A strong correlation was found between the rate of hydrolysis and *T*_m_. Raclopride was proposed to act as a phase transfer catalyst, effectively promoting hydrolysis by acting as a general acid catalyst. Earlier research from the same group reached similar conclusions for the activity of haloperidol and spiperone in DOPC membranes.^264^ A total of 12 CADs was studied, and although the levels of activity varied, promotion of hydrolysis was found to be a general property of CADs. CADs will protonate at physiological pH, in turn initiating acid-catalyzed ester hydrolysis of the membrane. The rate will be determined by the mechanical state of the membrane (such as curvature elastic stress) as well as the chemistry of the CADs. Membranes made of 1,2-dilinoleoyl-*sn*-glycero-3-phosphocholine (DLPC; lower stored curvature stress) will hydrolyze at a slower rate than DOPC systems.

#### 2.2.5 Physicochemical and Biological Effects of Hydrolysis

Hydrolysis of a single acyl chain yields equimolar FFA and lysolipid. Subsequent hydrolysis of the second acyl chain from the lysolipid, forming water-soluble GPC and another equivalent of FFA, will shift the balance of lysis products in favor of FFA. Studies show that on their own, both lysolipids and FFA increase disorder in the membrane, leading, for example, to the decreased retention of encapsulated drugs. However, the presence of both lysolipids (lyso-phopshatidylcholine, LPC) and FFA increases the stability of the bilayer, making the bilayer even less permeable than with phospholipids alone.^203,227,265^

In some systems, significant changes in morphology accompany lipid hydrolysis. In DPPC/DSPE-PEG2000 liposomes, lysis resulted in the formation of bilayer sheets and discs, but only upon heating and then recooling through the main gel to liquid crystalline phase transition, indicating that the initial mixture containing lysolipids and FFA is metastable.^229^ Strikingly, this effect was produced with <10% DPPC hydrolysis when the sample was stored at pH 4 and <5% when stored at pH 2. Disc formation was attributed to stabilization by lysolipids partitioning to the highly curved areas at the edge of the disc, an effect that has been noted elsewhere.^266^

The ability of hydrolysis reactions to produce morphological changes has recently been exploited by Kodama et al.^267^ They used the localized microinjection of hydroxide ions to the internal surface of a giant unilamellar vesicle (0.1 mol % rhodamine-labeled DOPE in DOPC) to drive lipid hydrolysis and generate movement of the vesicle. Related effects have been observed for the formation of complex 3D topologies from supported 1-palmitoyl-2-oleoyl-*sn*-glycero-3-phosphocholine (POPC) bilayers, including the formation of protrusions from the bilayer surface, following treatment with hydroxide ions at pH ≥11 or acidic conditions at pH ≤1.^268^ In this case, the effects were not directly attributed to hydrolysis, as protrusions formed significantly faster than the predicted rate of hydrolysis, although the involvement of some lysolipid in stabilizing highly curved structures might still contribute to the observed changes.

The presence of lysolipids increases bilayer permeability and significantly disrupts bilayer structure and morphology at relatively low levels (<10 mol %). In most healthy cells the levels of lysolipids are regulated at <6% of total membrane lipids. Elevated levels of lysolipids are associated with diseases such as cancer and cardiovascular disease. Lysolipids have other roles in cell physiology, including signaling, reproduction and the inflammatory response.^203,269^

### 2.3 Hydrolysis of Plasmalogens

**Scheme 2:**
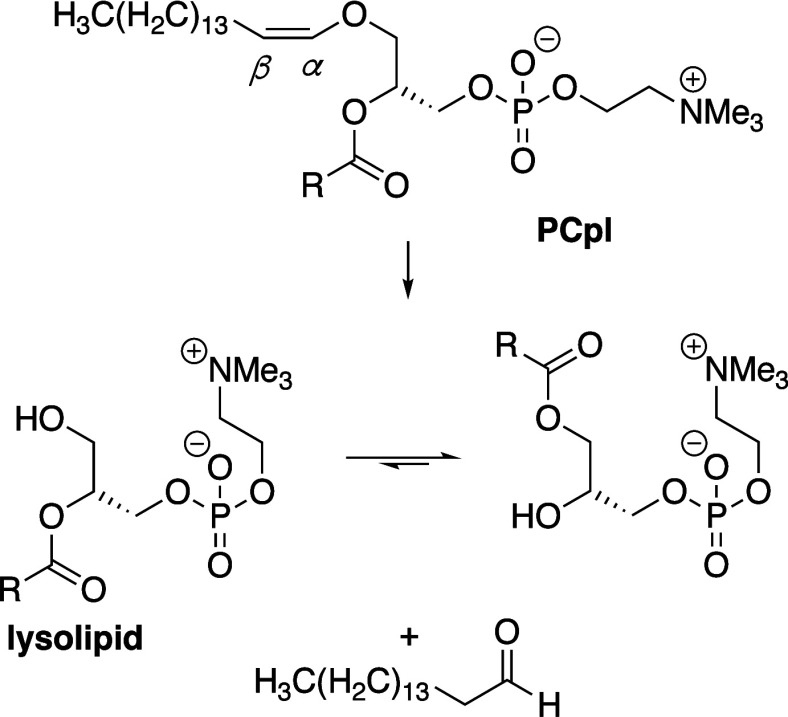
Hydrolysis of Plasmenyl Lipids

Vinyl ether linked lipids, such as plasmenylcholines, are found in many eukaryotic membranes and are generally more susceptible to hydrolysis than glycerophospholipids. This susceptibility arises from the electron-rich nature of the enol ether and results in preferential hydrolysis of this group over the adjacent ester (Scheme 2).^242^ First-order rate constants for hydrolysis of plasmenylcholines are 2–3× higher than those of glycerophospholipids under comparable conditions (Table 1).

The reactivity of vinyl ether lipids has been exploited for the development of drug delivery systems. Liposomes of a bis-vinyl ether lipid, 1,2-di-*O*-((*Z*)-1′-hexadecenyl)-*sn*-glycero-3-phosphocholine (DPPlsC), incorporating calcein, did not exhibit significant leakage at 37 °C and pH 7.4 over a 48 h period. At pH 4.5, significant calcein release was observed after a period of one hour, at which point the lipid was >20% hydrolyzed.^243^ This approach was used to successfully demonstrate targeted delivery of the drug Ara-C to KB cells using liposomes composed of DPPlsC /7-dehydrocholesterol (DHC)/DSPE-PEG_3350_-folate (9:1:0.05). Controlled release using liposomes containing vinyl ether groups has been reviewed.^270^

### 2.4 Other Hydrolyses

Few reports exist of other types of hydrolytic reactivity of lipids. Poznik et al. recently reported that lanthanide ions can act as Lewis acid catalysts in the decomposition of phosphodiesters.^271^ Bis-4-nitrophenyl phosphate (BNPP) was used to model the activity of La(III), Ce(III), Eu(III), Tb(III), and Yb(III) complexes in the presence of DOPC membranes. Although significant catalytic activity was found for complexes of these ions, no direct hydrolysis of lipid phosphates was reported. One report exists of a PC demethylation to form PE.^254^ This demethylation was reported for liposomes composed of H-soyPC and H-soyPC/chol containing ascorbyl palmitate (in a 7:1:1.5 ratio) that encapsulated carboplatin, where PE accounted for 5% of the material after 6 months at pH 3.5–4, regardless of temperature (4 °C/room temperature). Partially demethylated PC and lyso-PE were not detected.

### 2.5 Future Directions

While the fundamental aspects of membrane lipid hydrolysis are well established, there remain areas where further research is needed, most notably in better understanding the relationship between oxidation and hydrolysis and in predicting the stability of complex formulations containing lipid membranes alongside excipients and other active molecules. With regard to the latter, while there have been some studies that have found that lipid hydrolysis can be promoted by drugs and small organic molecules,^263,264,272^ others have found evidence to suggest that some compounds actually reduce the rate of background hydrolysis.^273^ The effects of drugs and related molecules on lipid stability remain underexplored.

## 3 Aminolysis and Transesterification

### 3.1 Intrinsic Lipidation

**Scheme 3:**
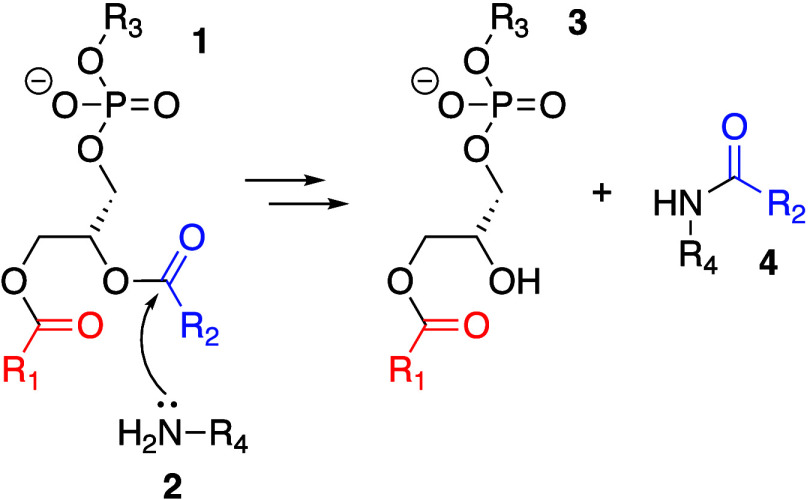
Aminolysis Reactions of Glycerophospholipids

Direct reaction between membrane-embedded molecules and membrane lipids, termed “intrinsic lipidation”, has been little studied until relatively recently. As hydrolysis reactions at high pH involve nucleophilic attack of hydroxide ions on the ester carbonyl group, it should not be surprising that other nucleophilic groups within the membrane are capable of participating in a similar attack to form a lysolipid (**3**, Scheme 3) and a lipidated product, an amide in the case of an amine (**4**) or an ester should the nucleophilic group be an alcohol.

**Figure 3:**
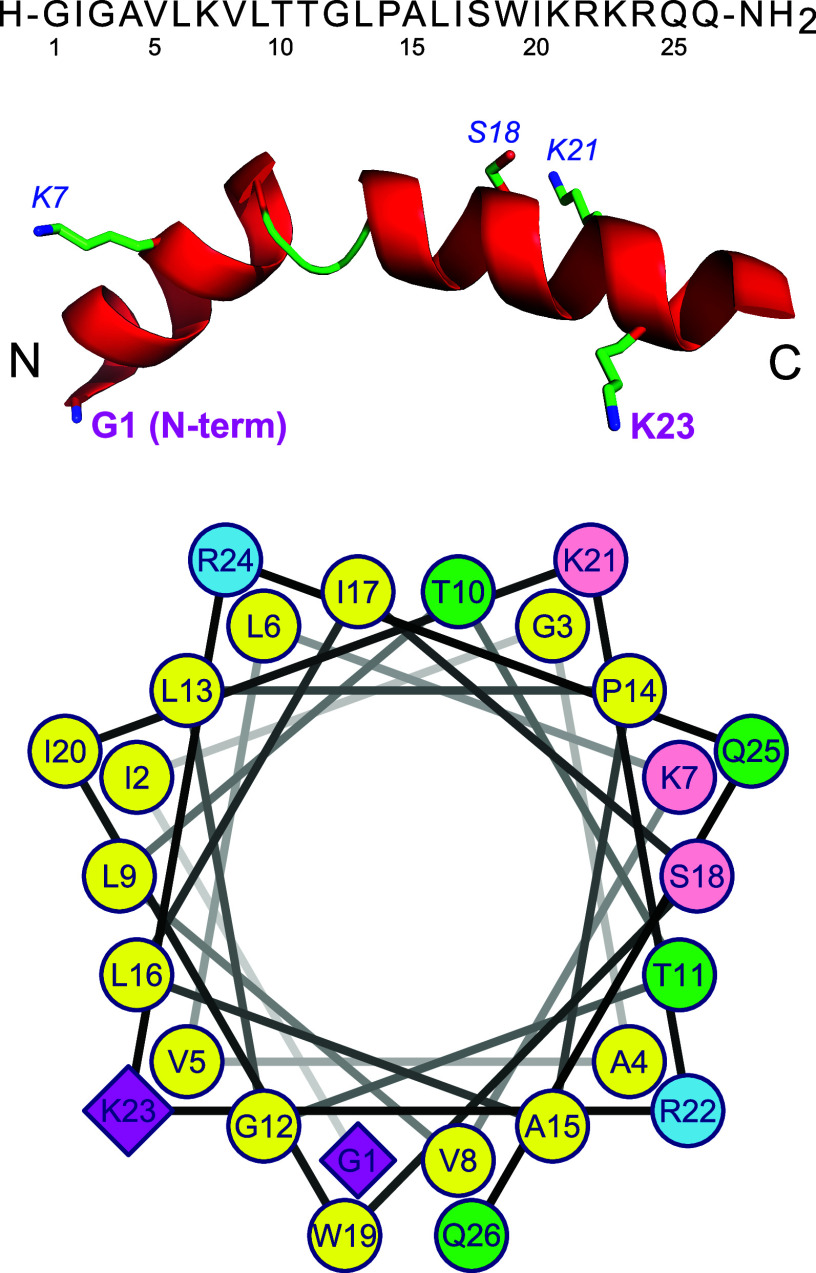
Sequence and structure of melittin: top, sequence; middle, crystal structure (PDB code 2MLT)^277^ with the major acylation sites shown in bold and the minor sites in italics; and bottom, helical wheel representation with the major acylation sites shown as diamonds, the minor sites shown as pink, charged residues shown as blue, polar uncharged residues shown as green, and apolar residues shown as yellow.

Membrane-embedded peptides have been shown to undergo acyl transfer reactions by direct reaction with lipids under physiological conditions (pH 7.4, 37 °C).^274−276^ These reactions, with a typical *t*_1/2_ of 20–40 h, are faster than hydrolysis reactions at this pH. The prototype for this reactivity is melittin. This 26-residue peptide (Figure 3) undergoes aminolysis reactions with diacylglycerophospholipids that involve the amino groups of internal lysine residues and the N-terminal amino group, and transesterification reactions involving an internal serine.^275^

**Figure 4:**
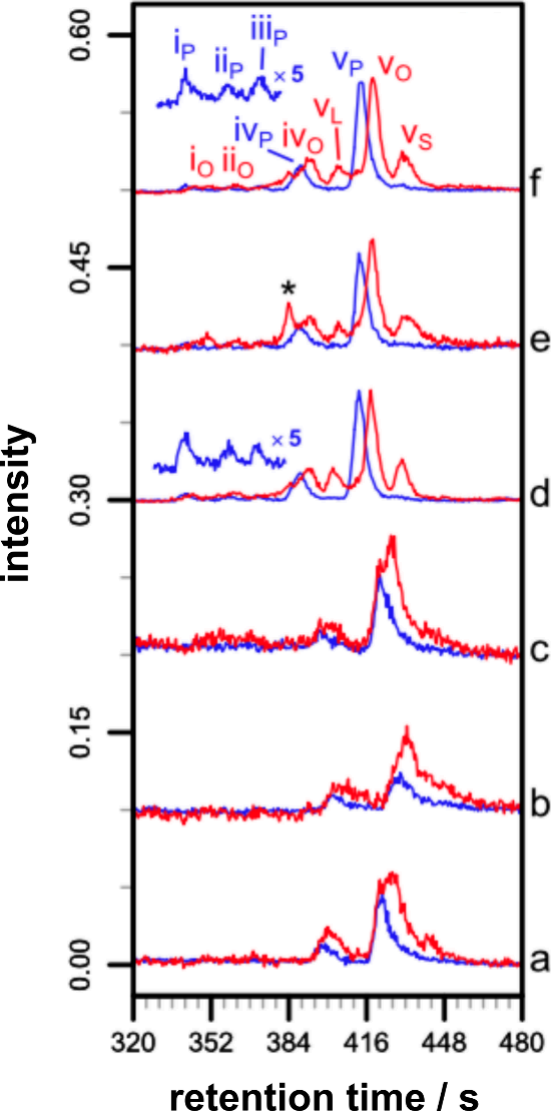
LC-MS analysis of melittin/liposome mixtures. Reaction conditions unless otherwise stated: [melittin], 26 μM; [POPC], 0.26 mM (P/L, 1:10), [NaCl], 90 mM; NaHCO_3_, 10 mM; pH 7.4; 37 °C. Key: rt, retention time. (a) No NaHCO_3_, 72 h. (b) 20 °C, 150 mM NaCl, 72 h. (c) Water (no NaHCO_3_ or NaCl), 48 h. (d) POPC/SLPS (4:1), 72 h. (e) POPC/SLPG (4:1), 48 h. (f) POPC/SLPE (4:1), 72 h. (a) Total ion chromatograms (TICs; area normalized). The broken box indicates the region in (b). (b) Extracted ion chromatograms (EICs; area normalized) for palmitoyl–melittin (blue) and oleoyl–melittin (red). For (d–f), combined EICs for oleoyl/stearoyl/linoleoyl–melittin are shown in red. The peak indicated by an asterisk is from a polymeric impurity. Chromatographic peak identities are annotated using a roman numeral to indicate the main site of peptide modification responsible for the peak, with a subscript to identify the acyl group; i–v correspond to S18, K21, K7, K23, and the N-terminus, respectively. Reprinted with permission from ref (274). Copyright 2013 Elsevier.

As with hydrolysis reactions, no selectivity is found for reaction at the acyl groups of *sn*-1 and *sn*-2 positions of the glycerol backbone, but selectivity is seen for the site of reaction on the peptide. The reactivity of the available nucleophilic centers on the peptide can be ranked in the order N-terminus > K23 ≫ K21 ≈ K7 > S18 (Figure 4).^275^ This ranking of reactivity is likely to be a consequence of a number of factors, including the p*K*_a_ of the ammonium form of the amine and positioning of the reactive group within the membrane. The p*K*_a_ of the N-terminal ammonium group of melittin is in the range 7.15–8.15 in a micelle-associated form, which is significantly lower than that of the internal lysine residues (9.2–10.2 for K21, K23, and K7)^278,279^ and consistent with the higher reactivity of the N-terminal amine, significant amounts of which will be in the neutral amine form within the membrane interface at physiological pH. The difference in reactivity between K23 and K7/K21, which cannot be accounted for simply in terms of p*K*_a_, is likely to reflect their different positioning within the membrane-embedded state of the peptide, with K23 being positioned close to the bilayer interface. Indeed, for other amphiphilic peptides, including magainin II and PGLa, reactivity is most frequently observed for residues that are located close to the interface between the polar and hydrophobic surfaces, which would position these residues in the interfacial regions of the bilayer.^274^ Peptides without interfacial reactive groups, such as penetratin, only undergo the reaction in very specific circumstances, such as high salt concentrations. Additional groups in melittin also undergo reaction with lipids, most notably the side chain hydroxyl of S18 to form ester-linked lipidated peptides, but the reactivity of these groups is lower than amines.^275^ Other products resulting from reactivity between melittin and membrane lipids have been detected, but they are extremely prone to in-source fragmentation during MS analysis and it has therefore been difficult to establish their identity. Potential sites for these reactions include the side chain of a histidine residue. In membrane models comprising two lipid classes with similar acyl chain compositions, such as POPC/1-stearoyl-2-linoleoyl-*sn*-glycero-3-phosphoethanolamine (SLPE), POPC/1-stearoyl-2-linoleoyl-*sn*-glycero-3-phosphoglycerol (SLPG), and POPC/1-stearoyl-2-stearoyl-*sn*-glycero-3-phosphoserine (SLPS) (4:1 in all cases), in which acyl transfer from the *sn*-1 and *sn*-2 positions of each lipid class can be distinguished, acyl transfer is seen from both the lipid classes in accordance with their relative abundance, with no appreciable selectivity between reactivity at the *sn*-1 and *sn*-2 positions (Figure 4). Differences are found in the overall reactivity of these systems, however, with POPC/SLPE being the most reactive, suggesting a role for the biophysical properties of the membrane in determining reactivity. Consistent with this, for lipid mixtures where the acyl chains are less well matched, differing reactivity patterns are seen, with transfer only found from DOPC in DOPC/1,2-distearoyl-*sn*-glycero-3-phosphoserine (DPPS) mixtures, but both lipid classes in DOPC/1,2-dimyristoyl-*sn*-glycero-3-phosphoglycerol (DMPG) mixtures.^275^

Other factors that could influence reactivity have been addressed, including temperature, the ionic strength of the medium, the role of the buffer, the counterion of the peptide, and the peptide/lipid ratio (in the range 1:10 to 1:100).^274^ Generally, both higher temperatures and increased salt concentrations favored the reaction, but the effects of other parameters were found to be small. It has also been demonstrated that peptides are able to undergo similar processes with lysolipids.^280^ A consequence of peptide lipidation in this manner is expected to be an increase in the membrane affinity of the lipidated peptide. This will be reflected by irreversible membrane binding. Examples exist in the literature where peptide association with membranes is not completely reversible, including association of the designed peptide TMX-1 to POPG membranes^281^ and melittin to PEG-stabilized POPC/palmitoyl-2-oleoyl-*sn*-glycero-3-phosphoglycerol (POPG) nanodiscs.^266^ Although analytical speciation of these mixtures was not conducted at the end of these experiments to determine whether lipidation had occurred, it remains a strong possibility that this is the prime reason for irreversible binding in cases such as this.

Interestingly, the inclusion of cholesterol into PC membranes significantly increases reactivity, with in some cases up to 50% of melittin converted into lipidated products within 24 h.^282^ This higher reactivity occurs in spite of the reduced affinity of melittin for membranes containing cholesterol and is attributed to the change in disposition of the peptide in cholesterol containing membrane. Consistent with this change in disposition, the pattern of reactivity also shifts, with lipidation at K23 becoming the predominant product. In these conditions it is implicit that the rate-determining step is not associated with initial nucleophilic attack of reactive groups on melittin and as a consequence the membrane affinity is not a significant factor in predicting the rate of lipidation.

**Figure 5:**
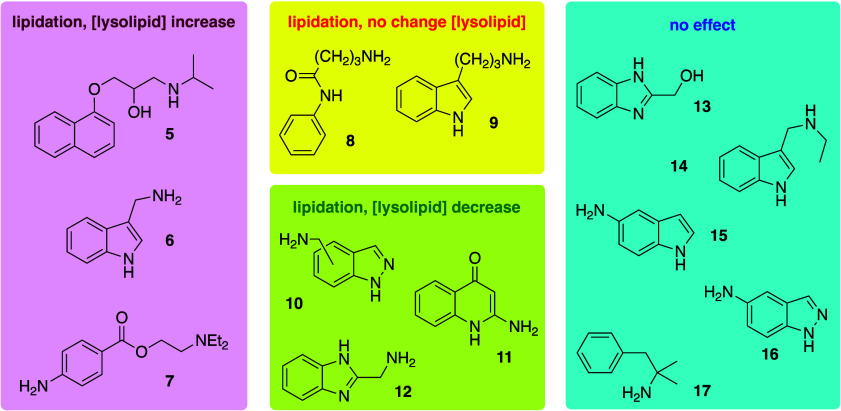
Lipidation activity of exemplar low molecular weight organic molecules with neutral phospholipid membranes.^272,273^ The changes in lysolipid concentration are ≥24 h following the addition of the compounds to liposomes. Compounds with “no effect” yielded neither lipidated products nor changes in lysolipid levels relative to controls.

This nondependence of reactivity on membrane affinity is seen most strikingly for small organic molecules, many of which have low predicted membrane affinity based on their log *P* values and exhibit a spectrum of behavior from no effect on membrane stability, through effects on the rate of lipid hydrolysis, to participation in lipidation reactions (Figure 5).^273^ Notable examples are the drug propranolol (**5**), for which lipidation on the alcohol occurs almost exclusively rather than at the secondary amino group, both in model systems *in vitro* and HepG2 cells.^272^ Propranolol lipidation is likely driven by a combination of membrane partitioning of the predominant ammonium form (at physiological pH) to an appropriate depth to enable reaction on the oxygen, combined with disfavored reaction of the minor neutral form at the nitrogen center for steric reasons. Lipidation is generally disfavored by steric bulk near the reactive center, exemplified by the difference in reactivity of 3-aminomethylindole (**6** and the corresponding *N*-ethyl analogue (**14**). Exchange of the nucleophilic group from an amino group to an alcohol can be sufficient to eliminate reactivity (e.g., **12** vs **13**). Furthermore, many of the amino analogues of reactive aminoalkyl -substituted aromatics, exemplified by **16** (for 5-aminomethylindazole, **10**), exhibit no apparent reactivity in membranes despite having lower p*K*_a_ values than their aminoalkyl counterparts, which would be expected to favor the partitioning of the neutral form into the membrane. These examples further illustrate that the disposition of molecules in the membrane is likely to be of greater importance for reactivity than the overall affinity of a molecule for the membrane.

As lysolipids, whether formed by background hydrolysis or as byproducts of aminolysis or transesterification reactions, are potential sources of acyl groups for lipidation reactions, their concentration profiles can fluctuate during the progress of an intrinsic lipidation reaction. To add to this complexity, some compounds may be able to simultaneously participate in aminolysis or transesterification reactions and promote lipid hydrolysis. Indeed, after periods ≥24 h following compound addition to liposomes, compounds such as propranolol yield an increase in lysolipid concentration. Other compounds, however, most notably the aminomethylindazole family (**10**), yield decreased lysolipid concentrations, presumably reflecting either a greater reactivity with lysolipids than lipids or an activity to reduce the rate of background hydrolysis. It is noteworthy that some compounds (e.g., **8**, **9**) have no net effect on lysolipid concentrations, and others are able to increase the rate of background hydrolysis without any evidence of lipidation. In membranes with a negative charge, broadly similar patterns of intrinsic lipidation activity are found, with some variation in the nature of lysolipid changes observed for some compounds.

In principle, membrane proteins should undergo similar reactions *in vivo*. In many cases, however, the protein turnover rate will prevent the products from these reactions from accumulating. Protein degradation half-lives in mammals typically range from <1 h,^283,284^ through 2–4 days in the liver and blood, to >10 days in the brain.^285,286^ A small number of proteins have extremely low or zero degradation rates, including structural proteins such as collagen and proteins in postmitotic cells such as the nucleus of the lens, in which the normal cellular machinery is absent and as a consequence proteins are not recycled.^283^ It is reasonable, therefore, to expect the products of the intrinsic lipidation of membrane proteins to accrue in scenarios where the normal cellular machinery is absent, such as in extracellular fluids and postmitotic cells. In cases where intrinsic lipidation may occur *in vivo*, the observations from peptide lipidation in model membranes enable a prediction that it will result in incomplete lipidation at unusual residues that reside close to the membrane interface but do not conform to a consensus sequence, yielding an acyl group distribution that reflects the acyl composition of the host membrane.

Lung surfactant protein C (SP-C) is initially synthesized as a precursor protein, proSP-C. Palmitoylation of proSP-C occurs at two internal cysteine residues with high efficiency^287,288^ under enzymatic control, occurring between the ER and the *cis*-Golgi.^289,290^ After processing to SP-C, the protein is incorporated into lamellar bodies (multilamellar vesicles) and transported to the plasma membrane, where fusion of the lamellar bodies releases SP-C into the liquid layer lining the airway epithelium. Examination of SP-C obtained from bronchoalveolar lavage has identified a third SP-C lipidation site. This site is palmitoylated at about 4% and has been identified as modification of an internal lysine, K11, by tandem mass spectrometry approaches.^291,292^ Lung surfactant is 78–90% phospholipid, with 50–70% of this being DPPC. The site of SP-C modification, the identification of the acyl group as palmitoyl and the incomplete conversion are all consistent with an intrinsic lipidation mechanism. Furthermore, the modified lysine is predicted to be close to the membrane interface in the transmembrane helical form of the protein and upon storage the extent of lysine modification was found to increase.^292^

Aquaporin-0 (AQP0, also termed the lens major intrinsic protein) is a membrane protein that, in fiber cells of the eye lens, has roles in water transport and the formation of gap junctions. Lens fiber cells are arranged concentrically, from the youngest cells in the outer cortex to the oldest (embryonic) cells in the lens nucleus.^293^ New fiber cells are added to the outer cortex from the epithelium that surrounds it, but during lens fiber cell differentiation cell organelles are removed and normal recycling of cellular components stops.^294^ As a consequence, fiber cells are postmitotic and contain some of the oldest proteins in the body. Post-translational modifications (PTMs) to AQP0 have been studied extensively by Schey and co-workers.^295−302^ They have documented a range of PTMs, including truncation, oxidation, deamidation, phosphorylation, and lipidation. The lipidation of AQP0 is unusual, occurring at the N-terminus and an internal lysine, K238, with the predominant modifications being oleoyl and palmitoyl in decreasing order of abundance.^298^ Recently, it has become apparent that the lipidation profile at both these sites includes a range of acyl modifications. For bovine AQP0, the acyl modifications at the N-terminus include, in decreasing order of magnitude, C18:1 ≫ C16:0 > C18:0 > C20:1 > C20:3 ≈ C16:1 > C20:2.^303^ Many of these modifications are also found at K238, with a similar relative abundance. Human AQP0 similarly has an extensive lipidation profile that differs by the inclusion of two different C20:4 and C20:3 modifications, as well as C22:4 and C20:5, although the latter two were only detected as oxidized species. For both human and bovine AQP0, but particularly the latter, there is a striking correlation between the abundance of the acyl modifications and the population of ester-linked acyl groups within the PE class of lipids (including plasmalogens). It is noteworthy that PE lipids are a major component of the cytoplasmic membrane leaflet to which the N-terminal amino group and K238 are proximal.^20^ Modification by intrinsic lipidation is consequently a distinct possibility, although lipidation by direct acyl transfer from membrane-associated Acyl-CoA has also been shown to be a viable process^304−308^ and cannot be completely ruled out as AQP0 passes through the ER, a major site of phospholipid biosynthesis.^20^ AQP0 lipidation is first detectable at about 20–30% of the distance from the outer cortex to the center of the lens nucleus and increases up to 60% of that distance before reaching a plateau.^295,297^ The value at this plateau increases with age from ∼30% in an 11 year old to ∼50% in a 32 year old.^297^ The increases in AQP0 lipidation both with age and proximity to the lens nucleus argue in favor of lipidation from the membrane.

Recently there has been a surge of interest in post-translational modifications to lysine. A range of modifications are known, many involving metabolic intermediates and most involving the formation of amides involving the lysine amino group.^309^ Concomitant with this has been increased interest in the sirtuins, a family of NAD^+^-dependent deacylase enzymes that catalyze the removal of these PTMs. Seven sirtuins are present in humans (SirT1–7), but they are present at varying levels in almost all organisms.^310^ The discovery that some sirtuins, including mammalian SirT6^311^ and Sir2A from *Plasmodium falciparum*(312) selectively hydrolyze long-chain acyl groups from the side chains of internal lysine residues suggests that this reaction is important enough *in vivo* for the evolution of protective mechanisms.^313^

Overall it is clear that molecules of any size that are able to interact with membranes have the potential to undergo direct acyl transfer reactions with the lipid to become lipidated, provided that there is a nucleophile suitably disposed in at least one bound configuration. This should be of some concern in instances where lipids are used to formulate potentially reactive drugs, such as in liposomes or solid lipid nanoparticles. Should protective mechanisms have evolved to recycle lipidated proteins or remove acyl modifications, the intrinsic lipidation process should also be of concern during the storage of foodstuffs. In biological systems, intrinsic lipidation has been proposed as one route by which amyloid formation could be triggered.^101,314^

### 3.2 Future Directions

The recognition that some molecules embedded in the membrane can be lipidated by direct acyl transfer from the lipid occurred relatively recently. There are still gaps in our understanding of this process with regard to both the molecular features and membrane dispositions that promote lipidation and the consequences of lipidation *in vivo* for the activities of biomolecules, as well as the distribution and clearance of drugs. It is likely that lipidation occurs in tissues or organelles where proteins have a slow turnover, but this is challenging to prove. There may also be cellular corrective mechanisms that are as yet unrecognized. In this regard, sirtuins with broad substrate specificity have been proposed as enzymes that may carry out this role.^101^ In some diseases, notably those involving amyloid formation, many of the long-lived deposited materials contain lipids alongside proteins, and it has been proposed that amyloid peptide lipidation may play a role in the nucleation process; however, this has yet to be proved.^314^ It will be interesting to examine whether lipidated peptides with diverse fatty acid profiles can be identified in proteomics studies. Currently, in many proteomics studies using LC-MS, the usable gradient stops short of mobile phase compositions where lipidated peptides would elute and be fragmented to permit identification.

## 4 Oxidation

The chemistry of lipid oxidation is very well documented due to its importance in food chemistry, liposome stability, and biological redox processes. By far the most important reactivity involves the alkene groups of unsaturated fatty acids, which are particularly liable to oxidation by atmospheric oxygen (autoxidation). Alkene reactivity is also a feature of the oxidative reactions undergone by plasmalogens, sphingolipids, and sterols. Oxidative processes involving free radical intermediates are known for other functional groups, particularly when reactive alkenes are not present, but these reactions only tend to occur following the formation of reactive intermediates during processes such radiative damage.

### 4.1 Free Radical Oxidation of Glycerophospholipids

**Scheme 4:**
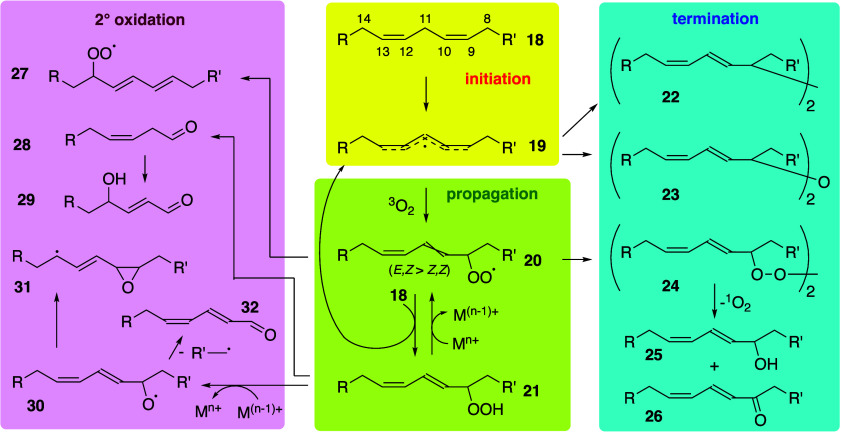
Overview of Processes Involved in the Autoxidation of Homoconjugated Dienes The atom numbering of **18** corresponds to the carbon atom numbers of linoleic acid.

Oxidative reactions of fatty acids have been well reviewed by Spickett, Pratt, and Porter,^74,315−317^ plus a number of other recent reviews.^108,112,181,318−326^ An overview of the main processes is given in Scheme 4.

#### 4.1.1 Initiation

Unsaturated fatty acids are liable to oxidation by the initial formation of a bis-allylic radical by hydrogen abstraction. Allylic radical formation is particularly prevalent in naturally occurring polyunsaturated fatty acids (PUFAs) as the double bonds are homoconjugate, being separated by a single methylene group. For example, *cis*,*cis*-Δ^9^,Δ^12^-octadecadienoic acid (lineoleic acid), commonly used as a model for oxidation reactions, has double bonds between carbon atoms 9/10 and 12/13 of the acyl chain, separated by a single methylene at carbon 11. Respective C–H homolytic bond dissociation energies for allyl and bis-allyl hydrogens are 65 and 77 kcal mol^–1^.^181^ Rate constants (*k*_i_) for initiation (typically 10^–6^ s^–1^)^327^ are significantly lower than those for propagation or termination. For studies of liposome aging *in vitro* and oxidative processes in food, initiation is frequently enhanced by the addition of free radical initiators to increase the overall initiation rate (*k*_i_[In^•^]). These have been well reviewed.^317,326,328^ Commonly used examples include water-soluble agents such as 2,2′-azobis(2-methylpropionamidine)dihydrochloride (AAPH),^329^ and lipophilic agents such as di-*tert*-butylhyponitrite (DBHN),^330^*t*-butylhydroperoxide,^139^ and 2,2′-azobis(4-methoxy-2,4-dimethylvaleronitrile) (MeO-AMVN).^331^

Photoinitiators react under light irradiation (UV or visible) to generate intermediates capable of initiation. Caution is needed when referring to photoinitiator types, with differing conventions sometimes used according to the circumstances of the process. For reactions involving oxygen, type I photoinitiators produce reactive radical intermediates that combine with oxygen or the superoxide anion radical (O_2_^•–^) to form oxygenated products. Type II photoinitiators induce energy transfer to oxygen to form singlet oxygen.^332−334^ In the absence of oxygen, photoinitiators that form radical intermediates capable of direct initiation are referred to as type I, and those that are able to extract hydrogen from a co-initiator are referred to as type II.^335,336^ Further caution is needed as some lipophilic photoinitiatiors may be mixed mode, which while being type I in principle give product distributions more typical of a type II photoinitiator within the hydrophobic core of the membrane.^337^

In the absence of organic free radical initiators, the most common sources of *in vitro* initiation are high valence metal ions,^338^ hydroxyl radicals generated by the action of redox-active metal ions on hydrogen peroxide (such as the Fenton reaction), and reactive oxygen species (ROS) generated photochemically,^339^ or by ionizing radiation.^74^ Irradiation of O_2_ by light of wavelength <240 nm generates a range of ROS, including singlet oxygen (O(_1_D)), hydroxyl radicals, ozone, and hydrogen peroxide.^340^

In some cases, agents that are able to mediate the generation of lower valence metal ions and act as hydrogen atom acceptors accelerate the initiation process. For example, ascorbate significantly enhances the oxidation of methyl linoleate by Fe(III).^341^ The proposed mechanism for this enhancement involves the formation of a trinuclear Fe(III) cluster with ascorbate that undergoes internal electron transfer to form an Fe(II) ascorbyl radical pair. The ascorbyl radical in this complex abstracts an allylic hydrogen from the fatty acid to release Fe(II) and ascorbic acid (p*K*_a_ 4.8), which deprotonates to regenerate ascorbate.^341,342^ This process is therefore cyclic with respect to ascorbate and gives optimal initiation rates with 3–5 equiv of ascorbate per Fe(III) due to the stoichiometry of the active clusters. The Fe(II) released is then available to participate in peroxide decomposition reactions to form peroxyl or hydroxyl radicals and reform Fe(III).^343^ At higher levels, ascorbate reverts to acting as an antioxidant through radical scavenging. This difference in activity, being pro-oxidant at low levels and antioxidant at high levels, is one example of the complex behaviors that many antioxidants exhibit in different concentration ranges or in the presence of metals.^344^ The PC demethylation over a period of 6 months in H-soyPC and H-soyPC/chol (7:1) liposomes encapsulating carboplatin and incorporating ascorbyl palmitate (AP),^254^ discussed earlier in this Review, is potentially accounted for by formation of reactive ascorbyl intermediates in a similar manner to the Fe(III)/ascorbate oxidation. The reduction of Pt(IV) to Pt(II) by ascorbate is a known process,^345^ although being a net 2 electron transformation, the generation of an alkyl radical would necessitate the intermediacy of a Pt(III) species, as well as the generation of Pt(IV). Pt(III) intermediates, alongside ascorbyl radical formation, have been proposed during ascorbate reduction of Pt(IV),^346^ and in some cases the electron paramagnetic resonance (EPR) spectrum of the ascorbyl radical is observable during the reduction.^347^ The formation of dihydroxo Pt(IV) species from hydrogen peroxide is well-known^348−350^ and has been demonstrated for carboplatin.^351^ Hydrogen peroxide formation could occur in the early stages of autoxidation to facilitate Pt(II) oxidation. Oxidation to Pt(III) can also be mediated by the hydroxyl radical.^352^ The C–H bond dissociation enthalpy of the choline methyl is of the order of 100 kcal mol^–1^,^353^ so it is unlikely that the ascorbyl radical will be sufficient to form a methyl radical. However, crystal structures of ascorbate complexes with Pt reveal the presence of very strong Pt–C bonds that could provide sufficient energy to effect hydrogen abstraction.^354,355^

Inorganic phosphorus and sulfur radicals are not generally well studied as initiators, but some are sufficiently hydrophobic that their reactivity with lipids could be significant. Both the S-centered bisulfite radical and the P-centered dihydrophosphite radical should prefer to undergo addition reactions with lipids,^356^ as has been proposed for carbonate radicals.^357^

In biological systems, the principal ROS involved in hydroxyl radical formation are superoxide and hydrogen peroxide, formed by mitochondria as a consequence of electron transfer to oxygen during oxidative phosphorylation.^114,358^ A good review of atmospheric ROS and reactive nitrogen (RNS) generation is given by Pöschl and Shiraiwa.^359^ Hydrogen peroxide can also be generated by the oxidation of superoxide by redox-active metal ions (the first half of the Haber–Weiss reaction). In terms of oxidation, hydrogen peroxide itself is relatively inert toward lipids, but photochemical or metal-induced dissociation of hydrogen peroxide generates hydroxyl radicals. Peroxynitrite (ONO_2_^–^) exists in equilibrium with its conjugate acid (p*K*_a_ 6.8) and is formed *in vivo* from nitric oxide (NO^•^) and superoxide. The acid form decomposes thermally and under metal catalysis to form, among other species, hydroxyl radicals and NO_2_^•^. The anionic form can combine with carbon dioxide to form an adduct that can decompose to form NO_2_^•^ and the carbonate radical. The reaction of superoxide with hypochlorous acid is an additional method for hydroxyl radical generation *in vivo*, although this method may only occur in a few special cases.^81,360^

The production of ROS (and RNS^334^) *in vivo* by ionizing radiation leads to the activation of cellular mechanisms to regulate oxidative stress that can amplify the response in relation to the actual level of ROS generated.^360^ Although the ability of ionizing radiation at environmental levels to produce oxidative stress *in vivo* has been questioned,^361^ the production of ROS at higher radiation doses, such as those used in medical and biotechnology applications, is still potentially significant. Irradiation of deoxygenated water leads to the formation of a solvated electron, the water cation (H_2_O^+^), and electronically excited water (H_2_O*). The water cation combines with water to from a hydroxyl radical and a hydronium ion (H_3_O^+^). Electronically excited water decomposes to H^•^ and OH^•^. In oxygenated solutions, the free electron can additionally reduce oxygen to form superoxide.^362^

#### 4.1.2 Propagation

**Figure 6:**
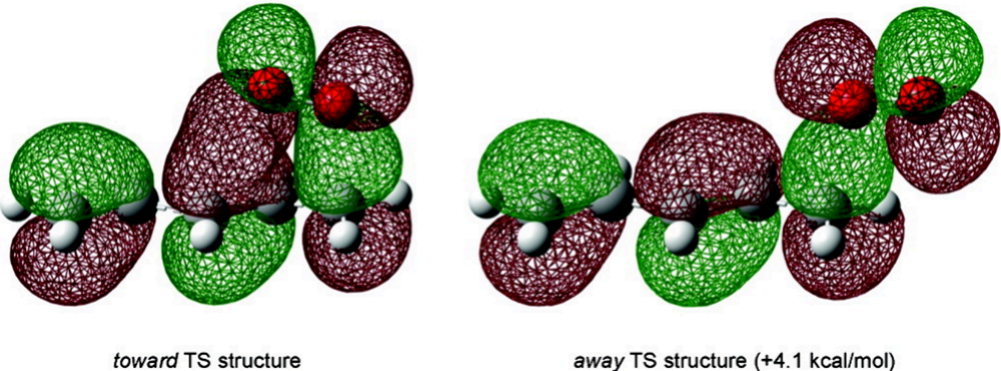
Secondary orbital interactions lead to a preferred geometry in the transition state structure for the reaction between pentadienyl radicals and molecular oxygen. From ref (368). Copyright 2011 American Chemical Society.

Reaction of the allylic radical **19** with triplet oxygen forms a peroxyl radical (**20**). Abstraction of a hydrogen atom by the peroxyl radical from the diene (**18**) forms a hydroperoxide (**21**), plus a further equivalent of **19**, and is usually the rate limiting step in autoxidation.^316,363^ Evidence for this step being rate-limiting in membranes arises from observations that deuteration of the allylic positions of the PUFAs in cell models significantly reduces the overall rates of peroxidation in response to oxidative challenge by Fe(III),^364^ Cu(II),^365^ and hydroxyl radicals generated from Cu(II)/ascorbate.^366^ The magnitude of the kinetic isotope effects in these examples also indicate that tunneling is involved in the hydrogen transfer process.^316^ In the presence of ions such as Fe^3+^, hydroperoxide **21** can be converted back to the peroxyl radical **20**, although this is a slow process.^341^ Hydroperoxide formation in colloidal dispersions favors the formation of the (*E*,*Z*)-configuration in **21**; in homogeneous solutions, formation of the (*Z*,*Z*)-configuration predominates.^367^ The carbon-centerd bis-allylic radical that initially forms is resonance stabilized and can react in principle at any of three carbon centers (carbons 9, 11, and 13). In practice, in most cases the majority of oxidation products that are isolated arise from reaction at the 9- and 13-positions. This selectivity arises because β-scission of the 11-peroxyl radical, the kinetic product, regenerates **20** and O_2_ and is more rapid than trapping of this radical by the diene during propagation. Reaction at the 11-position is only detectable when the initially formed 11-peroxyl radical is trapped by a process that is comparable to or faster than β-scission, such as transfer from a phenolic antioxidant like α-tocopherol (α-TOC). The rate constant for peroxyl radical quenching by α-TOC is 3.5 × 10^6^ M^–1^ s^–1^.^368^ Pratt and Porter have used the α-TOC quenching rate as a radical clock to determine the relative rates of other processes in the propagation pathway and account for the selectivity of peroxyl radical formation. For methyl linoleate at 37 °C, the rate constants for β-scission and trapping of peroxyl radicals by **18** are 2.6 × 10^6^ s^–1^ and 62 M^–1^ s^–1^, respectively.^368,369^ The authors calculated the C–O bond strength for the 11-peroxyl radical to be 8 kcal mol^–1^ weaker than the 9- and 13-peroxy species.^73,368^ At the limit, with very high α-TOC concentrations, the product ratio for 9- vs 11- vs 13-peroxyl radical formation is approximately 1:2:1. This ratio is accounted for by the formation of two energetically equivalent complexes between the pentadienyl radical and O_2_, each complex involving carbon atom 11 and either carbon 9 or carbon 13. The preferred (*E*,*Z*)-configuration of the product **20** in colloidal dispersions may be accounted for by the minimization of steric (gauche) interactions in the transition state of the dienyl radical–oxygen complex formed during O_2_ addition to carbon 9 or 13 and secondary orbital interactions between the π molecular orbitals of ground state ^3^O_2_ and the dienyl radical (Figure 6).

Another propagation mechanism that is suggested from studies on the oxidation of methyl linoleate in micelles is the fragmentation of the peroxyl radical **20** to release a hydroperoxyl radical (HO_2_^•^).^327,367^ Evidence for the formation of HO_2_^•^, which is almost completely dissociated to superoxide at neutral pH, comes from the observation that the enzyme superoxide dismutase (SOD), which catalyzes the conversion of superoxide to oxygen and hydrogen peroxide, significantly inhibits oxidation in these heterogeneous phases. Although peroxyl radical fragmentation to HO_2_^•^ only occurs for PUFAs and is rather slow, with an estimated rate constant of 0.04 s^–1^, it may be significant for the transfer of the propagating chain between vesicles and micelles, a phenomenon that has been observed experimentally.^327^ Simulations suggest that the polar regions of the oxidation intermediates migrate toward the ester regions of the lipids to adopt a disposition closer to the membrane interface, a behavior that reduces the rate of propagation reactions when compared with these reactions in homogeneous phases.^367^

**Scheme 5:**
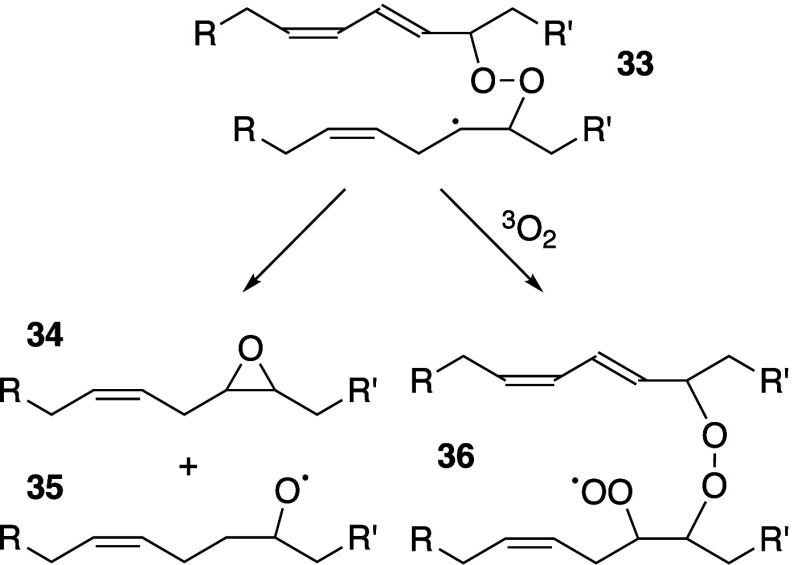
Reactions of Peroxyl Radicals with Neighboring Alkenes

The peroxyl radical **20** may also undergo a peroxyl radical addition to a neighboring alkene to form a new peroxyalkyl radical adduct (**33**, Scheme 5) that can either then undergo further reaction with triplet oxygen to furnish a new peroxyl radical (**36**) or break down *via* an intramolecular homolytic reaction to form an epoxide (**34**) and an alkoxyl radical (**35**). The peroxyl radical addition can make a significant contribution to propagation for PUFAs but is of lower significance for less unsaturated systems.^370^

#### 4.1.3 Termination

Radical combination to form neutral species is the main termination mechanism in the absence of extrinsic agents such as antioxidants.^317^ For membrane lipids, processes involving only fatty acyl groups are not exceptionally well studied, mostly because the lipid dimers and polymeric species that result are challenging to characterize, but are well-known as part of the curing process for drying oils.^371,372^ Radical combination reactions with smaller inorganic radicals, such as those involving the nitrogen dioxide radical to form nitroallyl products and nitroalkenes or nitric oxide to form nitrosoalkenes, have received some attention.^315,373,374^ Other processes contribute to termination, including the Russell mechanism, a disproportionation involving the formation of a tetroxy species (**24**) by recombination of two peroxyl radicals, followed by breakdown of this unstable intermediate to form an alcohol (**25**), a ketone (**26**), and ^1^O_2_.^375^

Radical propagation can also be effectively terminated by redox processes involving nonlipid molecules, including antioxidants such as α-tocopherol,^376−382^ squalenes,^383^ hydropersulfides,^380^ and tryptophan and tyrosine residues in transmembrane proteins.^384^

##### Secondary Processes

**Scheme 6:**
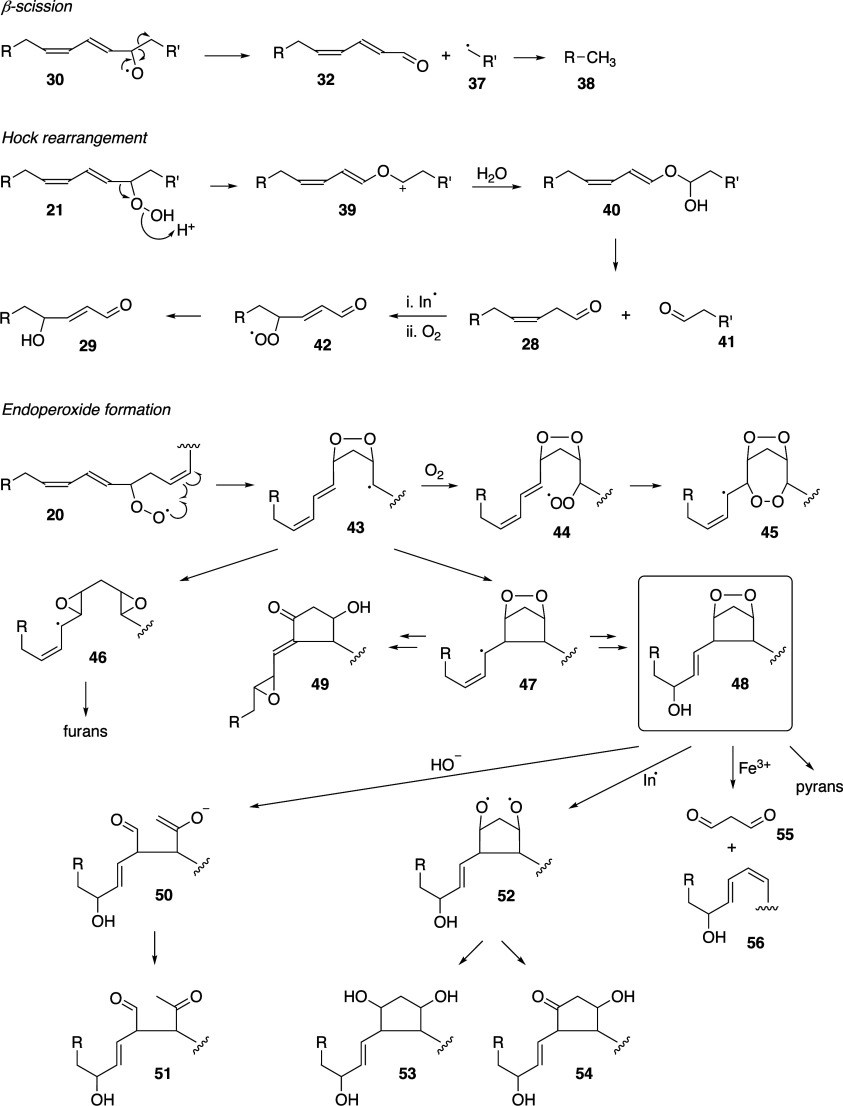
Overview of Key Reactions Involved in Secondary Oxidation

Hydroperoxides (**21**) constitute the major primary products of fatty acid oxidation. A plethora of other oxidized species result from subsequent reactions and rearrangements of these hydroperoxides and their peroxyl radical precursors (Scheme 6). Many of these species are radicals; quenching of these by hydrogen transfer from **18** will also contribute to propagation, and their combination or disproportionation contribute to termination. As with the initially formed 11-peroxy species, β-scission of **20** and recombination of the dienyl radical with O_2_ eventually generates the thermodynamic diene **27**, with an (*E*,*E*)-configuration. The C–O bond strength of the (*E*,*Z*)-peroxyl radical **20** has been calculated to be 1.5 kcal mol^–1^ weaker than that of the (*E*,*E*)-peroxyl radical **27**.^368^

Hydroperoxides are subject to a number of further transformations. In the presence of a reducing metal ion, such as Fe^2+^, disproportionation of the hydroperoxide generates an alkoxy radical (**30**) and hydroxide (analogous to the Fenton reaction). This process, combined with the conversion of **21** to **20** by reduction of Fe^3+^ described above, provides a cycle for hydroperoxide degradation that is metal-catalyzed. In the absence of a metal ion, homolytic hydroperoxide fragmentation can be achieved photochemically to generate **30** and a hydroxyl radical.^385^ This alkoxy radical can then either form the corresponding alcohol by hydrogen transfer or undergo β-scission to form the conjugated aldehyde **32**, with loss of an alkyl radical. Hydrogen transfer to the alkyl radical ultimately forms either an alkane or a truncated acyl group, depending on which side of the double bond the alkoxy radical is generated. Alternatively, intramolecular *S*_H_*i* reaction of the alkoxy radical generates the epoxide **31**. Hydrolysis of one epoxide of **31** to form the alcohol and subsequent attack of this alcohol on the other epoxide generates a tetrahydrofuran.

**Figure 7:**
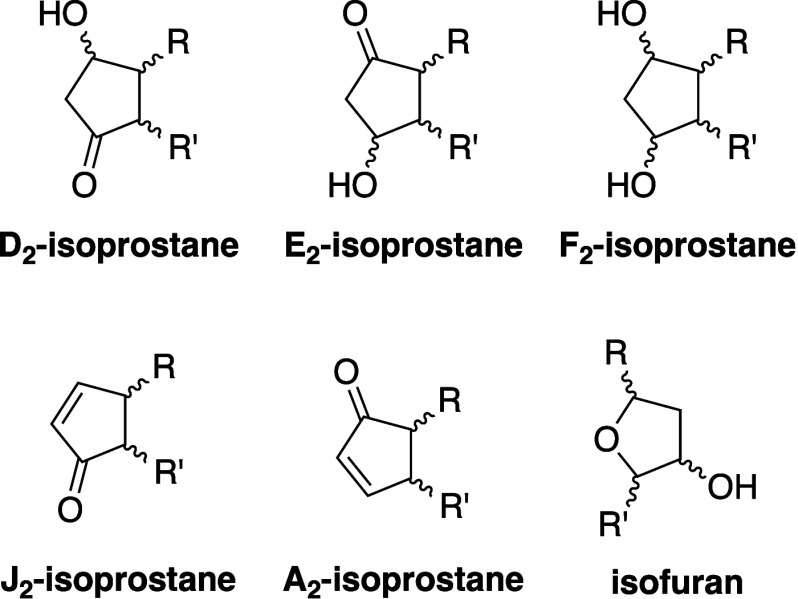
Isoprostanes and isofurans formed by fragmentation of endoperoxides. R and R′ correspond respectively to the groups proximal to the carboxyl and methyl ends of the parent fatty acid.

Under acidic conditions, the Hock rearrangement generates a vinyl oxonium species **39** (Scheme 6) through alkyl migration to the proximal peroxyl oxygen with concomitant loss of water.^386^ Reaction of this cation with water generates a hemiacetal (**40**), which after hydrolysis produces the aldehyde **41***via* its acetal, and the homoconjugated aldehyde **28**. Further reaction of **28***via* allyl radical formation and reaction with O_2_ generates the peroxyl radical **42**, which ultimately forms either the corresponding hydroperoxide or alcohol **29** by peroxyl cleavage. γ-Hydroxy-α,β-unsaturated aldehydes **29** are key products of fatty acid oxidation, with 4-hydroxynonenal (4-HNE), formed by cleavage of Ω-6 fatty acids at carbon n-9, being one of the most abundant and most well studied.^387^ If an additional homoconjugated double bond is present in the R′ group of **20**, intramolecular reaction of the peroxyl radical generates an endoperoxide and the alkyl radical **43**. This requirement for homoconjugation in R′ makes this 5-*exo* cyclization reaction of particular importance for PUFAs. The rate constant for this process is comparable to that of β-fragmentation to generate the (*E*,*E*)-diene (described above), and as a consequence (*E*,*E*)-isomers are infrequently observed.^317^ Alkyl radical **43** is a key intermediate that can react further *via* a number of alternative pathways depending on conditions. Reaction with O_2_ generates peroxyl radical **44**, which can subsequently from further macrocyclic endoperoxides such as **45**. With additional unsaturation in R′, the process can be repeated to form products with multiple endoperoxyl groups. Ultimately, rearrangements of these endoperoxides generate isofurans and isoprostanes (Figure 7).^111^

Under less oxidizing conditions, intramolecular rearrangement of **43** generates the bis-epoxy radical **46**. Ring closing of **43** generates spirocyclopentane **47** with an exocyclic allyl radical center. Compound **47** is a precursor for the D_2_-isoprostane **49** (and corresponding J_2_-isoprostane, not shown). Reaction of the allyl center of **47** with O_2_ and reductive cleavage of the hydroperoxide generates allyl alcohol **48**. This alcohol is another key intermediate in the generation of complex oxidation products.^388^ Under basic conditions, **48** undergoes ring opening to generate enolate **50** and ultimately the highly toxic^325,389^ γ-ketoaldehyde **51**. Under suitable conditions for radical initiation, homolytic cleavage of the O–O bond of **48** generates the bis-oxy radical **52**. This ultimately forms the diol **53** by hydrogen transfer under or the D_2_-type isoprostane **54** under more oxidizing conditions. The corresponding E_2_-isoprostane is also formed. Both D_2_- and E_2_-isoprostanes are further modified by dehydration to form the corresponding unsaturated ketones (J_2_- and A_2_-isoprostanes, respectively). In the presence of metal ions, **48** undergoes a ring opening reaction to generate an alkene (**56**) and one equivalent of malondialdehyde (**55**). Along with 4-HNE, malondialdehyde is one of the key tracers used to monitor fatty acid degradation in liposomes and foodstuffs. Given the variety of naturally occurring fatty acids, it is unsurprising that a range of other volatile products have been characterized following lipid oxidation, including aldehydes, carboxylic esters, alcohols, and hydrocarbons.^390,391^ Many of these contribute to the generation of bad aromas and flavors during oxidative processes in foodstuffs^70,178,392,393^ or the generation of distinctive colors and aromas during cooking.^390,394−396^ As apparent by the reactions in Schemes 4 and 6 that involve metals, as well as the role of metals in the generation of ROS, oxidative reactions are a particular issue for foodstuffs containing high levels of iron.^338,397−399^

Lipid oxidation in foodstuffs, when measured by a range of indices, such as the polyene index, peroxide value (PV), or thiobarbituric acid reactive substances (TBARS), occurs to a more significant extent than hydrolysis.^255^ While there are obvious variations in oxidation rate according to the nature of the material and how it is treated, the level of oxidized PUFAs tends to increase steadily up to 15 days or more.^400,401^ For example, recent studies using bogues found a ∼10% decrease in PUFA content after 16 days at 0 °C compared to 3% FFA formation.^401^ Oxidation rates decrease at low temperatures and can be further decreased by the exclusion of oxygen or the inclusion of antioxidants.^402^ Other treatments such as high-pressure processing (>300 MPa) have been found to increase rates of oxidation, an effect that may be a consequences of changes to the speciation of metal ions in the system.^72^

The aldehydes produced by secondary oxidation are significant because of their reactions as electrophiles with other macromolecules, including DNA, proteins, and lipids.^81,132−134,389,403−405^ Such reactions occur either directly through the carbonyl group (imine formation) or *via* the Michael reaction for α,β-unsaturated aldehydes. Reactions with aldehydes are responsible for the formation of pyrroles through processes such as the Maillard and Strecker reaction.^394,406−409^ An alternative breakdown pathway from hydroperoxide **21** leads to the formation of the keto equivalent of 4-HNE, 4-oxononenal (4-ONE), which also undergoes facile pyrrole formation.^410^ Although these reactions are usually assumed to require significant heating (*e.g*., during food cooking), their products have been detected at lower temperature (25–37 °C).^411^ Other aromatic species such as thiazolidines, furans, and thiophenes can be formed by reaction of aldehydes with peptides bearing an N-terminal cysteine, such as the Cys-Gly sequence derived from glutathione.^412^ In addition, thiol-containing species such as glutathione are able to form adducts with cyclic peroxides such as **48** by direct nucleophilic attack, as well as their α,β-unsaturated counterparts (*e.g*., **54**) by conjugate addition.^413^ Nitroalkenes, formed by radical combination reactions, are reactive electrophiles that may also undergo similar reactions with the thiol group of cysteine as a potential nucleophile.^414^

The relationship between the Maillard reaction and lipid oxidation products is not straightforward. In the first instance, the thermal degradation of monounsaturated fatty acids (MUFAs) *via* hydroperoxides varies with lipid class, being markedly slower and giving a different product profile for PC and PE when compared with the free fatty acids or triglycerides. Volatile carbonyl compounds derived from lipid oxidation provide additional sources of reactive carbonyl species to those formed by the Amadori rearrangement for the final stages of the Maillard process. However, radical intermediates in the oxidation of lipids also influence the balance of carbonyl compounds formed by the Amadori rearrangements^415^ and the overall rate of the Maillard process. Lipid oxidation and the Maillard reaction exhibit and interdependency where the rate of one influences the rate of the other, either positively or negatively depending on the circumstances.^394^

**Scheme 7:**
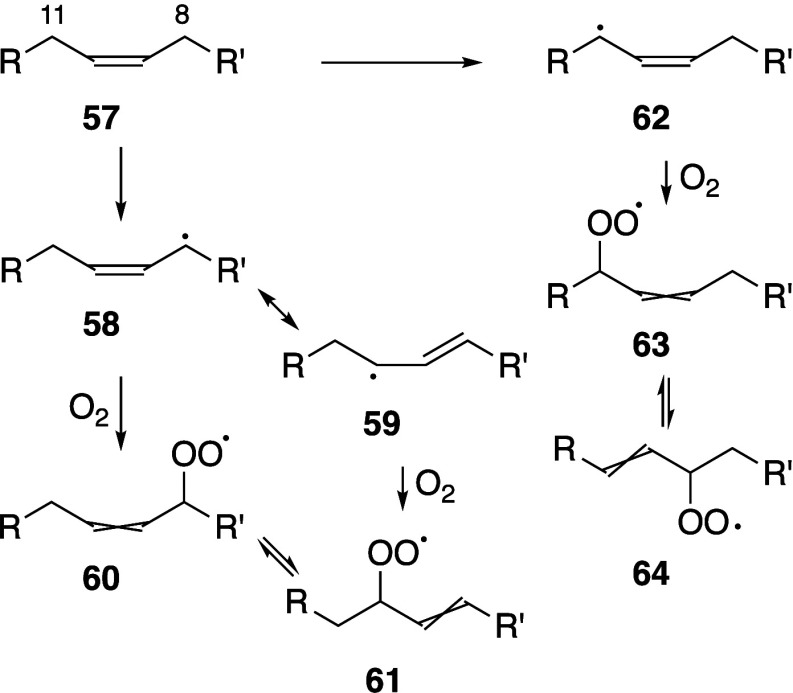
Reactions of Isolated Alkenes The numbering corresponds to the carbon atom numbering of oleic acid.

Monounsaturated fatty acids (MUFAs) are significantly less reactive toward autoxidation than PUFAs. Indeed, when the fatty acyl composition of liposomes is enriched in oleoyl groups, the corresponding rate of oxidation of PUFAs in the same systems decreases, pointing to an antioxidant activity.^416^ Nonetheless, MUFAs are able to undergo similar autoxidation reactions to PUFAs, involving the abstraction of a hydrogen atom during initiation to form a radical at either of the allylic positions (**58** and **62**, Scheme 7, corresponding to carbon atoms 8 and 11 of oleic acid) that then form (*Z*)-peroxyl radicals by reaction with ^3^O_2_ as kinetic peroxide products (**60** and **63**).^339,417−419^ Both of these allylic radicals have corresponding resonance forms, exemplified by **59** for **58**, that can also form peroxyl radical products, giving the corresponding (*E*)-peroxyl radicals as additional kinetic products (**61** and **64**, corresponding to carbon atoms 10 and 9 for oleic acid, respectively). Allylperoxyl radicals can undergo radical mediated [2,3] rearrangements, so that the initially formed peroxyl radicals can interconvert reversibly between **60**/**63** and **61**/**64** to form the (*E*)-isomers of peroxyl radicals **60** and **63** and the (*Z*)-isomers of **61** and **64**. Ultimately, considering the geometric isomers of the alkene, this gives rise to eight potential peroxyl radicals (and corresponding hydroperoxides). The distribution of products is dependent on experimental conditions, including temperature, substrate choice, and the mode of oxidation. In model reactions with methyl oleate at 25 °C, formation of the 8-peroxyl product is marginally favored over formation of the 10-peroxyl product, with the 8-peroxyl product comprising approximately equal amounts of (*E*)- and (*Z*)-isomers and the 10-peroxyl radical almost entirely *E*. At higher temperatures the stereochemical fidelity of the rearrangement is reduced, which along with *ab initio* calculations has led a proposed mechanism involving dissociation of the peroxyl radical to from an allyl radical–oxygen complex.^368^ Escape of oxygen from this complex competes with collapse to the rearranged peroxyl radical. Ultimately, the peroxyl radicals **60**, **61**, **63**, and **64** undergo similar trapping and termination reactions to those in Scheme 4. On the other hand, in another study, air oxidation of methyl oleate at 60 °C gave predominantly (*E*)-hydroperoxides at the 8-, 9-, and 10-positions as the major products (although the 9- and 10-isomers could not be separated).^417^ The product profile may can give information on the mode of oxidation to which a sample has been subjected. For example, in senescent cells, type II photosensitized oxidation of MUFAs at UV wavelengths forms all eight potential hydroperoxides; similar oxidation at visible wavelengths forms predominantly the (*E*)-isomers corresponding to oxidation at the 9- and 10-positions of oleic acid ((*E*)-**61** and (*E*)-**64**, respectively), and autoxidation favors *E*/*Z* mixtures corresponding to oxidation at the 8- and 11-positions of oleic acid (**60**/**63**) alongside the (*E*)-isomers of **61**/**64**.^418^ Thus, the (*Z*)-isomers of **61**/**64** are indicators of photooxidation at UV wavelengths, and the (*Z*)-isomers of **60**/**63** are markers for either autoxidation or photoxidation at UV wavelengths.

#### 4.1.4 Diacylglycerophospholipids Other than Phosphatidylcholine

**Scheme 8:**
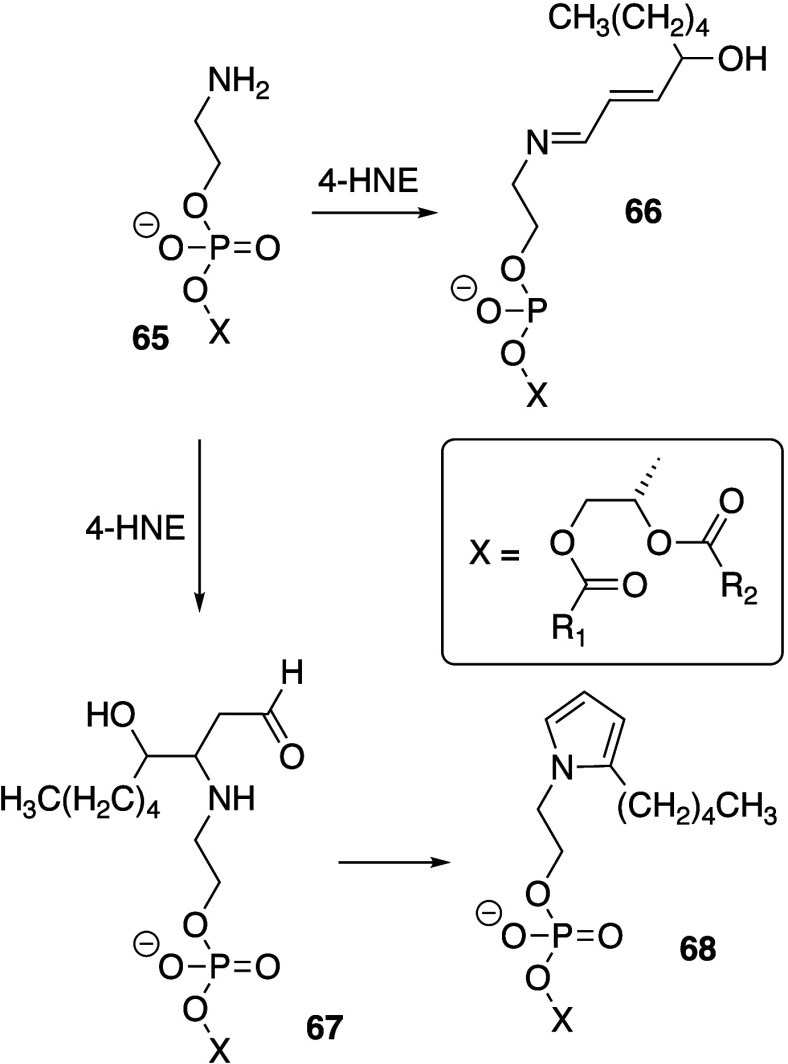
Products Formed by the Reaction of PE Lipids with 4-HNE

Similar oxidation reactions to those of PC fatty acyl groups are seen with the acyl groups of PE, PS, PG, phosphatidylinositols (PIs), and PA.^74,420,421^ The amino groups of PE and PS are also potential reaction sites. For example, *N*-chlorination of PE *via* reaction with hypochlorous acid has been detected.^81^ Recently, reactions of PE, and to a lesser extent PS, amino groups with the reactive carbonyls produced by fatty acid oxidation have been described with increasing detail.^325,421−426^ For example, incubation of PE with 4-HNE in a two-phase ether/water system at 30 °C for 2 h led to the formation of the Schiff base adduct **66**, together with the Michael adduct **67** and the pyrrole **68** formed from the latter (Scheme 8). Product abundances decreased in the order **68** > **67** > **65** ≫ **66**. PS underwent similar Michael reactions with 4-HNE but was less reactive; consequently, the pyrrole and Schiff base products were not detected.^426^ Under similar conditions, γ-ketoaldehydes (**a28**) undergo Schiff base formation with PE, also leading to the formation of pyrroles as an end point.^427,428^

**Figure 8:**
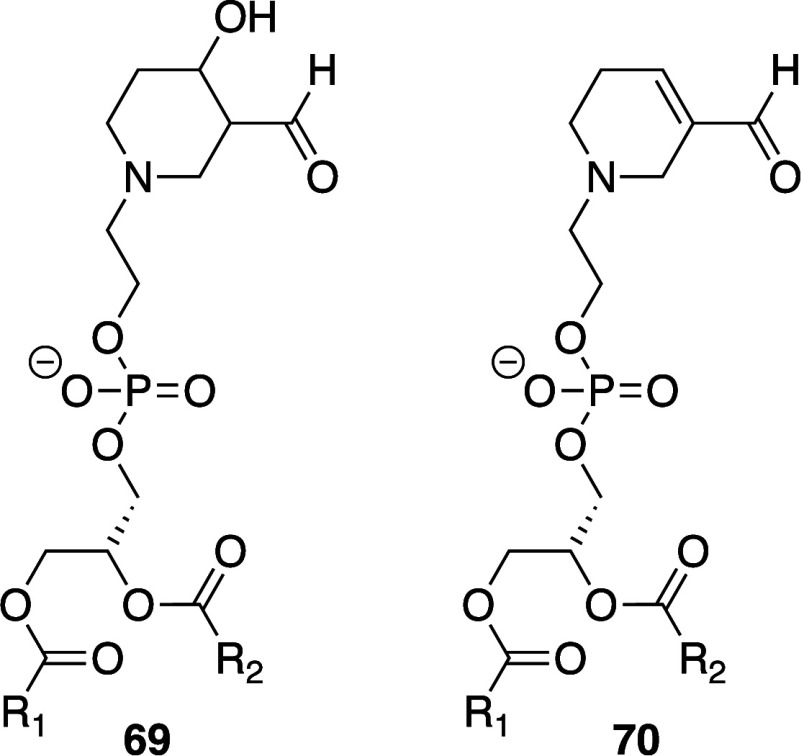
Amino-modified PE lipids isolated from HP-60 cells after exposure to acrolein.

In a human retinal pigment epithelial cell line, 4-hydroxy-7-oxohept-4-enoic acid is formed from α,β-unsaturated aldehyde equivalent to **29**, where the R group is a glyceryl ester. The lactone form of this hydroxy acid is able to modify PE and protein amino groups to form 2-carboxyethyl pyrroles.^427^ The products of alkenal addition to PE have been detected as trace products (0.01–0.02% of total PE) in rat retinas, increasing to 0.4–1% under conditions of diabetes-associated oxidative stress.^429^ Piperidines **69** and **70** (Figure 8), formed by consecutive Michael reactions of PE with acrolein followed by ring closure, have been detected in membrane extracts of HP-60 cells following their exposure to acrolein.^430^ Other experiments have detected amino-functionalized ethanolamines, breakdown products of N-modified PE, in red blood cell lipid extracts,^431^ mitochondrial membranes,^432^ and urine.^433^

**Figure 9:**
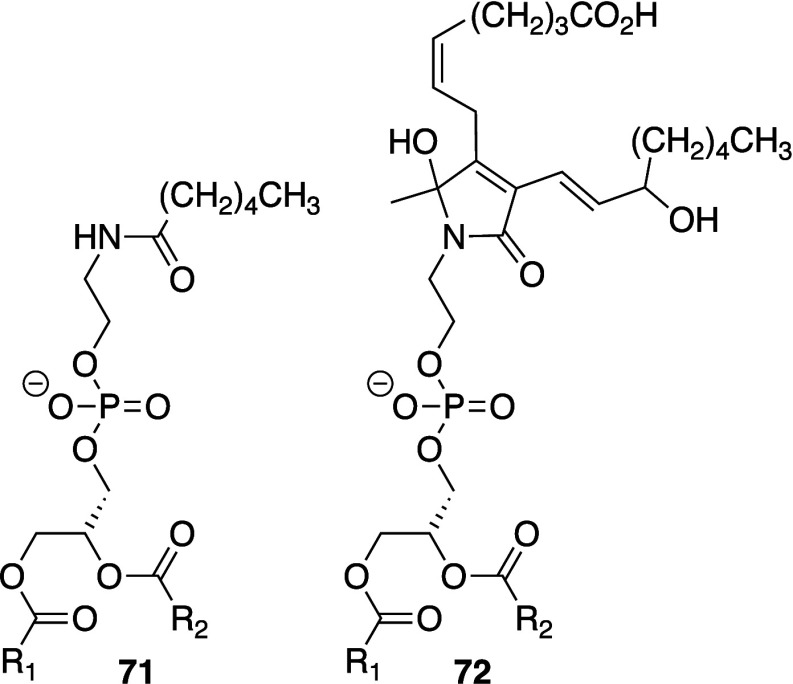
Products formed by the reaction of PE with reactive oxygen carbonyl compounds derived from fatty acid oxidation.

Model experiments using liposomes composed of 1-stearoyl-2-arachidonyl-*sn*-glycero-3-phosphocholine (SAPC) and DPPE in the presence of *t*-butylhydroperoxide or Cu(II)/H_2_O_2_ initiators led to the formation of a number of new species resulting from PE amino group reactions with intermediates generated from arachidonate.^423^ Along with compounds of the type described above (Figure 8 and Scheme 8) and cross-linked products arising from reaction with malondialdehyde (**53**), *N*-acyl compounds such as *N*-hexanoyl-PE (**71**, Figure 9)^434^ and products resulting from reaction with prostaglandin-type oxidation products (**72**) could also be identified. Recent experiments suggest that exogenously added prostaglandins are more reactive toward PE than proteins.^435^

In a model system comprising dipalmitoyl-*sn*-glycero-3-phosphocholine (DPPE), glucose, and the Fenton reagent, the hydroxyl radical was found to oxidize glucose to glyoxal and methyl glyoxal, which subsequently combined with the lipid to form carboxymethyl PE and carboxyethyl PE. Carboxymethyl PE was also formed by oxidation of the Amadori products by the hydroxyl radical.^436^ A number of other experiments have provided secondary evidence for the presence of amine-modified PE lipids, such as the development of fluorescent chromophores following peroxidation of both model lipid mixtures and biological membranes *in vitro* (reviewed in ref (424)). Oxidation of hemiketal intermediates formed by the reaction of amino groups with reactive aldehydes formed from cholesterol^437^ and fatty acids^438^ have recently been described, leading to the formation of amides. It is reasonable to expect that the amino groups of PE lipids may undergo similar reactions.

**Scheme 9:**
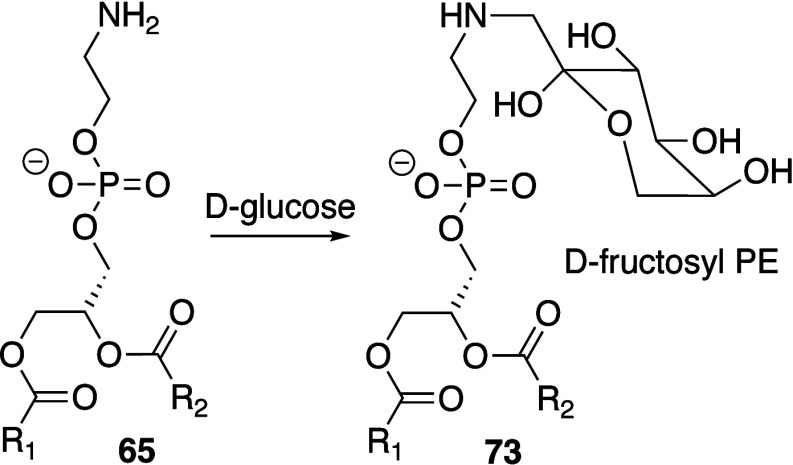
Amadori Rearrangement Product Formed by the Reaction of Glucose with PE

The products of lipid amine reactivity with carbohydrates and their autoxidation products have been detected *in vivo*. In a similar fashion to the Maillard reaction, reducing sugars such as glucose form a Schiff base with the PE amino group. Subsequent Amadori rearrangement of this imine leads to the formation of a ketosamine (**73**, Scheme 9). This amine is a precursor for several subsequent transformations, including oxidative degradation to form *N*-carboxymethyl- and *N*-carboxyethyl-PE.^424,439^ Autoxidation of glucose (Wolff pathway) or **73** (Namiki pathway) leads to the formation of a number of carbonyl species that are reactive toward aminophospholipids, including glyceraldehyde, glyoxal, and methyl glyoxal.^439^ Reactions of carbohydrate- and lipid-derived aldehydes and ketones with proteins and lipids ultimately leads to the formation of advanced glycation end products (AGEs) and advanced lipoxidation end products (ALEs), which are indicators of aging and are implicated in age-related diseases.^440^ In the case of lipids, AGEs are potential markers for diabetes.^439^

A study of the lyso-PE and PE lipids (including plasmanyl and plasmenyl) in human plasma that have been modified by the Amadori reaction identified 33 lyso-PEs and 142 PE products. Of these, the majority of lyso-PE- and PE -derived products were present at sub-nM levels, including all plasmanyl-PEs (PE O). The most abundant species detected, with plasma concentrations >5 nM, were the plasmalogens PE P-16:0/20:4, PE P-18:0/20:4, and PE P-18:1/20:4 and the lipids PE 18:0/20:4, PE 18:0/18:2, and PE16:0/22:6. By comparison with the corresponding unmodified PE lipids in the sample, the glycation rate was estimated at approximately 0.07%.^441,442^

Given the role of mitochondria in respiration and the generation of ROS, it is unsurprising that attention had been given to the oxidative stability of mitochondrial lipids. Particular attention has focused on PE lipids and cardiolipins (CDLs), which are major components of mitochondrial membranes, and their reaction with perhydroxyl radicals formed in the proximity of the inner membrane from the superoxide radical.^443^ CDLs are found predominantly in the inner mitochondrial membrane, with an asymmetric distribution skewed to the leaflet that faces the mitochondrial matrix. The fatty acid content of CDLs is complex. In many organs, CDLs contain a broad range of fatty acids, including PUFAs, in which each of the four acyl chains is different, with tetra-C20:4 being the predominant CDL. In others, including the heart, muscles, and the liver, the fatty acid distribution is remodeled to favor mostly C18:2 fatty acids. During aging and diabetes, the fatty acid composition of CDL in heart tissues is remodeled in favor of chains with increasing levels of unsaturation.^443^ Even though CDLs are of relatively low abundance compared to other mitochondrial glycerophospholipids, their fatty acids are significantly more susceptible to oxidation and are often the first lipids oxidized under oxidative stress.^358,443^ This susceptibility is attracting attention as it potentially indicates a role for CDL in cell regulatory responses and signaling. Following trauma such as brain injury, for example, >100 different oxidized CDLs can be formed.^444^ The range of species found includes many of those in Scheme 4, including hydroperoxides, alcohols, epoxides and aldehydes.^445^ While much of this oxidation is under enzymatic control, the proportion of the array of resulting oxidized CDLs that has physiological relevance is still unclear. Oxidation leads to CDL redistribution, with a loss of membrane asymmetry, and is sometimes accompanied by increased phospholipase-mediated ester hydrolysis to form monolyso CDLs.^64^

### 4.2 Plasmalogens

**Scheme 10:**
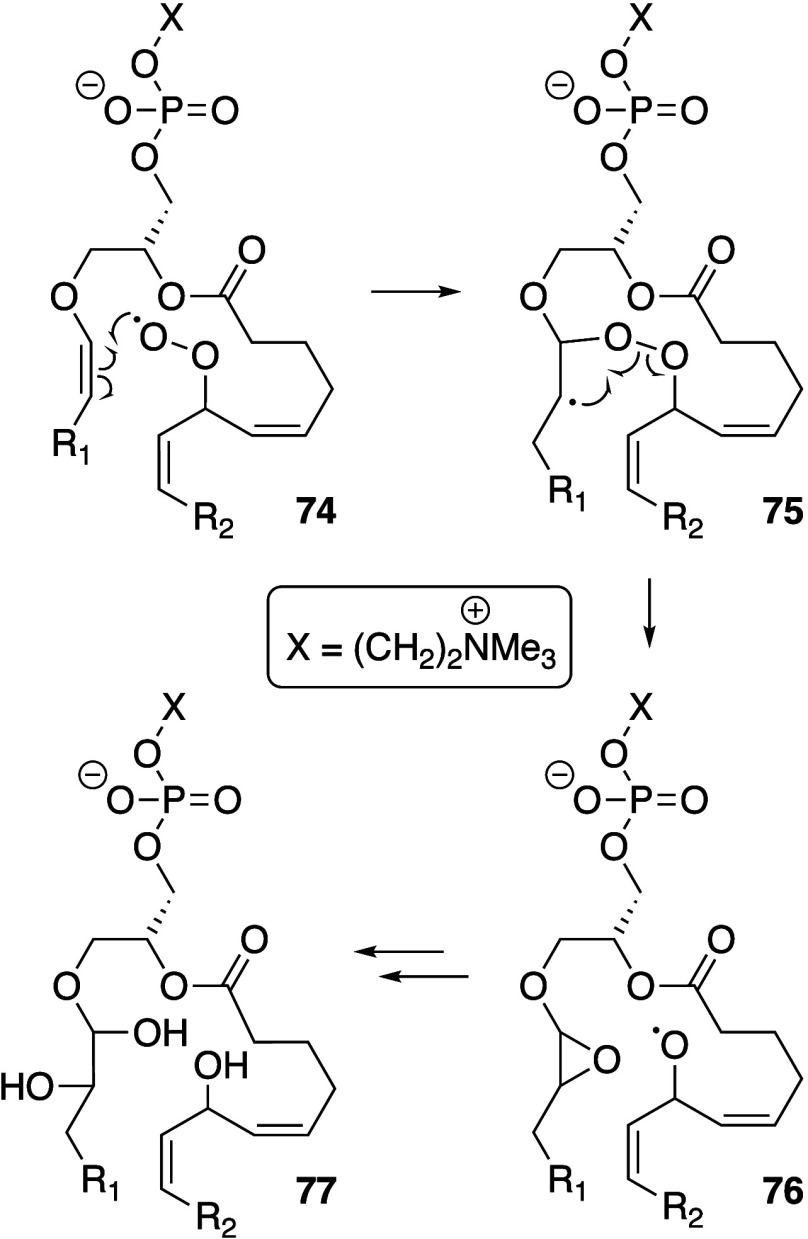
Oxidation of Plasmalogens with a PUFA at the *sn*-2 Position The double bond pattern shown here is that of arachidonic acid.

Plasmalogens are distinguished by the presence of an enol ether at the *sn*-1 position of the lipid. Oxidation of this alkene to form an epoxide occurs when a PUFA is present at the *sn*-2 position (Scheme 10).^446^ The peroxyl radical formed in the *sn*-2 chain (**74**) adds to the enol ether *via* alkylperoxy intermediate **75** to form the alkoxy radical **76**. Trapping of the alkoxy radical and hydrolysis of the epoxide forms the acetal **77**, which subsequently hydrolyzes to form an α-hydroxyaldehyde and a lyso-PC.^122^ The aldehyde product is able to form Schiff base with the amino group of PE and PEpl.^447,448^ Oxidation with Fe^2+^ and ascorbate provides an alternative route to the epoxide of the enol ether for plasmalogens without a PUFA at the *sn*-2 position.^447,449^

### 4.3 Sphingolipids

**Figure 10:**
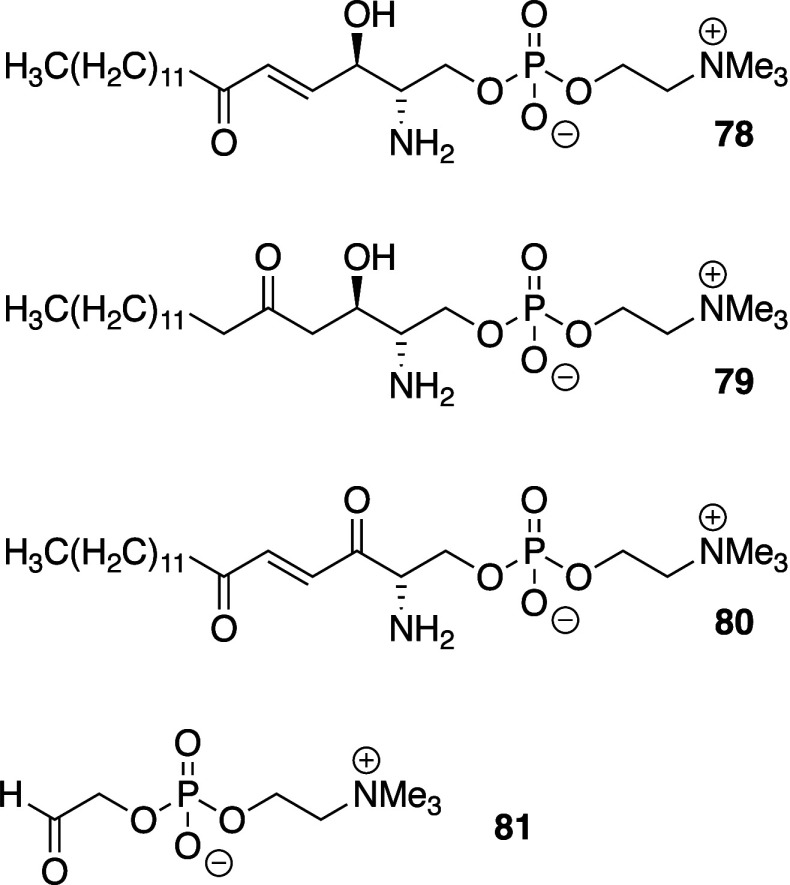
Products formed by the oxidation of sphingolipids.

The alkene of the sphingosyl group in these lipids is significantly more resistant to radical oxidation than the alkenes of plasmalogens and sterols. In recent work, oxidation of lactosyl and galactosyl ceramides using hydroxyl radicals generated by the Fenton reaction gave oxidation products on unsaturated fatty acyl chains, forming allyl hydroperoxides, allyl alcohols, and α,β-unsaturated ketones.^450^ If the fatty acyl chain was saturated, the only products were loss of the sugar group to form ceramide. Under similar conditions, ceramide was found to be unreactive, whereas sphingomyelin underwent acyl cleavage to the amine (sphingosylphosphorylcholine, SPC), and subsequent oxidation of SPC around the sphingosyl alkene to form **78**–**80** (Figure 10), as well as cleavage between C2 and C3 (**81**).^451^

### 4.4 Cholesterol

**Figure 11:**
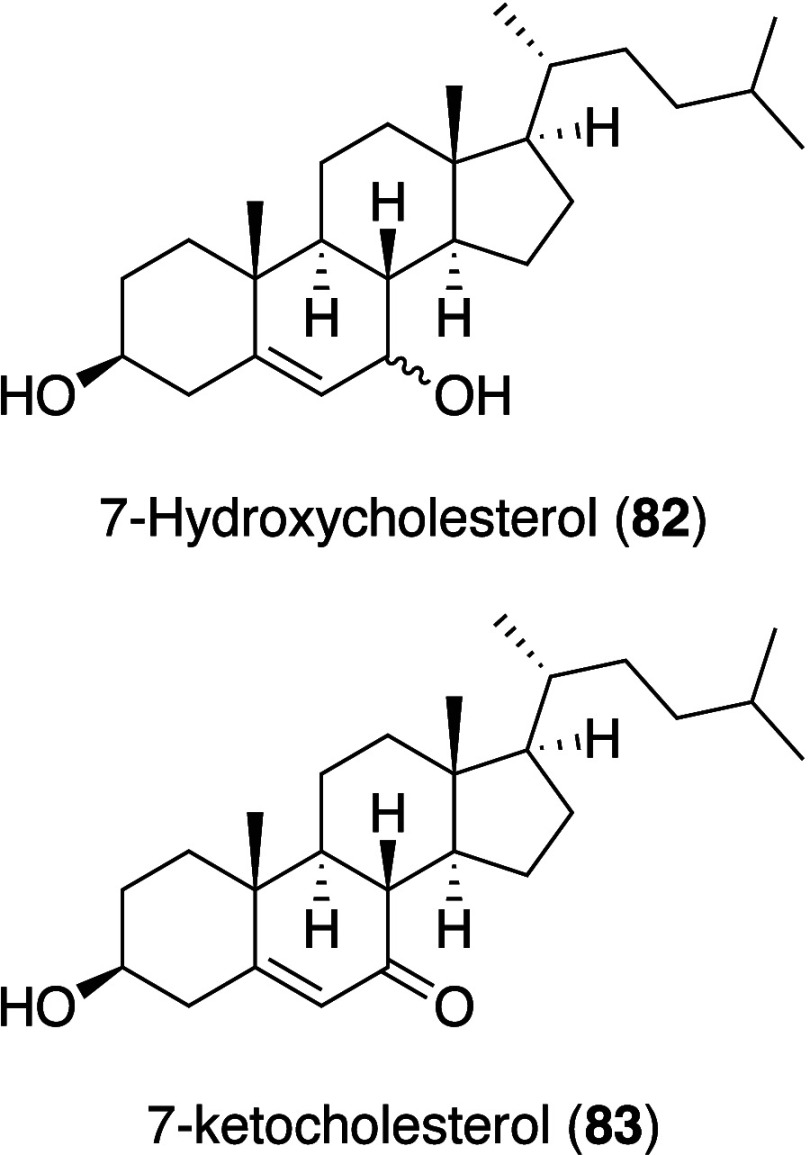
Predominant products arising from the autoxidation of cholesterol.

The nonenzymatic oxidation of cholesterol has been comprehensively reviewed.^452^ Due to the location of the alkene at the bridgehead between the A and B rings, cholesterol is marginally more reactive toward oxidation than the isolated double bond of monounsaturated fatty acids.^453^ In saturated lipid membranes, cholesterol undergoes slow oxidation, and cholesterol inclusion in most unsaturated membranes leads to a reduction in the rate of oxidation. Reactions at sites on the alkyl side chain are known, notably at the 24- and 25-positions, but are usually enzyme mediated and of little significance in autoxidation.^319,382,454−457^ Abstraction at more esoteric positions, such as C15, can be promoted in constrained environments, but it remains to be seen whether this can occur in membranes.^458^ Autoxidation of cholesterol leads to hydroperoxylation and hydroxylation, predominantly at the 7-position around the sterol core. The α- and β-isomers of 7-hydroxycholesterol (**82**, Figure 11) can be converted into 7-ketocholesterol (**83**) enzymatically in the liver, or nonenzymatically by a disproportionation reaction between peroxyl radicals that leads to the formation of **83**, an alcohol, and ^1^O_2_ (Russell mechanism).

**Figure 12:**
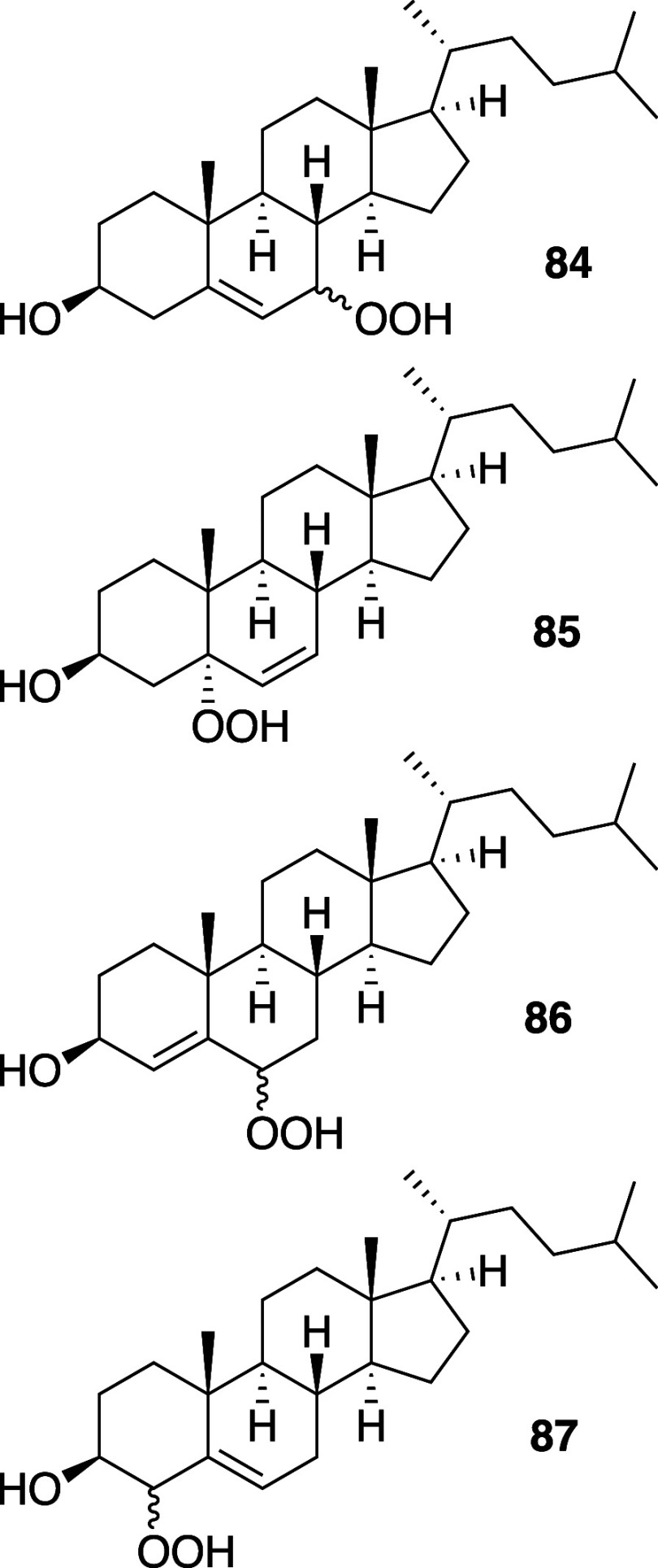
Oxidation products arising from the autoxidation of cholesterol at the 7-position of the sterol ring.

The predominant product forms by reaction at the 7-position to form the hydroperoxide (**84**, Figure 12) *via* an intermediate 3-centered allylic radical involving carbon atoms C5–C7. In model reactions in chlorobenzene, the C4 oxidation product (**87**), arising *via* the alternative 3-centered radical involving C4–C6, is formed with a lower abundance to the C7 product, with the C6 oxidation product (**86**) having the lowest abundance of those observed.^459^

**Figure 13:**
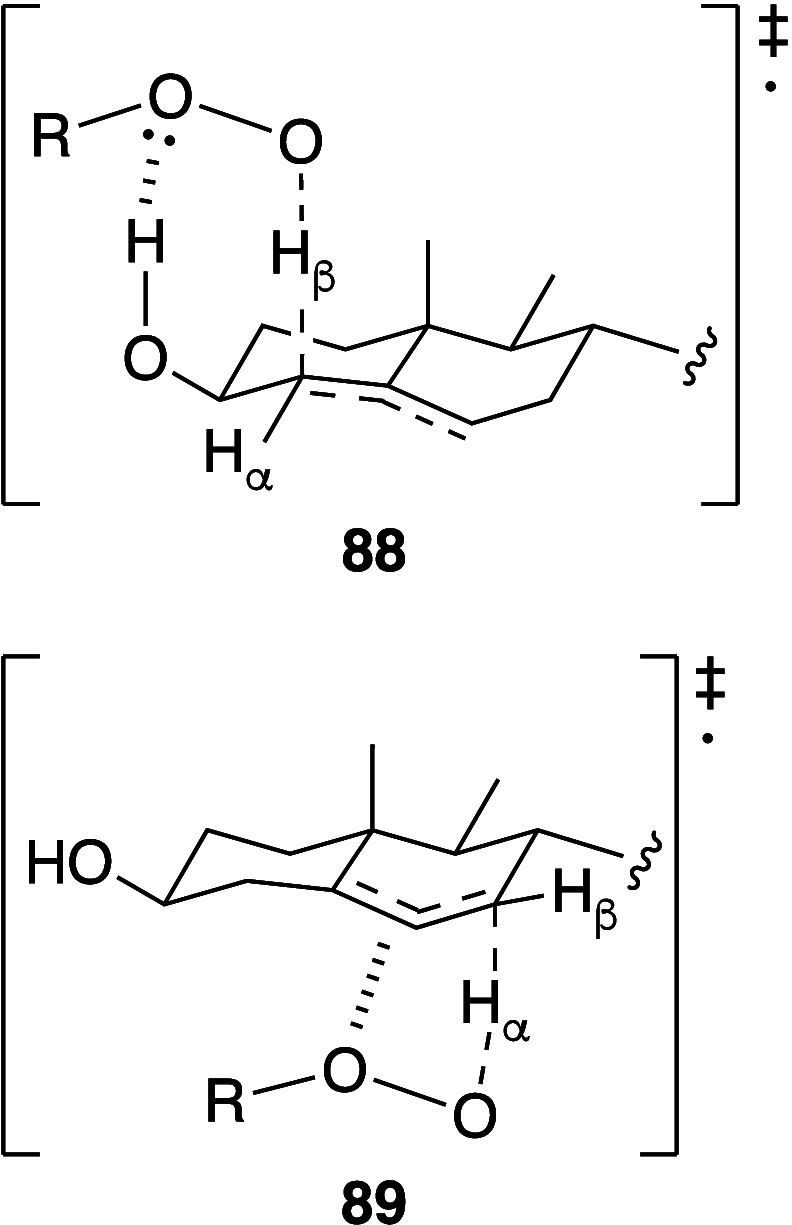
Transition states for hydrogen abstraction by peroxyl radicals during the propagation stage of cholesterol autoxidation.^459,460^

The 5-hydroperoxides (**85**) are not normally observed because β-scission of the 5-peroxyl radical is faster than trapping during the propagation step. In a similar fashion to their trapping experiments described above for methyl linoleate, Zielinski and Pratt have recently demonstrated that reactions of cholesterol in chlorobenzene in the presence of either a truncated α-TOC analogue or 4-*tert*-butyl-2,6-dimethylphenol (BDMP) enable trapping of C5-peroxyl radical to form the corresponding hydroperoxide as a mixture of α- and β-isomers. The amounts of the 5-hydroperoxide formed were small but increased linearly with the concentration of BDMP and enabled the rate constant for β-scission of the 5-peroxyl radical to be determined as 5.6 × 10^5^ s^–1^.^459^ The equivalent rate constant for fragmentation of the 4-peroxyl radical was 8.6 × 10^3^ s^–1^. The ratio of products formed *via* abstraction at C4 vs C7 was found to depend on the concentration of BDMP. Density functional theory (DFT) calculations suggest that abstraction of the α-H at C7 is favored by steric hindrance with the β-methyl at C10 and secondary orbital interactions between the π-bond of the alkene and the part of the SOMO centered on the internal oxygen (**89**, Figure 13).^460^ Abstraction of β-H at C4 is favored by hydrogen bonding between the C3-OH and the incoming peroxyl radical during the propagation step (**88**). This hydrogen bonding lowers the barrier for hydrogen atom abstraction at C4 to a value not hugely greater than that for abstraction at C7. At high BDMP concentrations, the BDMP radical becomes the predominant radical species present, preventing the hydrogen bonding mechanism that supports abstraction of the β-H from C4 from operating.

**Scheme 11:**
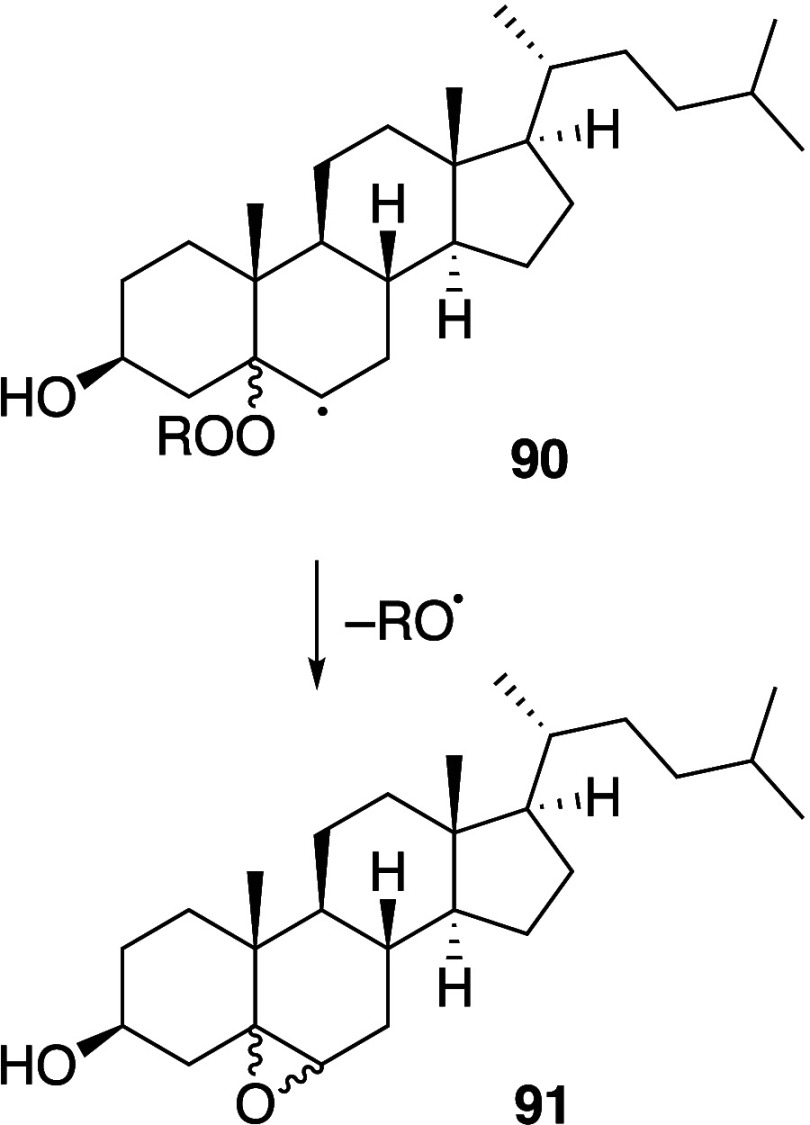
Generation of 5,6-Epoxycholesterol (**91**)

An alternative free radical route to oxidized sterols is *via* reaction with a lipid peroxyl radical to form the 5-peroxyl radical intermediate **90** (Scheme 11), which is subsequently transformed into 5,6-epoxycholesterol (**91**).^461^ Peroxyl radical addition at C5 occurs preferentially on the α-face of the structure.^460^

**Figure 14:**
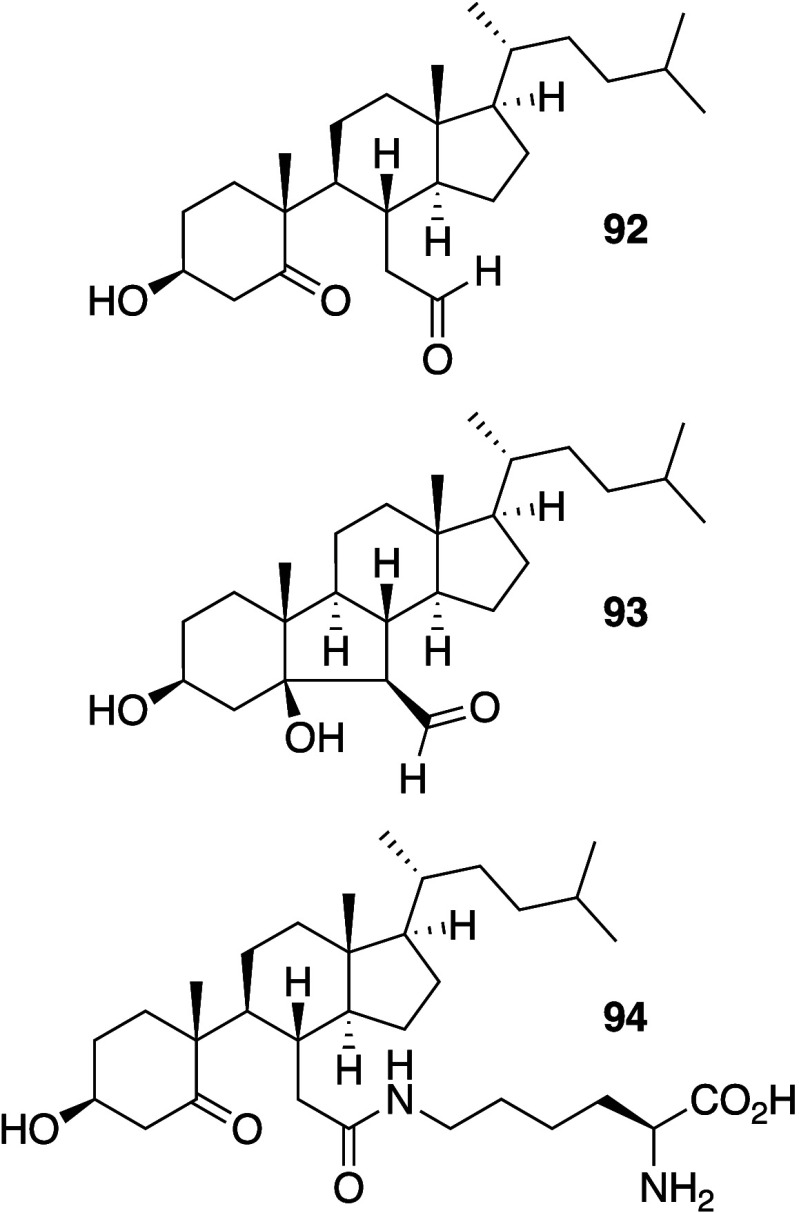
Higher-order cholesterol oxidation products.

Higher-order oxidation products also form (Figure 14), such as secosterol (**92**) and its aldol condensation product **93**. These can be formed by a number of routes, including ozonolysis, reaction of cholesterol with ^1^O_2_, and Hock rearrangement of the α-isomer of **85**.^462^ Amides such as **94** have been detected during the photosensitized oxidation of cholesterol and are proposed to form *via* oxidation of the hemiaminal intermediate formed by the reaction of the terminal amino group of lysine with the aldehyde group of **93**.^437^

Formation of Schiff bases between cholesterol aldehydes and proteins leads to changes in protein folding that are potentially important in the development of disease.^453,463^ Ketosterols are generally unreactive toward Schiff base formation.

**Scheme 12:**
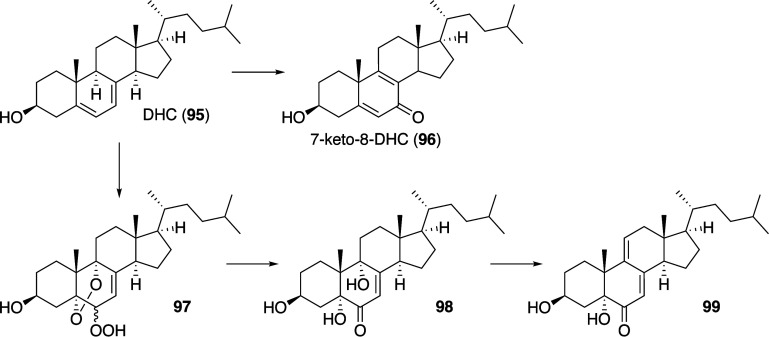
Products Arising from the Oxidation of Cholestadiene

Cholestadienes are metabolic precursors to cholesterol and vitamin D_3_. Their levels are usually low *in vivo*, but as they are metabolic precursors to cholesterol and vitamin D_3_ they can accumulate in some circumstances. Incorporation of a second alkene into the sterol core, particularly when conjugated or homoconjugated to the C5–C6 alkene, significantly increases reactivity toward autoxidation.^453^ For example, the propagation rate constant for 7-dehydrocholesterol (DHC, **95**) is 2260 M^–1^. Autoxidation of DHC leads to the formation of numerous (>10) oxysterol species (Scheme 12), mostly through reaction at C5 and C9, of which keto-dehydrocholesterols **96**, **98**, and **99** are significant examples. The latter two are formed *via* the cyclic peroxide **97**. The 6-alcohol corresponding to **97** is also detectable as a reaction product.^127,464,465^

**Figure 15:**
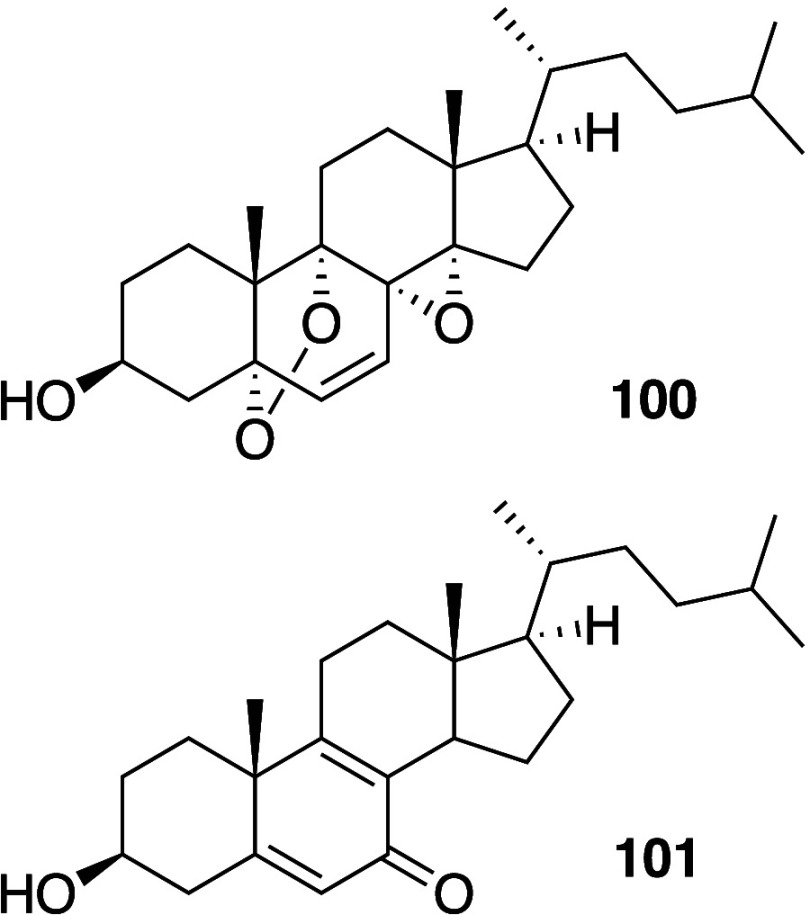
Products arising from the oxidation of cholestadiene following hydrogen atom abstraction at C14.

Sterols **96**, **98** and **99**, formed by initial hydrogen atom transfer at the 9-position, are favored in the presence of α-tocopherol in solution. Initial hydrogen atom abstraction at the 14-position leads to **100** and **101** (Figure 15).^466^

### 4.5 Nonradical Oxidation

**Scheme 13:**
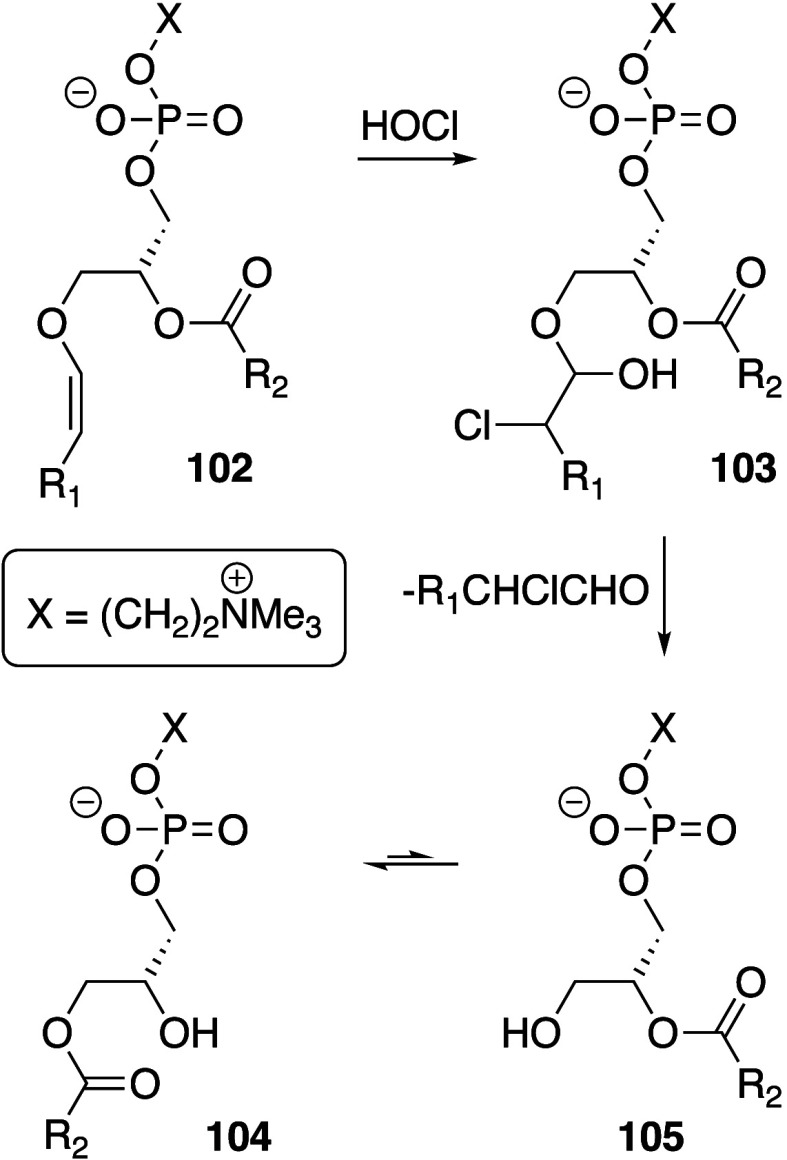
Reaction of PC Lipids with Hypochlorous Acid

Hypochlorous acid adds to double bonds *via* an electrophilic mechanism to form a mixture of 1,2-dichlorides and chlorohydrins.^467^ The latter can subsequently form epoxides by the elimination of chloride.^81^ The activity of myeloperoxidase (MPO) in cells of the immune system is primarily responsible for the formation of hypochlorous acid *in vivo*,^468^ whereas *in vitro* it is formed by the hydrochloric acid-catalyzed decomposition of hydrogen peroxide. Other hypohalous acids give similar processes. Electrophilic HOCl addition has been documented for sterols,^452^ sphingomyelin,^469^ and plasmalogens. In the latter case, the initial product (**103**) hydrolyzes to form a lyso-PC (**104**/**105**, Scheme 13).^470^ If the *sn*-2 chain is unsaturated, further HOCl addition can occur. This subsequent HOCl addition to the C=C of the lysolipid is faster than HOCl addition to the parent lipid (**102**).^471^ Ultimately, the chlorohydrin addition product with the lysolipid undergoes hydrolysis to the fatty acid and GPC.

Hypochlorous acid also forms *N*-chloro species by reaction with the amino group of PE.^81,467^

**Scheme 14:**
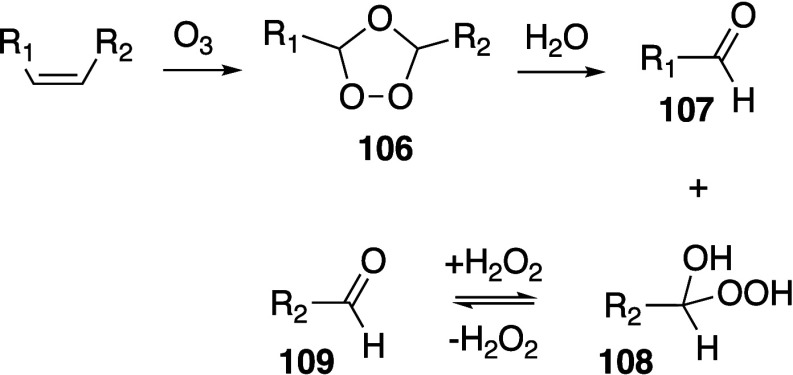
Ozonolysis Reactions of Fatty Acid Alkenes

The reaction of ozone with the unsaturated alkenes of lipid fatty acids leads, as expected, to the formation of the Criegee intermediate **106** (Scheme 14). Breakdown of **106** occurs by hydrolysis to form one equivalent of aldehyde (**107**) and one equivalent of the hydroxyhydroperoxide (**108**). The latter loses hydrogen peroxide to form a second equivalent of aldehyde (**109**). In the presence of excess ozone, aldehydes **107** and **109** can be further oxidized to the corresponding carboxylic acids.^472,473^ Good overviews of the sources of ozone^359^ and the volatile products that form *via* ozonolysis have been presented by Pöschl and Shiraiwa and Wisthaler and Weschler.^474^ Ozone is of interest because of its effects on the lungs, particularly the phospholipid-rich epithelial lining fluid (ELF), which is the first point of exposure following ozone inhalation. ELF is rich in saturated lipids, particularly DPPC, but contains a number of unsaturated PCs, including POPC, 1-palmitoyl-2-palmitoleoyl-*sn*-glycero-3-phosphocholine and 1-palmitoyl-2-linoleoyl-sn-glycero-3-phosphocholine (PLPC), alongside other lipid classes: PG, PE, PI, and plasmanyl and plasmenyl versions of PC and PE. The PG lipids are mostly monounsaturated in the *sn*-2 chain.^475^ Reaction of ozone with POPC in ELF leads to the formation of a number of oxidation products, most notably 1-palmitoyl-2-(9′-oxo-nonanoyl)-sn-glycero-3-phosphocholine (PoxnoPC) (**116**, Figure 17), which elicits a physiological response in the epithelial cells in contact with the surfactant,^475,476^ including the activation of phospholipases.^139,477^ Modern improvements in instrumentation have allowed a series of products formed by ozonolysis of the PG lipids to also be detected at ozone levels that are environmentally realistic.^475^ Interestingly, PLA_2_ recognizes the Criegee intermediate with the same efficiency as arachidonic acid,^140^ whereas the hydroxyhydroperoxide activates PLC.^139^ Aldehyde formation has also been noted following exposure of red blood cells to low levels of ozone *in vitro*,^478^ direct ozone permeation to the blood *in vivo* is unlikely because most reactions occur in the ELF.^479^ The sphingosyl alkene of sphingolipids undergoes ozonolysis to from the expected aldehyde.^480^

Singlet oxygen generation most commonly occurs trough the action of photosensitizers, in which the triplet state of the sensitizer (^3^T_1_), formed by intersystem crossing from the excited singlet state (^1^S_1_), transfers energy to ^3^O_2_. In some cases, sensitization through the singlet state can occur, although this mostly operates by ^3^O_2_ increasing the rate of intersystem crossing to ^3^T_1_.^481^ A range of organic molecules are capable of acting as photosensitizers, sharing the common property of being highly conjugated aromatic systems. In some cases, electron transfer competes with energy transfer, leading to the formation of superoxide.^482^ Dismutation of the superoxide anion generates hydrogen peroxide, which is a precursor for ^1^O_2_ through reactions with hypochlorous acid.^483^ The generation of ^1^O_2_ from the tetroxy species formed by combination of peroxyl radicals (Russell mechanism) has been described earlier in this Review. Agents that convert hydroperoxides to peroxyl radicals, including metal ions and peroxynitrite (*via* its radical decomposition products) can therefore participate in the generation of ^1^O_2_ by this route.^483^ Recent model experiments involving UVA irradiation of ethanolic solutions of fatty acids demonstrated significant generation of ^1^O_2_ by this mechanism.^484^ Recent work has demonstrated that the thermolysis of dioxetanes leads to the generation of excited triplet state ketones that transfer energy to ^3^O_2_ to form ^1^O_2_.^485^ Thermal decomposition of the endoperoxides of cyclohexa-1,4-dienes *via* a concerted pericyclic mechanism is another route that has been used to generate ^1^O_2_.^486,487^

**Scheme 15:**
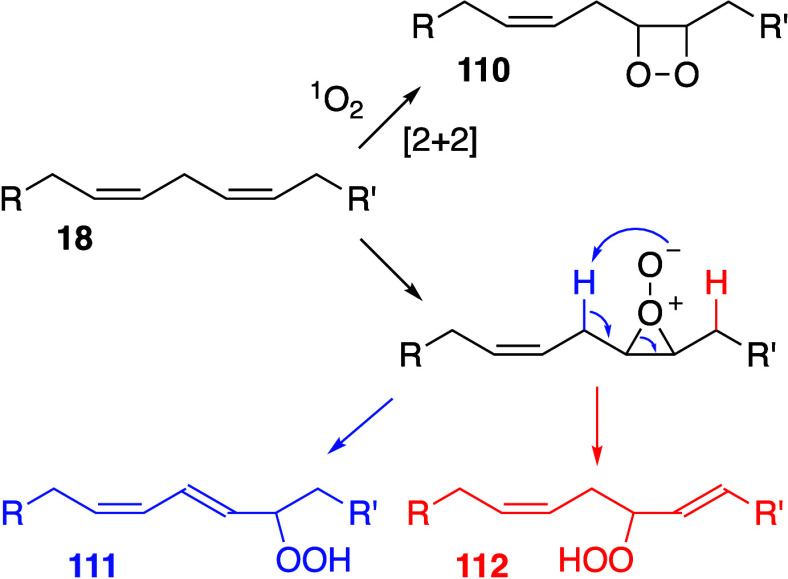
Reactions of Singlet Oxygen with Alkenes

Singlet oxygen reacts with alkenes to form dioxetanes *via* a 1,2-cycloaddition (**110**) and allyl hydroperoxides *via* the ene reaction (Scheme 15). In homoconjugated systems, the ene reaction forms both conjugated (**111**) and nonconjugated (**112**) products, with the former being the dominant product. In model reactions with linoleic acid, the ratio of conjugated to nonconjugated products is 66:34, with no preference for the alkene that reacts.^391,488^ As the first excited state of oxygen, oxidations with ^1^O_2_ are generally 3–4 orders of magnitude faster than those of ^3^O_2_ and are therefore of particular significance for monounsaturated fatty acids with low reactivity toward autoxidation by free radical mechanisms.

Cardiolipin oxidation by singlet oxygen is influenced by lipid packing. The presence of Ca^2+^ ions, for example, increases CDL’s susceptibility to oxidation by decreasing the headgroup volume of CDL and increasing the volume of the acyl chains, leading to the observation of structures with high curvature.^489^

**Figure 16:**
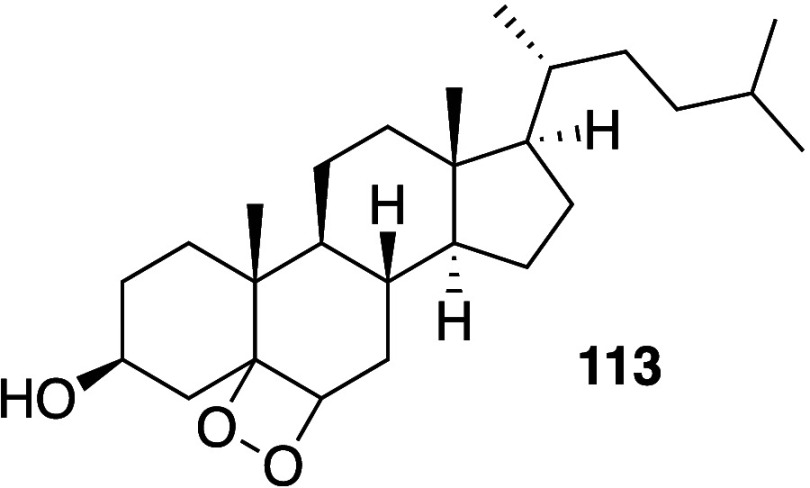
Dioxetane formed by the reaction of cholesterol with singlet oxygen.

Cholesterol reacts with singlet oxygen to form 5-hydroperoxide (**85**) and the dioxetane **113** (Figure 16). The former undergoes rearrangement to form the more stable 7-hydroperoxide, and the latter is a precursor for the formation of secosterol **92**(452) and cholesterol-5,6-epoxides. These epoxides are themselves reactive toward nucleophiles such as thiols and amines, forming 5-hydroxycholesterols substituted on the 6-position.^490^

Plasmalogens react with ^1^O_2_ to form dioxetanes that hydrolyze to form a lyso-PC and a long-chain aldehyde.^447,491^

The oxidation of sulfatides (sulfated galactocerebrosides) has been examined under ambient conditions in the presence of light. Oxidation of both sphingosyl and fatty acyl alkenes was observed over 24 h, with the former reacting slower than the latter. In the absence of light, no reaction was observed, leading the authors to conclude that the oxidation was mediated by photogenerated ^1^O_2_. In both cases, C=C bond cleavage occurred to form the corresponding aldehydes.^480^ In an earlier study, the oxidation of lactosyl and galactosyl ceramides was studied under UVA irradiation (320–400 nm). Oxidation of both sphingosyl and fatty acyl alkenes was observed alongside products resulting from oxidation in the sugar group.^492^ Oxidation in the sugar moiety is also a feature of glycated PEs, with cleavage of C–C bonds within the sugar moiety following UVA irradiation.^493^

### 4.6 Reactivity towards Oxidation of Membrane Lipids

Autoxidation reactions in membranes follow the same rate laws as their counterparts in homogeneous solution. All of the radical processes are slower in membranes, which may be attributed to the higher viscosity of the membrane (relative to solution), particularly below *T*_m_.^317^ For initiation, this leads to increased radical recombination during initiation due to cage effects and low initiator efficiency.^330^ Propagation and termination reactions of methyl linoleate at 37 °C in DMPC vesicles are approximately 10- and 100-fold slower, respectively, compared to the same processes in homogeneous solution.^330^ Slower termination reactions are also found for methyl linoleate in micelles.^329^

It is generally believed that the slower rates for propagation and termination in micelles and bilayers are a consequence of partitioning of the polar peroxyl radical centers to the interfacial region with the bulk phase.^330^ Simulations have given conflicting results, with some suggesting that while hydroperoxides may partition preferably to the membrane interface, this is not the case for the peroxyl radical, which remains within the hydrophobic core of the membrane,^494^ whereas others indicate that formation of the peroxyl radical generates more coiled conformations of the hydrocarbon chain that place the reacting carbon atoms closer to the interface.^367^ A recent study of the relative oxidation rates of a range of lipids with varying levels of unsaturation, both in solution and in bilayers, revealed that the reaction rates in bilayer are half those obtained in solution. This was attributed to reaction sites, particularly for initiation, only being accessible in the outer membrane leaflet. This study also demonstrated lipids with higher levels of unsaturation (C20:4, C22:6) oxidize faster than their less unsaturated counterparts.^495^ Interestingly, this study also demonstrated that, for highly unsaturated acyl groups, hydrogen transfer during the propagation step is an intramolecular process, with the consequence that individual acyl chains are either unmodified or highly oxidized.

Unlike the situation in solution for linoleate, in bilayers there is a preference for the formation of peroxides at C9 over C13, which may be attributed to the closer proximity of C9 to the membrane interface or to differences in availability of hydrogen atom donors at different depths within the bilayer.^317^ The rate constant for β-scission of peroxyl radicals at C9 is lower than that of C13, which may be attributed to differences in polarity at these depths in the membrane.

**Figure 17:**
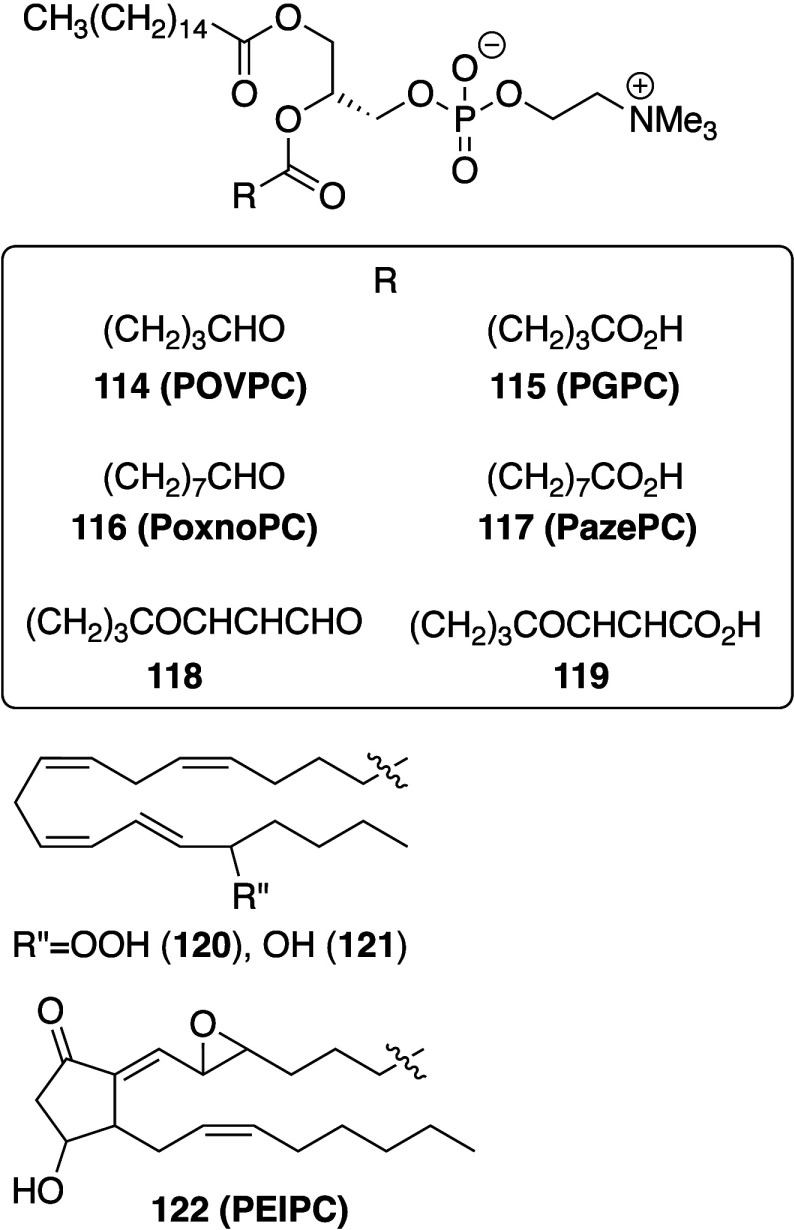
Examples of oxidized PCs.^89,499−501^ All examples here have palmitoyl at the *sn*-1 position.

For PUFAs such as arachidonate at the *sn*-2 position, reaction similarly occurs more readily *via* the bis-allyl radical center that is more proximal to the membrane surface. It is currently unclear why this is the case, but a likely reason is that this site is more proximal to the location from which water-soluble radical initiators diffuse.^74^ Unsaturated fatty acyl groups are rarely found at the *sn*-1 position of glycerophospholipids. This is the prime reason for the activation of PLA_2_ as a response to oxidative stress, as most of the acyl chain damage is on the *sn*-2 chain. For unsaturated *sn*-2 acyl chains, particularly if they are polyunsaturated, free radical initiation generally results in either the formation of hydroperoxides and their alcohol counterparts (**120**, **121**, Figure 17), the products of endoperoxide formation (**122**), or the products of acyl chain truncation, which produces species such as **114**–**119**. The range of truncated acyl groups found following lipid oxidation has been reviewed.^74,496^

Cholesterol inclusion slows the rate of oxidation.^255^ An interesting synergism exists between cholesterol and PUFA oxidation, however. Cholesterol is resistant to oxidation in saturated membranes but susceptible to oxidation in membranes containing MUFAs^497^ or PUFAs. In the latter, suppression of PUFA oxidation also suppresses cholesterol oxidation. The reason for this most likely lies in hydrogen transfer from cholesterol C7 to a PUFA peroxyl radical as a key initiation step in cholesterol oxidation.^73,452,498^ This transfer is particularly made apparent by the high tendency toward oxidation of cholesterol esters of PUFAs, where the abstraction at C7 becomes intramolecular and therefore more facile.^122^

**Figure 18:**
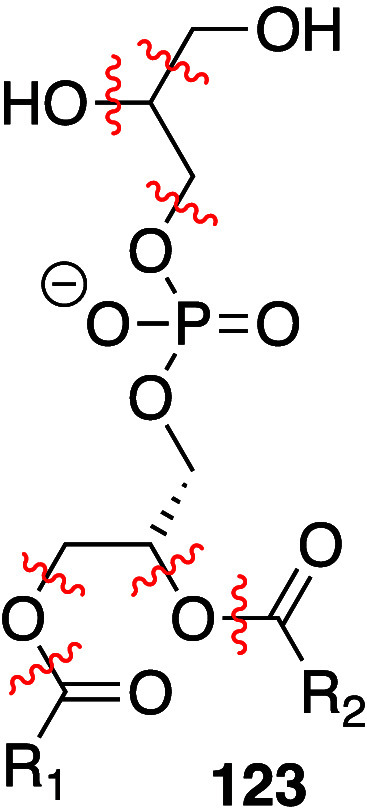
Bond cleavages, indicated by wavy lines, following γ-irradiation of DPPG (5.8 × 10^4^ Gy).

Many of the effects of γ radiation are mediated through the ROS described above. In some instances, however, unexpected bond cleavages have been found, particularly when systems composed of saturated lipids are exposed to high radiation doses. For example, irradiation of DPPC and DPPG/DPPC liposomes led to the formation of small amounts of 1,2-dipalmitoyl-*sn*-glycero-3-phosphatidic acid (DPPA) by headgroup cleavage, 1/2-palmitoyl-*sn*-propanediol-3-phosphorylcholine, 1/2-palmitoyl-*sn*-propanediol-3-phosphorylglycerol, and dipalmitoyl-*sn*-glycerol-3-phosphorylethanol, alongside lysolipids (Figure 18).^502^ These cleavages tend to occur adjacent to heteroatoms in the headgroup and glycerol sections of the lipid, which have recently been calculated to be sites most amenable to hydrogen abstraction in this part of the lipid.^503^

A recent study investigated the oxidative stability of lipids during electroformation of giant unilamellar vesicles (GUVs). Typical conditions for GUV preparation (0.3–1.2 V, 5–20 h) led to the formation of significant levels of oxidized lipids for 1,2-didocosahexaenoyl-*sn*-glycero-3-phosphocholine (DHAPC) and DLPC, whereas DOPC was significantly more stable. Ultimately, the optimum conditions were a trade-off between lipid oxidation and vesicle size and yield.^504^

The oxidative instability of lipids has been exploited to develop drug delivery systems that can be triggered using near-infrared (NIR) radiation. Luo et al. included DOPC in liposomes composed of DSPC, a porphyrin-modified lipid and cholesterol. Under NIR, the production of singlet oxidation resulted in DOPC and cholesterol oxidation and the release of entrapped doxorubicin within 1–2 min.^505^

### 4.7 Biophysical Consequences of Oxidation

A key consequence of lipid oxidation is the introduction of polar groups, including alcohols and hydroperoxides for full-length lipids and aldehydes for truncated lipids, into the normally low-dielectric hydrocarbon interior of the membrane. This presence of polar groups leads to an increase in the water penetration of the membrane.^109,506,507^ As the level of oxidized lipids becomes very high, water penetration increases to the extent that continuous pores span both leaflets of the membrane.^508−510^ The presence of cholesterol increases the pore lifetime.^508^ Simulations suggest that at levels of oxidized lipids up to 25 mol % there is little or no perturbation to headgroup conformations and lipid mixing by the presence of full length oxidized chains, but the polar groups of the chains tend to migrate toward the polar regions of the lipid interface.^373,511,512^ This presence of oxidized fatty acyl chains on the surface of the membrane has led to the development of the whisker model, in which oxidized chain ends project away from the surface of the membrane into the aqueous medium.^249,250^ The presence of whiskers is an important marker for cell recognition to trigger apoptosis or membrane recycling.^250,513^

Truncated chains, on the other hand, induce lipid disordering.^514−516^ Decreased chain ordering, lowering of phase transition temperatures, increased permeability and rates of lipid flip-flop, membrane thinning, and decreased bending rigidity have also been observed experimentally at these levels of oxidized lipids.^108,322,507,517−521^ When primary amines are present in the membrane, in lipids such as sphingosine, for example, cross-linking can occur through imine formation with reactive aldehydes, which can subsequently drive vesicle aggregation.^421^

Low molecular weight oxidized hydrocarbon fragments such 4-HNE (**29**) similarly orient themselves with the polar groups oriented toward the lipid interface. Eventually, these molecules are able to diffuse out of the membrane.^511,512^ The enhanced water penetration, and the partitioning of polar groups of oxidation products toward the interface lead to an increase in the area per lipid. Oxidized lipids have also been found to desorb from the membrane.^512,522^ In contrast to the products of oxidation, some simulations indicate that the intermediate peroxyl radical groups move toward the lipid interface,^367^ whereas others suggest they may remain more deeply buried.^494^

In three-component mixtures containing cholesterol and sphingomyelin that exhibit microphase separation into a sphingomyelin-rich liquid-ordered (L_o_) phase, the presence of oxidatively truncated lipids has been observed to stabilize L_o_ domains in a both monolayer^523^ and bilayer^507^ models.

Hydroxyl radicals, produced by the Fenton reaction, have been observed to lead to the formation of more condensed phases in PC monolayers and bilayers. This increased chain ordering has been attributed to loss of the choline ammonium group after reaction with the hydroxyl radical, which forms a negatively charged lipid that can coordinate Fe(II) ions, leading to cross-linking.^515,524^

### 4.8 Biological Consequences of Lipid Oxidation

Oxidative damage to lipids and sterols is associated with numerous diseases, particularly in conditions of oxidative stress, where the normal balance between the formation of reactive oxygen and nitrogen species and their removal by cellular defense mechanisms is perturbed in favor of the former.^525,526^ In many of these diseases, oxidized lipids arising from both enzymatic and nonenzymatic oxidation are typically present. Nonenzymatic processes include the reactions described in the previous sections, but it is worth noting that in some cases linked to disease the oxidative processes occurred during food preparation or storage, with subsequent dietary uptake of the oxidized products.^423,527,528^ Enzymatic processes include ferroptosis and reactions catalyzed by lipoxygenases, cyclooxygenases, and cytochrome P450. Lipoxygenases and cyclooxygenases act on fatty acids released from lipids by the action of phospholipases.^529^ Ferroptosis is a key enzyme-mediated process for cell death and can be triggered, among other methods, by elevated levels of lipid peroxides in cells that are under reactive oxygen stress. Ferroptosis involves an iron-mediated oxidation of lipids that leads to plasma membrane rupture, mitochondrial membrane breakdown, and ultimately cell unviability.^321,530−535^ Lipoxygenases selectively form hydroperoxides of PUFAs as precursors for bioactive fatty acid mediators such as leukotrienes and lipoxins. Cyclooxygenases (prostaglandin-endoperoxide synthases) catalyze the formation of secondary oxidation products such as endoperoxides and their subsequent transformation into prostaglandins.^356^ Cytochrome P450 enzymes constitute a family of monooxygenases that oxidize hydrophobic materials selectively to form alcohols or epoxides. In the case of sterols, sites of oxidation notably include the hydrocarbon chain (e.g., at C24). For lipids, notable substrates are PUFAs, to form lipoxins, and leukotrienes. Many of the oxidized lipids and secondary fatty acid oxidation products formed as a consequence of the activity of these enzymes are involved in the inflammatory response, some with pro-inflammatory effects and others with anti-inflammatory effects depending on the route and timing of their formation.^529,536^ Thus, several of the oxidized lipids presented in section 4.1.3 that are generated by nonenzymatic methods have a biological activity themselves; others can serve as substrates for additional transformation by enzymes. CYP is also involved in an overproduction of reactive oxygen species in mitochondria, associated with disease conditions where normal homeostasis has been disrupted.^537,538^

Given the potential molecular diversity offered by both enzymatic and nonenzymatic oxidation of the natural lipidome, it is unsurprising that the potential scale of the oxidatively modified lipidome, the epilipidome, is vast, with up to 10^6^ different lipids with an oxidation product in a single chain and 10^12^ lipids with an oxidation product in both chains. Many members of the epilipidome are involved in cellular responses such as inflammation, apoptosis, and the triggering of the innate immune response.^315,539^

Oxidized lipids and sterols are particularly associated with a number of diseases, including cardiovascular diseases, most notably atherosclerosis,^126,321,382,453,540−542^ nonalcoholic fatty liver disease,^382^ lysosomal storage disorders,^461^ and Alzheimer’s disease.^321,543−545^ In general, the presence of oxidized lipids can lead to biological consequences either through changes to the material properties of the membrane or through a direct biological activity of the oxidized lipids as enzyme substrates or receptor ligands. Examples of the effects of oxidized lipids on membrane structure include the release of cytochrome c from mitochondrial membranes following oxidative damage by CYP activity^538^ and the loss of membrane integrity seen during ferroptosis. The presence of oxidized lipids within the membrane can also influence the activity of membrane-embedded receptors, as evidenced by the increased activity of the human serotonin 1A receptor in the presence of oxidized lipids such as PoxnoPC (**116**).^546^ In some cases, membrane remodeling, mediated by desaturase enzymes, has been observed in response to an oxidative challenge, suggesting that cells are able to adapt their lipidome in order to maintain membrane function.^547^

Useful indicators for the presence of fatty acid oxidation arising by autoxidation, as opposed to enzymatic methods, can be obtained by examination of the stereochemistry of the hydroperoxide and hydroxyl products and the balance between (*E*,*E*)- and (*Z*,*E*)-isomers for di-unsaturated fatty acids. Oxidation by lipoxygenases favors the (*S*)-isomer of hydroperoxides and their corresponding alcohols, whereas autoxidation produces a racemic mixture. The ratios of (*E*,*E*)- and (*Z*,*E*)-isomers of hydroxylinoleate varies subtly between controls and samples under oxidative stress. In controls and samples subjected to lipoxygenase treatment and samples exposed to singlet oxygen, the (*Z*,*E*)-isomers predominate; in samples under stress by autoxidation, or chemical oxidation by hypochlorite, the ratio of (*E*,*E*)- to (*Z*,*E*)-isomers is closer to 1:1.^382^ Tracking these indicators through the progress of a disease can provide useful information on the relative importance of environmental stresses and enzymatic oxidation. For example, in the early stages of atherosclerosis, the *S*/*R* enantiomer ratio of 13-hydroxylinoleate is high, while in the later stages it tends toward 1:1, indicating the activity of lipoxygenases in the early stages, and increases the level of in 13-hydroxylinoleate have also been found to be higher in plaques than in plasma.^382^ Altered levels of these lipid biomarkers have been associated with a number of other diseases, including nonalcoholic fatty liver disease and glaucoma.^382^

Oxidized lipids have been associated with binding to a number of receptors, including G-protein coupled receptors associated with the inflammatory response (platelet-activating factor receptor), nuclear receptors associated with lipid metabolism (peroxisome proliferator-activated receptors, PPARα), and receptors associated with innate immunity (CD14 and Toll-like receptors).^536^ Lipids derived from PUFAs are capable of effecting vascular relaxation, L-type calcium channel activity, and glucose homeostasis.^356^ Truncated PCs, such as 1-palmitoyl-2-(5-oxovaleroyl)-*sn*-glycero-3-phosphocholine (POVPC) (**114**), are able to mediate the binding of oxidized low density lipoprotein to receptors involved in low density lipoprotein internalization and have a key role in the formation of foam cells and the progression of atherosclerosis.^126^ Oxysterols have been found to induce nitric oxide (NO) production and the release of pro-inflammatory cytokines.^548^

Reactive carbonyl species such as 4-hydroxynonenal (4-HNE, **29**) and 4-hydroxyhexenal are sufficiently long-lived and amphiphilic to enter general circulation during food digestion or following their formation in tissues. Elevated blood plasma levels of 4-HNE are associated with Alzheimer’s disease, some cardiovascular diseases, and rheumatoid arthritis. The effects of 4-HNE are concentration- and cell-type-dependent, with effects on cell differentiation at low concentrations and cell apoptosis or necrosis at high concentrations.^549^ 4-Hydroxyhexenal is also markedly cytotoxic^537^ and mutagenic as a consequence of adduct formation with the imidazole ring of guanine. Furthermore, epoxides formed from these unsaturated aldehydes are also reactive toward DNA bases.^550^ Malondialdehyde (**53**) levels have been found to be higher in other diseases, including Alzheimer’s disease, diabetes, chronic obstructive pulmonary disorder, and cirrhosis.^382^ Glycated lipids have been shown to have cellular toxicity and are found at elevated levels in patients with diabetes. Cell integrity can also be negatively impacted by their accumulation.^422^ Secosterol aldehydes (**92** and **93**) have been detected in the brains of patients with Alzheimer’s disease or Lewy body disease and in atherosclerotic tissues. In all of these cases, secosterol aldehyde-modified proteins are detectable.^453^ Protein derivatization by lipid-derived reactive carbonyl species has been shown to affect protein–protein, protein–DNA, and protein–membrane interactions, as well as the cellular location of the modified molecule. Protein lipoxidation is site- and protein-selective. The predominant sites for modification are the side chains of cysteine and lysine, and to a lesser extent, histidine. Notable targets include albumin (circulation), chaperones, and some cytoskeletal proteins (tubulin, actin, and vimentin). In some cases, such as Ras, lipoxidation activates the receptor to trigger a secondary messenger cascade.^403,526^

**Scheme 16:**
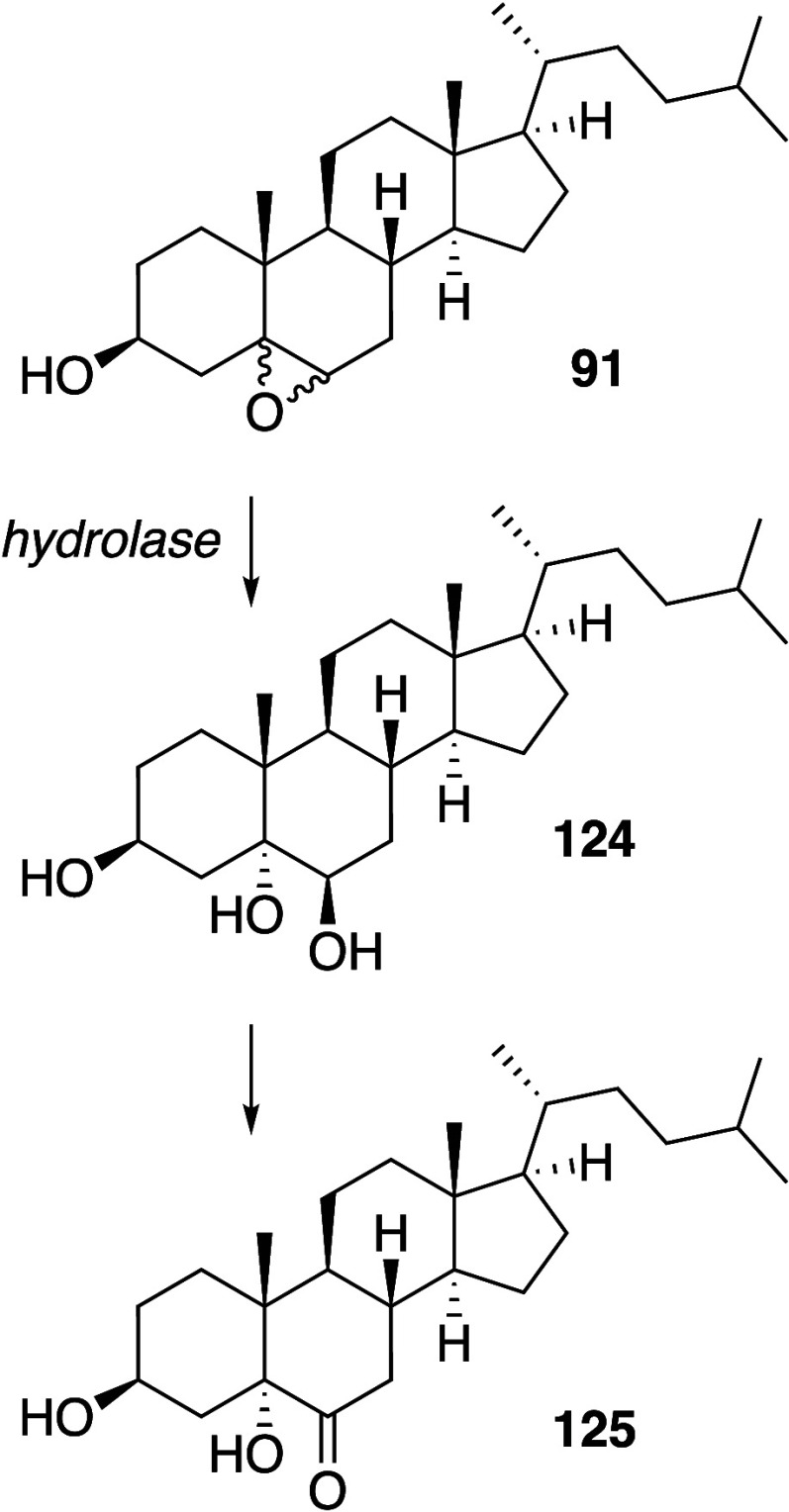
Sterols Formed by Enzymatic Processing of 5,6-Epoxycholesterol

In the case of cholesterol, the 5,6-epoxy derivatives such as **91** (Scheme 16) are able to serve as substrates for cholesterol epoxide hydrolase that stereoselectively forms the corresponding 5α,6β-diol (**124**), which is subsequently transformed by with catalysis by a dehydrogenase into ketone **125**.^461^ The 7β-isomer of hydroxycholesterol (**82**) is also a substrate for a dehydrogenase to form the corresponding 7-oxo compound (**83**).

Compound **125** is found in elevated levels in breast cancer tissue, and high levels of **125** and **83** are associated with several types of Niemann–Pick disease. These latter two sterols are also precursors for oxidation by CYP to form ligands for oncoproteins in the hedgehog pathway.^454,461,551^ All of the sterols in Scheme 16 are found at elevated levels in atherosclerotic plaques relative to blood plasma. Raised levels of the 7β-isomer of hydroxycholesterol (**82**) are a notable marker for several cardiovascular diseases.^454,537,552^ Some oxidized sterols, notably 7-ketocholesterol (**82**) in the context of autoxidation, are inverse agonists of retinoid-related orphan receptors, are able to bind to cytoplasmic transport proteins, and are able to up-regulate enzymes involved in cholesterol biosynthesis.^537^ 24(*S*)-Hydroxycholesterol (24S-OHC) and 25-hydroxycholesterol (25-OHC) are acylated by an Acyl-CoA:cholesterol acyltransferase 1 (ACAT1) and are then incorporated into lipid droplet-like structures that are implicated in a process leading to cell death in some cell types.^553^

### 4.9 Future Directions

Significant advances have been made in characterizing the vast range of products resulting from lipid oxidation, including oxidized lipid and fatty acid fragments and biological molecules modified by these fragments. However, there remain some significant obstacles to understanding the links between oxidative stress and disease. The complexity of the product profile under oxidative stress makes it challenging to determine which products have significant biological activity of their own and which are relatively inert oxidation byproducts.^133,475,544^ While it may be possible to correlate complex oxidation profiles to particular diseases, moving from demonstrating correlations to establishing molecular mechanisms is difficult. As evidenced by the recent strides in understanding ferroptosis, in the biological context the formation of oxidized lipids can trigger processes that significantly change the profile of oxidation products. It is now established that some oxidized lipids regulate inflammation and have particular effects on macrophages, inducing behavior characterized by reduced phagocytotic capability and reduced motility.^126^ It is likely that other macrophage phenotypes linked to disease are induced by particular lipid oxidation products. Establishing which oxidation products produce particular patterns of immune cell activity, and establishing the molecular mechanisms underpinning this activity, will be vital. Understanding the roles of oxidation products from cardiolipins will also be crucial. Given their proximity to cellular sites of ROS generation in mitochondria and their susceptibility to oxidation, cardiolipins are capable of yielding a vast array of oxidized products, several derivatives of which are active in regulating the inflammatory response and apoptosis.^444,496^

For applications that do not involve living systems with active antioxidant processes, there remain ongoing challenges to improve stability and thereby shelf life. In the case of formulations with liposomes for drug delivery applications, particular obstacles to development are presented by excipients and active components that are either prone to oxidation themselves or increase the sensitivity of lipids to oxidation.^257,381^ Currently, our understanding of how these types of molecules affect stability toward oxidation (and ester lysis) is insufficient to predict their effects *de novo*. In food technology there is a need to better relate the formation of glycated products to stages in food processing and attain a better understanding of mechanisms by which glycation occurs, as the product profiles can vary significantly with changes in temperature and pressure.^422^

From an analytical standpoint, challenges remain in identifying some oxidation products for which the fragmentation pathways are not well-known. It is also hard to determine the location of a chain at the glycerol *sn*-1 or *sn*-2 position if it is oxidized. Part of the solution to these issues will involve improvements in software.^539^ Mechanistically, there is a desire to clarify which hydroperoxides arise by direct oxidation, and which by isomerization,^417^ and the extent to which each of these mechanisms operates *in vivo*.

## 5 Isomerization

**Scheme 17:**
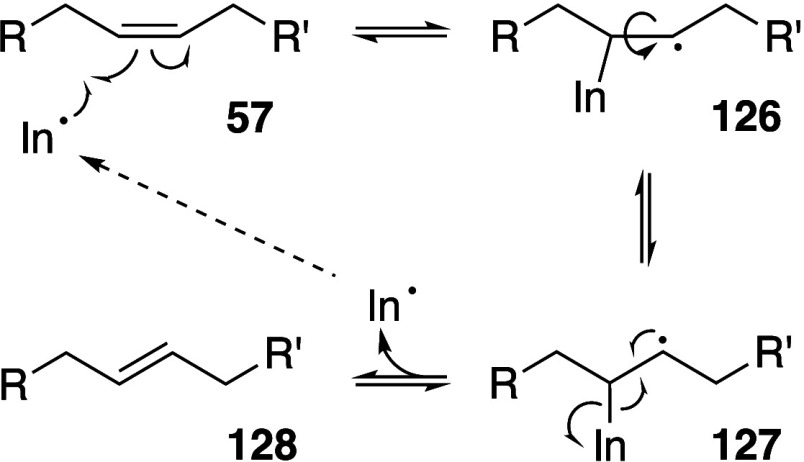
Alkene Isomerization Mediated by Electrophilic Radical Addition

In addition to the free radical reactions that lead to autoxidation, free radical addition of sulfur- and nitrogen-centered electrophilic radicals forms adducts that, if not trapped by hydrogen abstraction, subsequently eliminate the electrophilic radical by β-scission. These radical addition–elimination reactions proceed with isomerization of the alkene from *Z* to *E* and formally represent the propagation step of a radical reaction (Scheme 17), with formation of the electrophilic radical as the initiation step and dimerization of the electrophilic radical as the termination step. These reactions have been well reviewed by Chatgilialoglu et al.^554^

### 5.1 Sulfur Radicals

**Figure 19:**
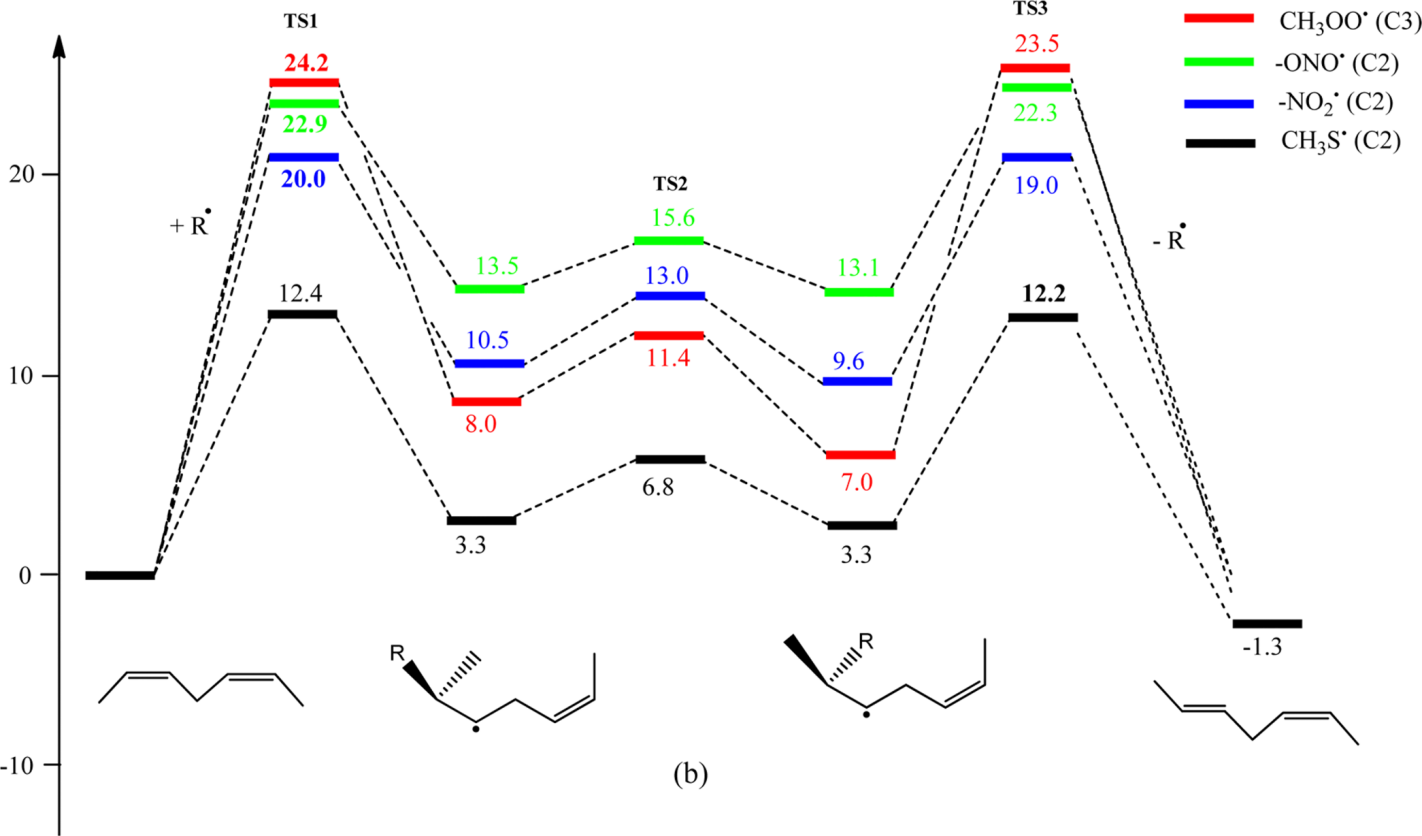
Free energy landscapes of radical additions. Only the lowest barrier path is shown; the reaction sites of lowest barrier are shown in parentheses. From reference (557). Copyright 2014 American Chemical Society.

Thiols are a significant source of electrophilic radicals, forming thiyl radicals *via* single electron oxidation. Initiation is typically mediated by γ-radiation, azo initiators, hydrogen transfer to an allylic radical, hydrogen transfer to a photochemically excited ketone, or electron transfer from a reducing metal ion. Model reactions using 2-mercaptoethanol with methyl oleate have enabled the rate constants for each of the steps in Scheme 17 to be determined.^555,556^ At equilibrium, all MUFAs comprise an *E*/*Z* ratio of 84:16. Rate constants for thiyl radical addition to (*Z*)- and (*E*)-alkenes (**57** and **128**) are broadly similar (1.6 × 10^5^ and 2.9 × 10^5^ M^–1^ s^–1^ respectively), but the fragmentation of the alkyl radical **127** occurs with a rate constant that is an order of magnitude greater than that of **126** (1.6 × 10^8^ and 1.7 × 10^7^ s^–1^). This rate difference is attributed to the transition state for the elimination of the thiyl radical from **127** being lower in energy than that for elimination from **126**. For but-2-ene, this energy difference has been calculated as 1 kcal mol^–1^, attributed to a late transition state where steric interactions between the alkene substituents are more significant during the elimination from **126**.^555^ Similar small differences in the transition state free energy barriers for elimination from the intermediate radical have been suggested by theoretical studies of the reaction of CH_3_S^•^ with homoconjugated dienes. The transition state free energy for the decomposition to the (*E*,*Z*)-alkene of the radical intermediate (corresponding to **126**), formed by the addition of CH_3_S^•^ to C2 of (2*Z*,5*Z*)-hepta-2,5-diene, was lower in energy by 0.2 kcal mol^–1^ than the transition state for (*Z*,*Z*)-alkene formation (Figure 19).^557^ Addition of CH_3_S^•^ to C2 had a lower energy barrier to addition than C3. The results were similar for linoleic acid, with a transition state energy difference for elimination to *E*,*Z* vs *Z*,*Z* of 0.1 kcal mol^–1^. Reactions of thiyl radicals with PUFAs are more complex because hydrogen abstraction to form bis-allyl radicals competes with radical addition, with the ratio of the two reactions being approximately 1:1. Isomerization following radical addition proceeds in a stepwise manner, forming mixtures with combinations of (*E*)- and (*Z*)-alkenes at each position.^554^ The critical free energy barrier for thiyl radical addition has been calculated to be lower than that for hydrogen abstraction by 1.3 kcal mol^–1^.^557^ Recent modeling studies indicate that the radical addition step has a low energy barrier due to stabilizing interactions between the unpaired electrons of the sulfur and the π* orbitals of the alkene.^558^

**Scheme 18:**
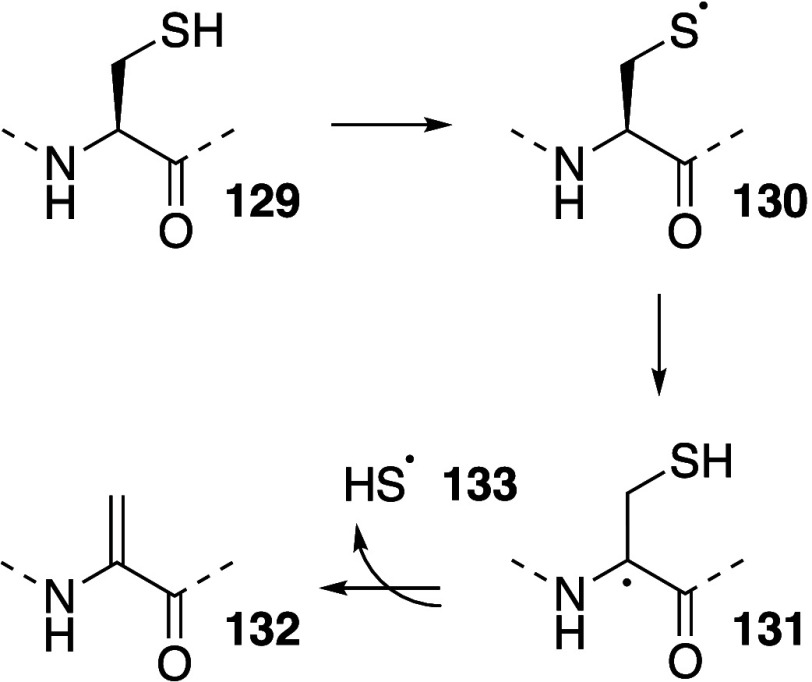
Generation of the Sulfhydryl Radical from Cys

Cysteine, methionine and glutathione are potential sources of sulfur-centered radicals *in vivo*.^559,560^ Cysteine has a low abundance in the transmembrane regions of proteins, and it has been suggested that its presence may act to accelerate propagation,^384^ noting that lipophilic thiols generally accelerate peroxidation and isomerization reactions.^561^ Aside from thiyl radical formation by hydrogen transfer to allylic radicals, pulse radiolysis experiments with Cys suggest that the intermediate thiyl radical (**130**, Scheme 18) undergoes 1,2- and 1,3-hydrogen shifts.^562,563^ In the latter case, subsequent β-scission generates dehydroalanine (**132**) and a sulfhydryl radical (**133**). Sulfhydryl radicals have been shown to induce *E*/*Z* isomerization in liposomes.^564^ Thiyl radicals generated from penicillamine and cysteamine undergo similar hydrogen migrations, although the former is restricted to 1,3- and the latter to 1,2-migration.^565^ Carbon centered radicals can also be formed at various sites in peptides by hydrogen transfer reactions with the thiyl radical formed from glutathione.^562^ These hydrogen transfer reactions occur under physiological conditions and are tolerant of oxygen.^560^ Single electron reduction of disulfides *via* γ-irradiation provides another route to thiyl radicals.^565,566^ For example, dimethydisulfide-mediated isomerization of the oleoyl group in POPC liposomes leads to >90% of the *trans* isomer at a γ-irradiation dose of 126 Gy. Carbon dioxide radical anions, produced by photodegradation of citrate in the presence of Fe(II), are able to react with disulfides to form thiyl radicals that can induce fatty acid isomerization in a synthetic surfactant, although this has not yet been demonstrated for a protein disulfide in membranes.^567^

Thioethers such as methionine can potentially act as hydrogen atom acceptors to form thiouranyl radicals (R–S^•^(H)–R′) that subsequently fragment to a thiol (methyl mercaptan in the case of methionine) and a carbon-centered radical. The thiol is then available for subsequent thiyl radical formation.^554^

There is some evidence for thiyl radical-mediated isomerization *in vivo*, although the levels of (*E*)-isomers that accumulate are small unless conditions are used that favor the formation of thiyl radicals. Incubation of human monocytic leukemia cell membranes with thiols (2-mercaptoethanol, glutathione, and the biologically active thiol 3-(2-mercapthoethyl)quinazolin-2,4(1*H*,3*H*)-dione (MECH)) produced small increases in the levels of (*E*)-alkenes, typically 1–2% of total fatty acids, for 2-mercaptoethanol and glutathione but not for MECH.^568^ Increases were most notable for PUFAs, with little change in the *E*/*Z* ratio of MUFAs. Similar results were obtained for normal and cells cultured under oxidative stress. On the other hand, incubation of NTera-2 testicular cancer cells with bleomycin (BLM) led to significant increases the in proportion of both saturated fatty acids and (*E*)-isomers of MUFAs and PUFAs.^569^ The respective proportions of MUFAs and PUFAs with at least one (*E*)-alkene (% of total FA) in these experiments were 2.9% and 0.38% compared to 0.14% and 0.05% in controls without BLM, respectively.

**Scheme 19:**
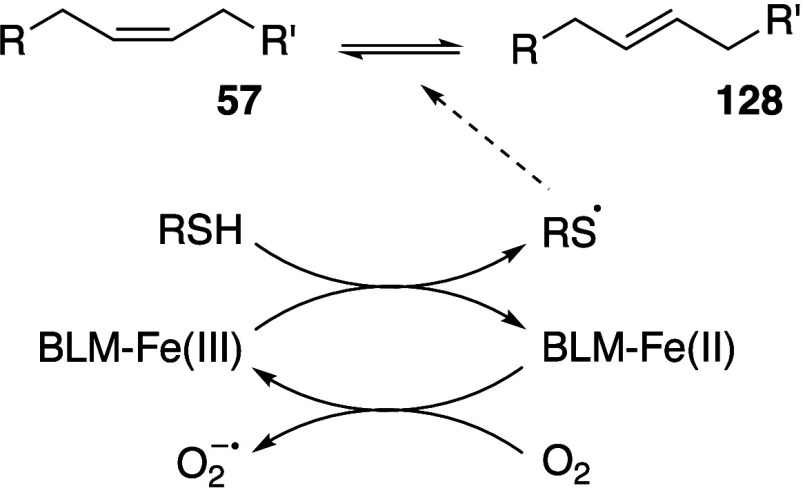
Generation of Thiyl Radicals by Fe(III) Complexes of Bleomycin (BLM)

Thiyl radical-induced isomerization produced by BLM, in combination with iron, has been well studied. Oxidation of the Fe(II) complex with BLM in the presence of O_2_ leads to the formation of the BLM–Fe(III) complex and ROS, most notably superoxide.^570,571^ The production of ROS in the BLM–iron system is associated with lipid peroxidation.^572^ In the presence of thiols, reduction of BLM–Fe(III) to BLM–Fe(II) occurs with concomitant formation of thiyl radicals (Scheme 19). In model POPC liposomes, *E*/*Z* isomerization of the oleoyl group to elaidoyl, promoted by BLM–Fe(II) complexes in the presence of oxygen and an amphiphilic thiol, occurs without the formation of significant oxidation products. Isomerization yields are typically 25–30% *E* after 24 h at 5% oxygen, with the amount of (*E*)-isomer decreasing at higher oxygen levels. In more complex models, *E*/*Z* isomerization of both MUFAs and PUFAs occurs, but the predominant products arise by PUFA oxidation.^573^

Sulfonyl radicals (RSO_2_^•^) form by rearrangement of thioperoxyl radicals (RSOO^•^), which themselves form reversibly through the reaction of thiyl radicals with oxygen, although the equilibrium favors the thiyl radical in all but saturating oxygen conditions. As a consequence, sulfonyl radical reactivity is only likely to be significant in exceptional circumstances, but these radicals nevertheless produce similar *E*/*Z* ratios to thiyl radicals by the general process outlined in Scheme 17.^554^

### 5.2 Nitrogen Radicals

Isomerization of isolated alkenes by NO_2_^•^ has been demonstrated in the gas phase, where radical addition competes with allylic hydrogen abstraction, but generally NO_2_^•^ reactivity with these alkenes is low.^554^ In PUFAs, hydrogen abstraction to form allylic radicals predominates over NO_2_^•^ addition in the absence of oxygen and water, with ∼5% isomerization (vs addition) products at low concentrations of NO_2_ (6.8 ppm), which increases to ∼40% at higher NO_2_ levels (81 ppm). In aqueous or aerated systems at these NO_2_ levels, no isomerization products are detectable.^574^ This contrasts with *ab initio* predictions that addition reactions through the nitrogen should be favored in polar media, although the difference in the free energy barriers for hydrogen abstraction and free radical addition is small.^557^ The involvement of NO_2_^•^ in isomerization reactions *in vivo* has been questioned.^575^ Reduction of NO_2_^•^ radicals by thiols such as glutathione to generate thiyl radicals is potentially a more important mechanism for producing isomerization *in vivo*.

### 5.3 Isomerization of Membrane Lipids

Isomerization reactions of membrane lipids with thiyl radicals are tolerant to oxygen (up to 0.2 mM) and follow the same trends in reactivity as model reactions in homogeneous solutions, with each double bond of PUFAs behaving independently irrespective of chain position. Differences are seen for reactions in micelles and liposomes that depend on the ability of the parent thiol to penetrate the membrane, with lipophilic or amphiphilic thiols such as 2-mercaptoethanol being much more effective at producing isomerization than polar thiols like cysteine.^554^ With amphiphilic thiols, double bonds closer to the membrane interface isomerize ahead of those buried deeper within the membrane, reflecting the increased encounter rate between the corresponding thiyl radicals and surface-proximal alkenes.^573,576^

### 5.4 Biophysical Effects of Isomerization

Because of the detrimental effects of dietary “trans fats”, particularly in relation to cardiovascular disease, the effects of the (*E*)-alkenes on the biophysical properties of the membrane have been well studied. In general, for membranes containing monounsaturated fatty acyl groups, replacing the (*Z*)-alkene with an (*E*)-alkene leads to properties that are intermediate between the (*Z*)-alkene and corresponding fully saturated systems.^577^ Examples are given in Table 3.

**Table 3:** Selected Properties of Lipids Containing (*E*)- and (*Z*)-Alkenes

lipid	configuration	*T*_m_ (°C)^a^	Δ*H*_m_ (kJ mol^–1^)^a^	–*S*_CD_^d^
POPC	9*Z* (*sn*-2)	–3.4–2.4	19.7–32.9	0.091 (46 °C)
PEPC	9*E* (*sn*-2)	26^b^, 35^c^		0.156 (46 °C)
PSPC		48.3–52	35.7–43.1	
SOPC	9*Z* (*sn*-2)	–0.1–8.3	27.5–28.5	
SEPC	9*E* (*sn*-2)	26	35.1	
DSPC		54.3–58.2	38.1–45.4	0.173 (65 °C)^e^
DOPC	9*Z* (*sn*-1/2)	–24–21	31.8–46.9	0.098 (51 °C)
DEPC	9*E* (*sn*-1/2)	12^f^	33.1^f^	0.13 (57 °C)

a The range of values, in water, presented in ref (8) unless indicated otherwise.

b From ref (578).

c From ref (579).

d C–D bond order parameters for the *sn*-2 chain deuterated at position 9. All data from ref (8).

e Perdeuterated in both chains.

f In 0.1 M NaCl, 50 mM Tris, 10 mM EDTA (pH 7.4).

Other properties of membranes containing (*E*)-isomers of fatty acids are consistent with tighter lipid packing compared to the corresponding (*Z*)-isomers, including decreases in fluidity,^580^ lateral^581,582^ and transverse mobility (“flip-flop”),^580^ and permeability.^580,583^

Binary mixtures containing lipids with (*E*)-fatty acids exhibit phase behavior that is broadly similar to their (*Z*)-counterparts, but with subtle differences resulting from the higher *T*_m_ of (*E*)-fatty acids and the affinity of (*E*)-fatty acyl groups for cholesterol. For example, as with DOPC, binary mixtures of 1,2-dielaidoyl-*sn*-glycero-3-phosphocholine (DEPC) with DPPC have a miscibility gap, with coexisting gel and fluid phases between 20 and 70 mol % DPPC at room temperature.^584,585^ As the *T*_m_ of DEPC is 12 °C, DEPC/DPPC binary mixtures are always in the gel phase below this temperature, regardless of composition, whereas at this temperature DOPC/DPPC mixtures, for example, are in the gel phase at >90 mol % DPPC, the fluid phase at <20 mol % DPPC, and in a mixed gel/fluid phase at compositions in between. Binary DEPC/DPPE mixtures exhibit fluid–fluid immiscibility.^585^ Cholesterol has a higher affinity for PCs with (*E*)-fatty acids than their (*Z*)-counterparts, as demonstrated by cholesterol partitioning experiments. The *E*/*Z* difference in cholesterol affinity becomes more pronounced with increasing chain length.^583^ Ternary POPC/1-palmitoyl-2-elaidoyl-*sn*-glycero-3-phosphocholine (PEPC)/cholesterol (60:30:10) membranes exhibit properties that are consistent with the formation of liquid ordered domains enriched in cholesterol and PEPC. These domains melt at a lower temperature than the corresponding system in which PEPC is replaced with 1-palmitoyl-2-stearoyl-*sn*-glycero-3-phosphocholine (PSPC).^586^

Modeling experiments have suggested that the conformational and packing behaviors of (*E*)-fatty acyl chains closely resemble those of saturated acyl chains.^582,587^ This has led to predictions that (*E*)-FFAs should increase membrane rigidity in a similar manner to palmitic acid.^588^ It has also recently been suggested that fatty acids with a (*E*)-alkene exhibit greater conformational flexibility at the C–C bonds adjacent to alkene than their (*E*)-counterparts. The increased flexibility allows for better packing, accounting for the increased order and affinity for cholesterol of (*Z*)-fatty acids.^589^

### 5.5 Future Directions

While many of the chemical details of the isomerization process are well described, there is still much to learn considering the propensity of individual fatty acids to undergo isomerization in the context of a complex biological membranes and the effects of (*E*)-fatty acid generation on membrane protein activity, particularly as the levels of (*E*)-fatty acid isomers increase with aging.^590^ Whether the presence of particular endogenous (*E*)-isomers, or combinations of (*E*)-isomers, are biomarkers for specific conditions is still not well characterized.^591^

## 6 Concluding Remarks

### 6.1 The Biological and Biophysical Effects of Chemical Reactivity

The products of membrane lipid reactivity have significant, and usually detrimental, effects on membrane integrity. Lysolipids cause perturbations to the bending rigidity and the morphology of lipid membranes and increase bilayer permeability. Oxidation of fatty acyl chains introduces polar groups into previously apolar regions of the lipid. While in some cases these polar groups migrate to more polar interfacial regions, the general effect of their presence is to disrupt bilayer integrity and allow water penetration to greater depths. As a consequence, the barrier to the diffusion of polar molecules across the membrane is reduced and the transbilayer relocation of lipids can occur more easily. This oxidative damage has a particular chance to accumulate when tissue turnover is reduced, which occurs notably in the deposits associated with cardiovascular disease and amyloid diseases. Several of the oxidized fragments resulting from fatty acid oxidation are able to modify other molecules, notably proteins. In some cases this can lead to loss of function. The distribution and diversity of protein modifications by electrophiles, the “adductome”, is complex and likely to be characteristic for particular diseases.^404^ The sensitivity of mass spectrometric techniques, exemplified by the work of He et al.,^441^ also revealed a similar complexity for lipid modifications by glycation. While the levels of these modifications may not have a significant physiological effect, they may offer up the potential for early detection of disease conditions. Similar complexity is also found in the “oxylipidome” of PUFAs, including their secondary oxidation products.^388,536^

It will be interesting to see whether modern advances in artificial intelligence can be used to detect trends in the distributions of lipoxidated proteins, fatty acid oxidation products, and glycated lipids for the detection of disease conditions at very early stages. Such detection would enable early interventions in these diseases.

### 6.2 The Time Scales of Lipid Chemical Reactivity

With respect to the consequences of lipid reactivity *in vivo*, it is worthwhile to consider the relative rates of some of the relevant processes from the perspective of both the reactions themselves and the typical rates of turnover for lipids and proteins. While the details vary significantly according to cell (and stage of the cell cycle, as well as subcellular location), tissue, and organism, there are some general trends in protein and lipid turnover. Protein half-lives vary from minutes to thousands of hours. Higher turnover rates are found in cells with short cell cycles or those that are dividing. In mice for example, proteins in the liver and brain have average half-lives in the range of 3–9 days. Some proteins are very long-lived (years or the entire lifespan). These long-lived proteins include proteins in postmitotic cells such as crystallins and aquaporins of lens fiber cells, but also some proteins in active cells such as components of the nuclear pore complex, where the half-life can be over a year.^283,592^ Lipids have half-lives that tend to be approximately 30% shorter than proteins.^593^ In many mammalian cells, degradation of approximately half of the total phospholipid occurs every one or two cell divisions,^594^ although as with proteins lipids in some postmitotic cells are not turned over during the entire lifespan of the organism.^595^ In eurkaryotic cells, there is a continuous transport of lipids from their site of biogenesis (ER for glycerophospholipids, peroxisomes for plasmalogens) to their site of activity and then onward to their site of degradation (often in lysosomes). Recent studies have highlighted the key roles of membrane contact sites, which permit the movement of lipids between organelles, as key mediators of lipid synthesis and degradation.^596^

Overall, the continuous turnover and trafficking of lipids occurs on a similar time scale to lipid hydrolysis (Section 2) in the absence of enzymes, with a half-life of hours to days. Lipidation reactions (Section 3), which also generate lysolipids, are typically marginally faster than hydrolysis reactions. Cellular lysolipid levels are under tight regulation, and in addition to recycling by degradation, lysolipids are also recycled by reacylation to the lipid under enzyme control.^597^ Consequently, under normal circumstances in a healthy cell, the products of hydrolysis should not accumulate. However, in circumstances where hydrolysis is accelerated, or turnover is restricted, lysolipid levels may increase to become physiologically significant. A good example occurs during the inflammatory response, where the generation of lysolipids is accelerated by PLA_2_. Drugs that reduce lysolipid processing, accelerate lipid hydrolysis, or undergo lipidation reactions may increase lysolipid levels sufficiently to trigger a physiological response. It is notable in this context that some of the drugs known to cause drug-induced phospholipidosis (DIPL), a lysosomal storage disorder characterized by the accumulation of lipids in multilamellar arrays, are also known to promote lysolipid formation.^272,598^ Although a direct causal link between lipid hydrolysis and DIPL has not been established, the molecular mechanisms behind DIPL are still the subject of debate.

At face value, many of the key steps in lipid oxidation (Section 4) occur with rates that are significantly higher than lipid turnover, and consequently one might expect that the levels of lipid oxidation products would be continuously high. However, there are two key points to note. First, many of the experiments to determine the mechanistic details of oxidation are conducted under forcing conditions, with levels of oxidants far higher than those that occur in most normal circumstances.^599,600^ Second, oxidative damage *in vivo* is limited in a healthy cell by the presence of defense mechanisms that operate with faster kinetics than oxidative reactions. This is the case for membrane antioxidants such as tocopherol;^368^ detoxification by aldoketoreductases, glutathione peroxidases, and glutathione *S*-transferases;^133,601^ and quenching of hydroperoxyl radicals by superoxide dismutase.^327^

In model systems *in vitro*, oxidation products accumulate on a time scale of minutes to hours.^397,455,489^ This is a similar time scale to cell death mediated by ferroptosis, giving a clear indicator of the rate by which toxic levels of oxidized lipid products can accrue when unchecked.^602^ However, the progression of diseases where lipid oxidation and an accumulation of damaged lipids is a factor, such as atherosclerosis,^126,603^ can be months to years. Alongside the generation of lipid oxidation products in disease conditions, the increased formation of reactive aldehydes leads to the generation of protein adducts that are both detectable and characteristic of the disease in question.^133,404^ In postmitotic cells, such as lens fiber cells, adaptations to reduce oxidative damage include a high cholesterol level and increased levels of saturated acyl chains.^604^ Oxidative damage to lipids does accrue in these cells, but even in aged individuals the levels can be surprisingly low.

As outlined above, some proteins have very slow turnover rates, and it may be expected that these proteins will accumulate modifications by reactive electrophiles derived from lipids over time. In this context it is notable that aquaporin-0 from both human and bovine lenses is lipidated with acyl chains in two positions that present a fatty acid profile that is similar to the membrane leaflet in which the protein resides, suggesting that these post-translational modifications have occurred by direct transfer from the lipid.^303^

Many of the processes by which membrane lipids react exhibit an interdependence. For example, reactive oxygen species, including hypochlorous acid, are able to cause the formation of lysolipids, which can then trigger an inflammatory response.^597^ By contrast, an accumulation of oxidatively damaged lipids facilitates water penetration to greater depths in the membrane, with subsequent increases in the rates of transbilayer lipid diffusion and hydrolysis and greater penetration of soluble oxidants.^506^

### 6.3 Final Comments

From the large body of work available on lipid reactivity, we now have a better understanding of the many chemical processes that impact membrane lipids.

First, lipids are susceptible to lysis reactions at the glyceryl ester groups involving nucleophilic attack on the ester carbonyl by water (hydrolysis) or organic nucleophiles. The byproducts are a lysolipid and either a free fatty acid or a lipidated product. Lysolipids can in turn be subjected to further hydrolysis to form *sn*-glycero-3-phosphocholine (GPC). Hydrolysis of lipids and membrane lipids can be affected by temperature (slower rates at low temperatures), pH (slowest rates at pH 5.8–6.5), and their chain length (higher rates for shorter chains and increased unsaturation levels). Some membrane additives, such as ascorbyl palmitate, dialkylphosphates, or cationic amphiphilic drugs (CADs), can also influence hydrolysis rates. In the case of aminolysis involving amine nucleophiles, reactivity is impacted by the p*K*_a_ of the ammonium form of the amine. For both aminolysis and transesterification reactions, the disposition of the reactive group in the membrane-bound form of the nucleophile has a significant effect on reactivity. These lytic reactions, which potentially lead to the formation of multiple byproducts, can severely impact the biophysical propertied and long-term stability of lipid membranes.

Second, lipids are well documented to undergo oxidation processes, the most common being autoxidation of the alkene groups of unsaturated fatty acids and sterols through a multistep process involving triplet oxygen. Oxidation can also occur with singlet oxygen and by reaction with other oxidizing agents such as hypochlorous acid. A key biophysical effect of these oxidation reactions is to increase the depth of water penetration into lipid membranes. Biological effects are associated with disturbances to lipid homeostasis and the triggering of apoptotic pathways such as ferroptosis. Oxidative damage is particularly associated an increased risk of cardiovascular disease.

Finally, lipids are affected by the isomerization of the alkene from *Z* to *E* through a radical addition–elimination reaction. This happens in the presence of sulfur- and nitrogen-centered electrophilic radicals, with sources such as cysteine, methionine, or glutathione. Isomerization through thiyl radicals has also been demonstrated in membrane lipids. (*E*)-Alkenes have been shown to decrease the fluidity and increase the transmembrane mobility and permeability of membrane lipids.

## Abbreviations

4-HNE: 4-hydroxynonenal
4-ONE: 4-oxononenal
AAPH: 2,2′-azobis(2-methylpropionamidine)dihydrochloride
AGE: advanced glycation end product
AP: ascorbyl palmitate
AQP0: aquaporin-0
BDMP: 4-*tert*-butyl-2,6-dimethylphenol
BLM: bleomycin
CAD: cationic amphiphilic drug
CDL: cardiolipin
Cer: ceramide
chol: cholesterol
DBHN: di-*tert*-butylhyponitrite
DEPC: 1,2-dielaidoyl-*sn*-glycero-3-phosphocholine
dFdC: gemcitabine
DFT: density functional theory
DHC: 7-dehydrocholesterol
DHPC: 1,2-dihexanoyl-*sn*-glycero-3-phosphocholine
DMPC: 1,2-dimyristoyl-*sn*-glycero-3-phosphocholine
DMPG: 1,2-dimyristoyl-*sn*-glycero-3-phosphoglycerol
DOPC: 1,2-dioleoyl-*sn*-glycero-3-phosphocholine
DOPE: 1,2-dioleoyl-*sn*-glycero-3-phosphoethanolamine
DOTAP: 1,2-dioleoyl-3-trimethylammonium propane
DPPA: 1,2-dipalmitoyl-*sn*-glycero-3-phosphatidic acid
DPPC: 1,2-dipalmitoyl-*sn*-glycero-3-phosphocholine
DPPE: 1,2-dipalmitoyl-*sn*-glycero-3-phosphoethanolamine
DPPG: 1,2-dipalmitoyl-*sn*-glycero-3-phosphoglycerol
DPPlsC: 1,2-di-O-((*Z*)-1′-hexadecenyl)-*sn*-glycero-3-phosphocholine
DSPC: 1,2-distearoyl-*sn*-glycero-3-phosphocholine
DSPE: 1,2-distearoyl-*sn*-glycero-3-phosphoethanolamine
DSPG: 1,2-distearoyl-*sn*-glycero-3-phosphoglycerol
EIC: extracted ion current
ELF: epithelial lining fluid
EPC: hen egg phosphatidylcholine
EPG: hen egg phosphatidylglycerol
EPR: electron paramagnetic resonance
FFA: free fatty acid
GPC: *sn*-glycero-3-phosphocholine
GUV: giant unilamellar vesicle
In: initiator
In•: initiating radical
H-EPC: hydrogenated hen egg phosphatidylcholine
H-soyPC: hydrogenated soybean PC
HEPES: hydroxyethylpiperazine ethanesulfonic acid
LPC: lyso-phopshatidylcholine
L_d_: liquid disordered
L_o_: liquid ordered
MALDI: matrix-assisted laser desorption/ionization
MECH: 3-(2-mercapthoethyl)quinazolin-2,4(1*H*,3*H*)-dione
MeO-AMVN: 2,2′-azobis(4-methoxy-2,4-dimethylvaleronitrile)
MPO: myeloperoxidase
MUFA: monounsaturated fatty acid
NIR: near-infrared
OHC: hydroxycholesterol
PA: phosphatidic acid
PazePC: 1-palmitoyl-2-azelaoyl-*sn*-glycero-3-phosphocholine
PC: phosphatidylcholine
PCpl: plasmenylcholine
PE: phosphatidylethanolamine
PEG: polyethylene glycol
PEPC: 1-palmitoyl-2-elaidoyl-*sn*-glycero-3-phosphocholine
PEpl: plasmenylethanolamine
PG: phosphatidylglycerol
PGPC: 1-palmitoyl-2-glutaryl-*sn*-glycero-3-phosphocholine
PH-eggPC: partially hydrogenated hen egg PC
PH-soyPC: partially hydrogenated soybean PC
PI: phosphatidylinositol
PLA/B/C/D: phospholipase A/B/C/D
POPC: 1-palmitoyl-2-oleoyl-*sn*-glycero-3-phosphocholine
POVPC: 1-palmitoyl-2-(5-oxovaleroyl)-*sn*-glycero-3-phosphocholine
PoxnoPC: 1-palmitoyl-2-(9′-oxo-nonanoyl)-*sn*-glycero-3-phosphocholine
PS: phosphatidylserine
PTM: post-translational modification
PUFA: polyunsaturated fatty acid
PV: peroxide value
ROS: reactive oxygen species
RNS: reactive nitrogen species
SAPC: 1-stearoyl-2-arachidonyl-*sn*-glycero-3-phosphocholine
SM: sphingomyelin
SMase: sphingomyelinase
SOD: superoxide dismutase
SLPE: 1-stearoyl-2-linoleoyl-*sn*-glycero-3-phosphoethanolamine
SLPG: 1-stearoyl-2-linoleoyl-*sn*-glycero-3-phosphoglycerol
SLPS: 1-stearoyl-2-stearoyl-*sn*-glycero-3-phosphoserine
SOMO: singly occupied molecular orbital
SP-C: surfactant protein C
SPC: sphingosylphosphorylcholine
TBARS: thiobarbituric acid reactive substances
*T*_m_: gel to liquid crystalline phase transition temperature
α-TOC: α-tocopherol
